# Transfer RNA-derived fragments in aging *Caenorhabditis elegans* originate from abundant homologous gene copies

**DOI:** 10.1038/s41598-021-91724-z

**Published:** 2021-06-10

**Authors:** GiWon Shin, Hee Jung Koo, Mihwa Seo, Seung-Jae V. Lee, Hong Gil Nam, Gyoo Yeol Jung

**Affiliations:** 1 Institute of Environmental and Energy Technology, Pohang University of Science and Technology, Pohang, Gyeongbuk South Korea; 2 School of Interdisciplinary Bioscience and Bioengineering, Pohang University of Science and Technology, Pohang, Gyeongbuk South Korea; 3 Department of Chemical Engineering, Pohang University of Science and Technology, Pohang, Gyeongbuk South Korea; 4 Department of Biological Sciences, Korea Advanced Institute of Science and Technology, Daejeon, South Korea; 5 Center for Plant Aging Research, Institute for Basic Science, Daegu, South Korea; 6 Present Address: Department of New Biology, Daegu Gyeongbuk Institute of Science and Technology, Daegu, South Korea

**Keywords:** Gene expression, Ageing

## Abstract

Small RNAs that originate from transfer RNA (tRNA) species, tRNA-derived fragments (tRFs), play diverse biological functions but little is known for their association with aging. Moreover, biochemical aspects of tRNAs limit discovery of functional tRFs by high throughput sequencing. In particular, genes encoding tRNAs exist as multiple copies throughout genome, and mature tRNAs have various modified bases, contributing to ambiguities for RNA sequencing-based analysis of tRFs. Here, we report age-dependent changes of tRFs in *Caenorhabditis elegans*. We first analyzed published RNA sequencing data by using a new strategy for tRNA-associated sequencing reads. Our current method used unique mature tRNAs as a reference for the sequence alignment, and properly filtered out false positive enrichment for tRFs. Our analysis successfully distinguished de novo mutation sites from differences among homologous copies, and identified potential RNA modification sites. Overall, the majority of tRFs were upregulated during aging and originated from 5′-ends, which we validated by using Northern blot analysis. Importantly, we revealed that the major source of tRFs upregulated during aging was the tRNAs with abundant gene copy numbers. Our analysis suggests that tRFs are useful biomarkers of aging particularly when they originate from abundant homologous gene copies.

## Introduction

Transfer RNAs (tRNAs) are essential for mRNA translation, whose integrity is maintained with high fidelity^1–3^. Interestingly, recent studies using the next generation sequencing (NGS) technology indicate that tRNA-derived fragments (tRFs) are generated in various circumstances^4–6^. These tRFs are not likely randomly degraded products, because their aligned sequences are not evenly distributed but rather localized to specific regions of tRNA genes. The enrichment has been observed in many biological systems, including cancer cell lines^6–8^, virus-infected cells^9^, adipose tissue-driven stem cells^10^, mouse sera^11^, plants^12^, nematodes^13^, bacterial community^14^, and halophilic archaea^15^. These studies strongly indicate that tRNA fragmentation is an evolutionary conserved phenomenon. Recent studies also suggest that several tRFs play regulatory roles as small RNAs^6,16^. Nevertheless, the functional significance of tRFs remains largely unknown.

tRFs are classified into two main classes: tRNA halves and small tRNA fragments, both of which can be further subcategorized based on cleavage sites. Four potential cleavage sites exist in mature tRNAs, all of which are located at exposed single-stranded loops. For example, tRNA halves are produced by cleavage at the anticodon loops (AC-loops), resulting in 30 to 35 nucleotide-long RNA fragments^17^. Fragments derived from mature tRNAs or pre-tRNA transcripts are generated in various physiological or pathological contexts, such as disease conditions^8^ and stress responses^18^. Interestingly, some tRFs are accumulated during aging in *C. elegans*^13^ and in mammals^11^. In addition, dietary restriction, an established anti-aging regimen, counteracts age-dependent changes in the level of tRNA halves in mouse sera^11^. These studies raise a possibility that tRFs play a role in aging processes.

Despite these intriguing functions, several characteristics of tRNAs hindered proper analysis of tRNA species, including tRFs, using RNA sequencing data. First, tRNA genes are present in multiple copies throughout genome^19^; some tRNA genes are identical and others are very similar in sequences. Thus, it is challenging to identify exact genomic origins of tRF sequence reads. Second, tRFs can be processed from both pre-tRNA transcripts and mature tRNAs^4^. Because mature tRNAs are extensively modified^5^, tRFs generated from mature tRNAs may not precisely match corresponding coding sequences. For example, 24 genomic regions in *C. elegans* encode proline-TGG tRNAs, and nine additional regions have highly homologous sequences. If a sequencing read is generated from one of these copies, a general way for mapping will be just assigning one or several regions randomly among these matches. Further complications arise because of base modifications that occur frequently in mature tRNAs, which can change complementary base pairings.

In this study, we report age-dependent changes of tRFs in *C. elegans*. We analyzed a previously published sequencing data^13^ by using a new strategy for tRNA-associated sequencing reads. We successfully distinguished de novo mutation sites from differences among homologous copies, and identified potential RNA modification sites. We showed that the majority of tRFs were upregulated during aging and originated from 5′-ends, which we validated by using Northern blot analysis. Importantly, we found that the major source of these upregulated tRFs was tRNA genes having abundant homologous copies across the genome.

## Results

### tRNA fragments are mostly derived from mature tRNAs

**Figure 1:**
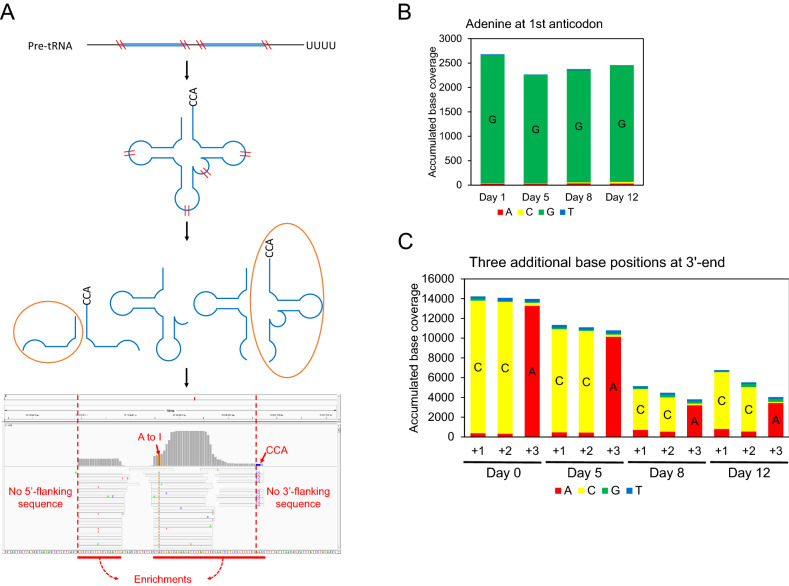
Characteristics of tRFs identified by sequencing read alignment to the genome reference. (**A**) Example of sequencing read alignment to a tRNA gene locus is shown with a brief illustration of tRF generation via tRNA maturation. The sequencing read alignment suggests that the majority of tRFs are derived from mature tRNAs. Upon transcription, pre-tRNA has 5′ and 3′ margins as well as an intron, which are removed later with the maturation process. In addition, 3′-CCA and RNA base modifications observed in the sequencing reads are characteristics of mature tRNA. Dashed vertical lines at the integrative genomics viewer (IGV) plot indicate the start and the end of the tRNA gene. (**B**) Accumulated base coverage at the first position of the anticodons starting with adenine. The base coverage for every age is shown as a bar graph. For 56 first anticodon base loci, for which the genomic sequence is adenine, the sequenced bases were counted per read alignment. (**C**) Accumulated base coverage at the three additional base positions of all tRNA genes. For the three additional 3′ base positions of 600 tRNA genes (excluding pseudogenes), the sequenced bases were counted per read alignment. For both panels (**B**) and (**C**), adenine (A), cytosine (C), guanine (G), and thymine (T) bases are indicated by red, yellow, green, and blue colors, respectively. The height of colored bar indicates the number of sequencing reads with a corresponding base at each locus.

A previous small RNA sequencing analysis indicates that sequencing reads that match parts of annotated tRNA gene regions are accumulated during aging in *C. elegans*^13^. We determined whether the RNA sequencing reads originated from mature tRNAs or pre-tRNAs by thoroughly assessing the characteristics of tRNA-related reads. For all the 820 regions listed in a genomic tRNA database (http://gtrnadb.ucsc.edu/), the number of aligned reads per million was 8103, 8884, 13,005, and 18,934 respectively for days 0, 5, 8 and 12 of ages. We found that 98% of the reads were aligned within the boundary of tRNA genes (Table 1 and Supplementary Table S1), whereas remaining 2% included sequences that did not originate from the tRNA genes (i.e. the flanking sequences). The sequences of mature tRNAs are different from those of tRNA gene sequences mainly because of three following post-transcriptional modifications (Fig. 1A). First, some tRNA genes include introns that are not present in mature tRNAs after splicing. Second, RNA base editing during tRNA processing is reflected in the sequencing analysis. Third, the CCA trinucleotide added at the 3′-ends of mature tRNAs is different from the sequence of tRNA genes. In particular, the RNA base editing events can be identified upon inspecting the mapped reads. Adenine (A) to inosine (I) editing can be identified by sequencing analysis because the modified base I prefers cytosine (C) as its complementary binding partner instead of thymine (T)^20^. We found that more than 97% of the A’s in the first anticodons were changed to guanines (G’s) throughout all ages (Fig. 1B). In addition, we observed zero or one mean read alignments for 32 intronic regions of tRNA genes (Table 1 and Supplementary Table S2). Moreover, the majority of sequencing reads mapped at the 3′-ends of genomic tRNA regions had the CCA trinucleotides (Fig. 1C). Together, these results indicate that the majority of tRFs originated from mature tRNAs.

**Table 1:** tRNA-associated reads from small RNA sequencing.

Age	Day 0	Day 5	Day 8	Day 12
Total aligned read	5,185,655	6,824,397	6,361,538	7,429,646
Read aligned to tRNA genes (% of total aligned read)	42,018 (0.81%)	60,630 (0.89%)	82,732 (1.30%)	140,672 (1.89%)
Read aligned within gene boundary, % of total tRNA read*	98%	98%	98%	98%
Mean read alignment to introns**	0	0	1	1

*Supplementary Table S1 shows number of aligned reads in each tRNA gene.

**Supplementary Table S2 shows number of aligned reads in each intronic region.

Interestingly, the trends of base coverage by sequencing reads throughout different ages were different among the entire tRNA genes, anticodons, and the three additional bases at the 3′ends. Among the total aligned sequencing reads from four different time points, 0.8%, 0.9%, 1.3%, and 1.9% were aligned to the tRNA genes, indicating a gradual increase in tRNA-originated fragments (Table 1). However, the reads aligned to the three-base anticodons did not match this trend, and maintained at a similar level throughout all four ages (Fig. 1B). In addition, the sequencing reads aligned to three additional bases at the 3′-end of tRNA genes (i.e. the positions corresponding to CCA trinucleotides of mature tRNAs) decreased with age (Fig. 1C). These data suggest that the expression of distinct types of tRFs is regulated differently during *C. elegans* aging.

### Strong correlation between genomic copy number and tRF abundance at old ages

**Figure 2:**
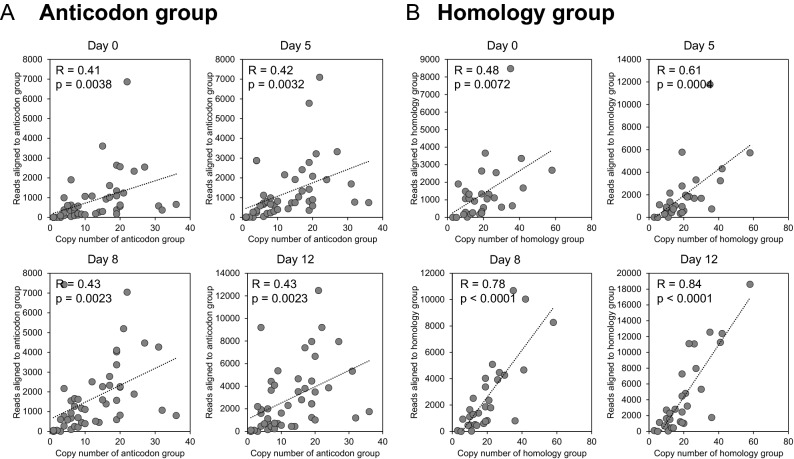
Correlation between tRF abundance and genomic copy number of homologous tRNA genes. For all four ages (days 0, 5, 8 and 12), tRF abundance and the tRNA gene copy number were compared with two different definitions for homologous tRNA gene groups. The copy number was determined based on the definitions of gene group. (**A**) Correlation based on anticodon. The number of sequence reads aligned to tRNA genes with a same anticodon (y-axis) is plotted against genomic frequency of the anticodon (x-axis). In this analysis, anticodon frequency represents the dosage of tRNA genes in each homologous group. (**B**) Correlation based on sequence homology among tRNA sequences. Unique tRNA sequences are binned into 30 homology groups. For each homology group, the genomic copy number (x-axis) and the number of sequencing read alignment to the homologous genomic copies (y-axis) are plotted. For each scatter plot, dotted line indicates linear regression, and the Pearson correlation (R) is provided with the significance (*p*).

In *C. elegans* genome, 605 tRNA genes are present for decoding 20 amino acids. After eliminating pseudogenes and identical copies, 178 distinct tRNA gene sequences remained (Supplementary Table 3). Among the 64 possible anticodons, the *C. elegans* genome contains tRNA genes for 48 anticodons (Supplementary Table 4). By categorizing these sequences with anticodons, we found that the copy number of homologous genes weakly correlated with the number of sequencing read alignments in all four ages (Pearson correlation R = 0.41–0.43; Fig. 2A). The number of read alignments represents the abundance of tRFs originating from the anticodon group, and the weak correlation suggests homology across different anticodon groups.

To define the homology groups of tRNA genes, we performed multiple sequence alignments of the 178 distinct tRNA gene sequences. Using the percent identity matrix provided by Clustal Omega software (https://www.ebi.ac.uk/Tools/msa/clustalo/), we clustered the tRNA sequences into homology groups (Supplementary Fig. 1A). When we chose a specific number of tRNA sequence clusters (e.g. k = 30), we found that the gene copy number displayed stronger correlations with tRF abundance than when tRNA genes were grouped by anticodons (Pearson correlation R = 0.48–0.84; Fig. 2B). We chose the cluster number to maximize the correlations while maintaining tRNAs of all the amino acids in separate clusters (Supplementary Fig. 1B). Interestingly, the correlation increased with age regardless of the selected number of clusters (Supplementary Fig. 1B). For example, by using the 30 clusters, the correlation was the strongest at day 12 of age (R = 0.84, *p* < 0.0001; Fig. 2B). The homology groups with higher copy numbers produced more tRFs in old ages than the groups with lower copy numbers. Given the strongest correlation at day 12 of adulthood, the high sequence copy number may play important roles in old ages. In addition, the strong correlation suggests that the major source of tRFs are not the parts specific to a tRNA species or a certain anticodon group, but homologous parts that are shared within a homology group.

### Re-alignment using modified reference sequences for measuring age-dependent quantitative changes of individual tRFs

**Figure 3:**
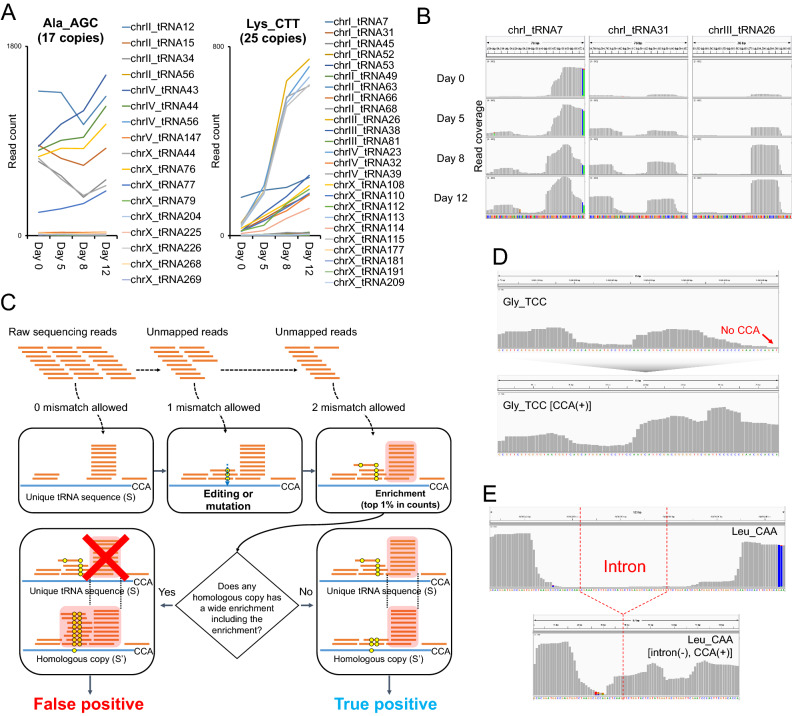
Improved tRF identification using unique mature tRNA sequences. (**A**) Non-correlative quantitative changes among reads mapped to identical gene copies. For identical gene copies of Ala_AGC tRNA (17 copies) and Lys_CTT tRNA (25 copies), count of reads mapped to each individual gene is shown. (**B**) Different enrichment patterns and quantitative changes of reads mapped to identical gene copies. For three of 25 identical Lys_CTT tRNA gene copies, per-base read coverage is shown. Grey bars indicate sequenced bases matching the reference, and colored bars indicate mismatches. Green, brown, blue and red colors indicate adenine, guanine, cytosine, and thymine, respectively. The scale of y-axis is shown at the top left of each plot. (**C**) Schematic illustration of new sequence alignment strategy. Using 178 unique tRNA sequences as reference, a series of sequence alignments are performed with allowing zero, one, and then two mismatches. The process detects gene mutation or RNA base editing among homologous gene copies. For the enrichment, of which the abundance is among the top 1%, additional filtering is performed. Only when an enrichment exists as is in all homologous gene copies, the process determines true positive hits. (**D,E**) Comparison between previous and new alignment results. Per-base read coverage is compared between a tRNA gene and the sequence of matured tRNA (i.e., with 3′-CCA or without intron sequences). Grey bars indicate sequenced bases matching the reference, and colored bars indicate mismatches. Green, brown, blue and red colors indicate adenine, guanine, cytosine, and thymine, respectively.

We then sought to determine the quantitative changes of individual tRFs during aging. As described above, we were not able to align some of the raw sequence reads from mature tRNAs to the genomic regions of tRNAs. Consequently, this analysis using the genomic sequence as a reference may cause false identification of the enrichment of tRFs, and may not precisely reflect the quantitative changes of tRFs. For example, even when tRNA gene copies shared identical sequences, mapped read counts and their enriched regions did not correlate with each other (Fig. 3A,B). Furthermore, the existence of homologous gene copies containing only a few bases of sequence differences and RNA editing events in mature tRNAs complicated the analysis of age-dependent quantitative changes of tRFs. Thus, we re-aligned the raw sequence reads to modified reference sequences by considering mature forms of tRNAs and their existence as multiple homologous gene copies (Fig. 3C). The new reference included all possible mature forms of unique tRNA sequences that contained the 3′-end CCA sequences without introns (Supplementary Table S3). To identify all potential RNA editing events while distinguishing gene-specific base differences and edited sites, we employed sequential alignment methods. We aligned only perfect matches at the first step, and then allowed mismatched alignments at following steps. By using this strategy, we were able to avoid false positive mutation calls. The identified mismatches must have originated from either RNA editing events or genomic mutations. Instead of randomly reporting one of the best hits, which may cause unequal distribution of alignments among homologous copies, we reported all the best hits as primary alignments in the new results. Upon identification of significantly enriched regions, we further inspected the sequences to rule out false positive hits for tRFs. We then selected the regions for final candidates only when no homologous gene included a same enrichment as part of another enrichment that is bigger in size (Fig. 3C).

Because our alignment strategy considered the characteristics of tRNAs, we were able to recover previously unmapped reads. Some of the previously identified tRFs are likely to be falsely annotated because of these failed alignments. We found that the alignment of genomic tRNAs did not represent precise quantitative levels of tRNA fragments because of 3′-CCA addition in mature tRNAs (Fig. 3D). We fixed this error by adding the CCA sequences to the 3′-ends of tRNAs. Moreover, for spliced tRNAs, genomic tRNAs containing intron sequences between splicing junctions were not appropriate as references for analyzing tRFs that originated from mature tRNAs (Fig. 3E). The reads including the splicing junctions in middle regions did not align to genomic tRNA sequences, while being recovered by using mature tRNA sequences as references. Overall, these observations suggest that tRNA-originated reads need to be analyzed with sequences of mature tRNAs instead of genomic sequences as the reference.

### Identification of tRFs with age-dependent upregulation

**Figure 4:**
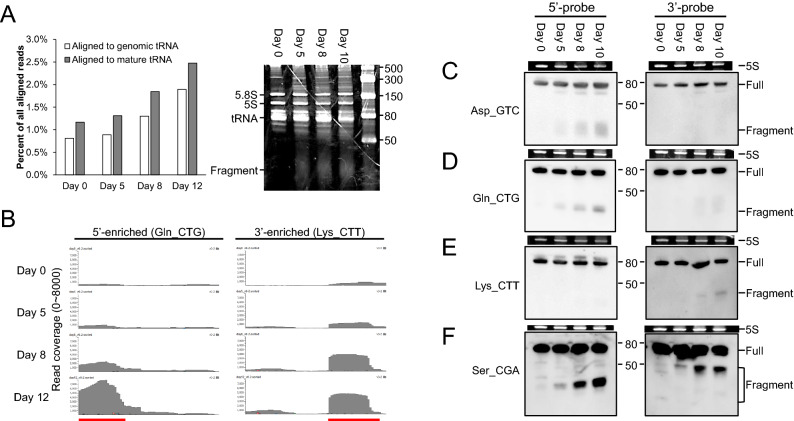
Types of tRF with different enrichment locations. (**A**) General trends of tRF abundance during aging. Percentage of reads mapped to tRNA sequences among all the sequencing reads (i.e. normalized tRF abundance) is shown for both alignments to genomic and mature tRNA sequences (left panel). Gel electrophoresis analysis indicates smear bands smaller than 50 bases as well as the bands for full length tRNA and 5S and 5.8S rRNAs (right panel). The intensity of the smear bands representing tRFs increases during aging. (**B**) Two most frequent types of tRF enrichment (5′-enriched and 3′-enriched). Examples of two most frequent enrichment types are shown. X- and Y-axes indicate read coverage and the base positions in each mature tRNA sequences. Red bar at the bottom indicates the regions with enrichment. (**C-F**) Northern blot hybridization results. Using probes complimentary to both 5′- and 3′-end regions of four tRNA sequences, five tRF detected by the sequencing analysis were validated. The last two tRF were detected from a same tRNA sequence (Ser_CGA). For each Northern blot result, 5S rRNA band is shown as a loading control. The original blot images as well as the gel images taken before transfers are provided in Supplementary Information file.

From each of four time points (days 0, 5, 8, and 12), we selected tRF enrichment with the top 1% abundance, and then identified false positives by using sequence alignment (Supplementary Table S5). General trends of sequencing reads mapped to the tRNA reference sequences suggest that tRF population increases during aging. We confirmed this observation by detecting the presence of a smear band below the size range of tRNAs (Fig. 4A). We categorized three enrichment types of tRFs based on their relative locations in mature tRNAs: 5′-end, 3′-end, and middle fragments (Supplementary Table S6). The majority (49) of total 57 tRFs identified in our analysis occurred at either ends of tRNAs (25 at the 5′-ends and 24 at the 3′-ends; Fig. 4B). In particular, the majority of 5′-end tRFs (18 out of 25) displayed a general trend of age-dependent upregulation. In contrast, 3′-end tRFs exhibited mixed patterns. Interestingly, the levels of 3′-end tRFs with the CCA sequences displayed decreasing patterns during aging, whereas the ones without the CCA sequences exhibited increasing patterns. We validated five abundant tRFs, 5′-Asp_GTC, 5′-Gln_TTG, 5′-Ser_CGA, 3′-Lys_CTT, and 3′-Ser_CGA tRFs, by using four different Northern blot assays (Fig. 4C–F); one assay targeted two Ser_CGA tRFs that originated from 5′-end and 3′-end of the tRNA (Fig. 4F). Each of the other three assays targeted one tRF and a region without sequencing read enrichment, which was used as a control (Fig. 4C–E). We included both 3′-end tRFs with and without the CCA sequences in the validation set. All four tRFs that we identified as age-dependently upregulated ones by performing sequencing analysis exhibited an increasing pattern during aging in our Northern blot analysis as well (Fig. 4C–F). However, we did not detect 3′-Ser_CGA, an age-dependently downregulated tRF with the CCA sequence based on the sequencing analysis, using Northern blotting (Fig. 4F). Instead, we identified a 50-base pair (bp) tRF that was upregulated with age in the Northern blot data (Fig. 4F). The sequencing analysis targeted small RNA species with a size up to 35 bp, and this may be the reason why our sequencing analysis missed the 50-bp tRF. Thus, our validation with Northern blot analysis raises the possibility that age-dependently downregulated 3′-end tRFs with CCA sequences are likely to be artifacts from sequencing analysis. Together with the RNA seq analysis, our results suggest that age-dependent upregulation of tRFs is prevalent among *C. elegans* tRFs.

## Discussion

The most significant advance achieved in the current study is that our new analysis revealed that the major source of tRFs in worms with old ages (e.g. days 8 and 12) was the tRNA genes with high copy numbers. To be precise in counting copy numbers of genes homologous to each other across different anticodons and amino acids, we grouped tRNA gene sequences by their sequence homology. After clustering unique tRNA sequences into homology groups, tRF abundance displayed a strong correlation with the number of genomic copies of each group. In particular, the correlation was higher in the older ages (i.e., days 8 and 12) than in the younger ages (i.e., days 0 and 5) (Fig. 2B), regardless of the number of clusters chosen for the comparison (Supplementary Fig. 1B). The abundant gene copies of tRNA are common in eukaryotic genomes, and have been considered redundant^19^. However, our analysis strongly suggests that the genomic copies of tRNAs are not redundant, but useful as the source of tRFs in the aging process. Although a direct association between tRFs and human aging has not been reported yet, tRFs have been shown to be associated with age-related human diseases, such as cancers and neurodegenerative diseases^21^. Particularly, accumulation of tRFs is observed not only in cells, but also in extracellular environments^22^, suggesting potential of tRFs as promising diagnostic markers^22^. For identifying novel tRF markers, we suggest that abundance of genomic sources, i.e. the genomic copy number, should be considered when prioritizing candidate markers for clinical studies.

For individual tRFs that are accumulated during the aging, our analysis showed that the tRFs can be be categorized by the relative locations of their origins in the corresponding tRNAs. From a given mature tRNA, the biogenesis of tRFs essentially generates both 5′-tRF and 3′-tRF^23^, but generally the abundance did not match in our analysis (Supplementary Table 6). In other words, from a tRNA, either 5′- or 3′-tRF was produced. A previously reported meta-analysis of more than 50 small RNA sequencing libraries consistently observed the unequal abundance between 5′- and 3′-tRFs from a same tRNA origin^24^. In our study, approximately a half of the identified tRFs originated from the 5′-sides. Almost all these 5′-tRFs displayed an age-dependent increase in their levels. In contrast, our sequencing analysis indicated that 3′-tRFs exhibited no consistent age-dependent changes in their levels, i.e. both upregulation and down-regulation during aging. However, a validation by Northern hybridization suggested that the down-regulated 3′-tRFs may be artifacts from sequencing. Interestingly, the decreasing expression pattern was observed only among 3′-tRFs with the terminal CCA sequences. One possibility is that modifications added specifically to these tRFs during aging may be inhibitory to the enzymatic reactions in the RNA sequencing library preparation. When excluding the tRFs containing terminal CCA, 3′-tRFs also showed an age-dependent upregulation. Therefore, regardless of the tRF types, upregulation was most frequent during aging. In addition, for many of these upregulated tRFs, the abundance had a linear correlation with the age, and therefore tRFs can be used as quantitative biomarkers of aging.

Our findings are based on aging time course data derived from single samples taken at four different ages (i.e., days 0, 5, 8, and 12 of adult stage). To validate the data, we performed additional experiments with an independent set of samples also obtained at four different ages (i.e., days 0, 5, 8, and 10 of adult stage). The gradual increase in overall tRF population during aging was also observed in our validation (Fig. 4A). Moreover, for a subset of tRF species with a high genomic copy number, we individually confirmed their age-dependent accumulation (Fig. 4C–F). However, future studies with more replicates and independent experimental setups will further validate our findings. For example, carefully setting up conditions without additional stresses (e.g. starvation and contamination by non-*E. coli* bacteria) will be able to rule out the possibilities of the inadvertent effects of environmental factors. In addition, studies with other eukaryotic systems will confirm if the associations are evolutionarily conserved.

The number of studies reporting biological activities of tRFs is growing, but all of these studies suffered from frequent nucleotide modification and sequence homology among different tRNA species and isoforms^21^. When analyzing small RNA sequencing data, these characteristics should be considered for the quantification of individual tRFs. A software package, MINTmap, has been developed for the identification of tRFs from RNA sequencing data^25^, but this method only considers the sequence homology problem, not the nucleotide modifications. MINTmap identifies tRFs by using only exact sequence match, which excludes tRFs with modified RNA bases. In contrast, in our study, we considered not only the sequence homology problem, but also the modified RNA bases.

Identification of the modification site was possible by a series of sequence alignments prioritizing perfect match, one mismatch, and then two mismatches (Fig. 3C). From the identified RNA base modification events, our results indicate no significant change in the modification patterns during aging. Two major modification events that we consistently observed during aging were A to I and A to N^1^-methyl-adenosine (m^1^A) changes; these two types of modifications are generally detected at the first nucleotides of anticodon sites and in the T-loops, respectively. Because both of these changes are highly conserved in eukaryotes, these observations support the reliability of our analysis. Unlike the first type that displayed consistent base changes from A to G, m^1^A modifications can result in pairings with any of the four DNA bases when the source RNAs are reverse-transcribed^26,27^. Consistent with these results, the modifications can be detected by the existence of all four bases at a specific site in the T-loop (Supplementary Fig. 2). Identification of the modifications in transcriptome sequencing results has been reported^28,29^. In particular, the A-to-m^1^A modification is translated into different mismatch patterns depending on the nucleotide 3′ of m^1^A^29^. Our results also suggest the sequence-dependent mismatch patterns from m^1^A modifications. It will be important to determine the physiological role of these modifications in future research.

## Materials and methods

### Sequence data

The sequence data were downloaded from the website of modENCODE project (http://www.modencode.org/; study title: ‘changes in expression of small RNAs during aging in *C. elegans*’). The small RNA sequencing was performed on four different time points during adulthood of *C. elegans* (days 0, 5, 8 and 12 post-L4 molt; accession numbers: modENCODE_6503, modENCODE_6504, modENCODE_6505 and modENCODE_6506)^13^. Both fastq and sam files were downloaded and used for determination of tRF. For the time points with replications (days 0 and 8), we only used the first run for the analysis.

### Read and base coverage analyses

‘IntersectBed’ software^30^ was used for read coverage analysis of tRNA gene and intronic loci. IntersectBed with ‘-c’ option was run to count sequencing reads mapped to the coordinates obtained from a genomic tRNA database (http://gtrnadb.ucsc.edu/) with at least one base overlap (Supplementary Tables S1 and S2). Additionally, ‘-f 1’ option was used to count only the reads mapped completely within the boundary of tRNA genes. By subtracting the within-boundary count from the total count, the number of reads partially mapped to target gene loci was calculated (Supplementary Table S1). ‘Samtools mpileup’ software^31^ was used for base coverage analysis of specific site in tRNA genes (e.g. first anticodon site and three additional base positions). From all read mapped on a target site, the number of sequenced bases (i.e., A, G, T, or C) was counted. The full list of target base positions is provided in Supplementary Table S7.

### Sequencing read alignment

The adapter sequences were trimmed from raw sequences using ‘cutadapt’ software^32^. Small RNA sequencing reads, with the adaptor sequence trimmed, were retained if they were 15 nt or longer in length. The trimmed reads were first aligned to the mature tRNA sequences of *C. elegans* (Supplementary Table S3) using ‘Bowtie’^33^ with no mismatches (‘-v 0’ option) and all possible multiple alignments allowed (‘-a’ option). After the initial alignment with no mismatch allowed, the remaining reads were then sequentially aligned with one and two mismatches allowed (Fig. 3C).

### Northern hybridization

Eggs from bleached adult BA671 *spe-9(hc88) I* worms provided by Caenorhabditis Genetics Center, which is funded by the NIH National Center for Research Resources, were maintained at 15 °C on OP50-seeded NGM plates. Synchronized embryos by using bleaching were cultured at 23 °C to induce their sterility, and the worms were harvested at days 0 (pre-fertile), 5, 8 and 10 adult stages with M9 buffer, following a previous report^13^. Total RNAs were extracted from the day 1 adult worms using Trizol-based extraction methods (RNA Isoplus, Takara) and subsequently purified with an ethanol purification method. The extracted total RNA was separated by polyacrylamide gel electrophoresis (PAGE) using Novex TBE-Urea 15% gel system (Thermo Fisher Scientific Waltham, MA) following the recommended instruction. The separated RNA was electrophoretically transferred to a positively charged nylon membrane (Cat. No. 11209299001, Sigma-Aldrich, St. Louis, MO) using Trans-Blot SD Semi-Dry Transfer Cell (Bio-Rad Laboratories, Hercules, CA) at 60 V for 1 h. The nylon membrane and Whatman 3MM papers were soaked in 0.5X TBE buffer before assembled on the anode surface. The blotted RNA was crosslinked to membrane in a UV crosslinker. Oligonucleotide probes (Supplementary Table S8) were labeled with digoxigenin-11-dUTP (DIG-dUTP) using a DIG Oligonucleotide Tailing kit (Cat. No. 03353583910, Sigma-Aldrich). A terminal transferase catalyzes addition of DIG-dUTP and dATP to the 3′-end of oligonucleotide probes. By following the DIG application guideline provided by the manufacturer, the crosslinked membrane was prehybridized, hybridized with the DIG-labeled probes, and then washed. For detection of hybridized target probes, we added anti-DIG (Anti-Digoxigenin-AP, Cat. No. 11093274910, Sigma-Aldrich), and then a chemiluminescent substrate (CDP-Star, Cat. No. 12041677001, Sigma-Aldrich) as described in the guideline. Blot images were obtained by using ImageQuant LAS-4000 system (GE Healthcare, Chicago, IL).

## Supplementary Information

Supplementary Information.
