# Supplementary material for: Transfer RNA-derived fragments in aging Caenorhabditis elegans originate from abundant homologous gene copies
Source: Sci Rep. 2021 Jun 10;11:12304. doi: 10.1038/s41598-021-91724-z (PMC8192933; doi:10.1038/s41598-021-91724-z)
Supplement: Supplementary file 1 — Supplementary Information. [file 41598_2021_91724_MOESM1_ESM.pdf]

SUPPLEMENTARY MATERIALS

Transfer RNA-derived fragments in aging *Caenorhabditis elegans* originate from abundant homologous gene copies

GiWon Shin[1], Hee Jung Koo[2,5], Mihwa Seo[2], Seung-Jae V. Lee[4,*], Hong Gil Nam[5,6,*,†] and Gyoo Yeol Jung[2,3,*]

[1] Institute of Environmental and Energy Technology, [2] School of Interdisciplinary Bioscience and Bioengineering, [3] Department of Chemical Engineering, Pohang University of Science and Technology, Pohang, Gyeongbuk, South Korea

[4] Department of Biological Sciences, Korea Advanced Institute of Science and Technology, Daejeon, South Korea

[5] Center for Plant Aging Research, Institute for Basic Science, Daegu, South Korea

[6] Department of New Biology, Daegu Gyeongbuk Institute of Science and Technology, Daegu, South Korea

* To whom correspondence should be addressed. E-mail: seungjaevlee@kaist.ac.kr, nam@dgist.ac.kr, or gyjung@postech.ac.kr

[†] Current address: Department of New Biology, Daegu Gyeongbuk Institute of Science and Technology, Daegu, South Korea

A

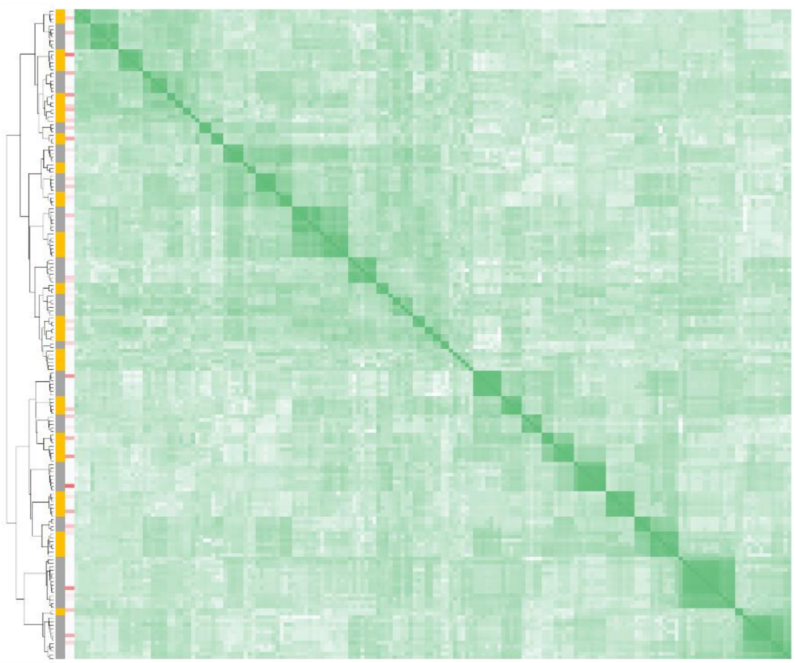

B

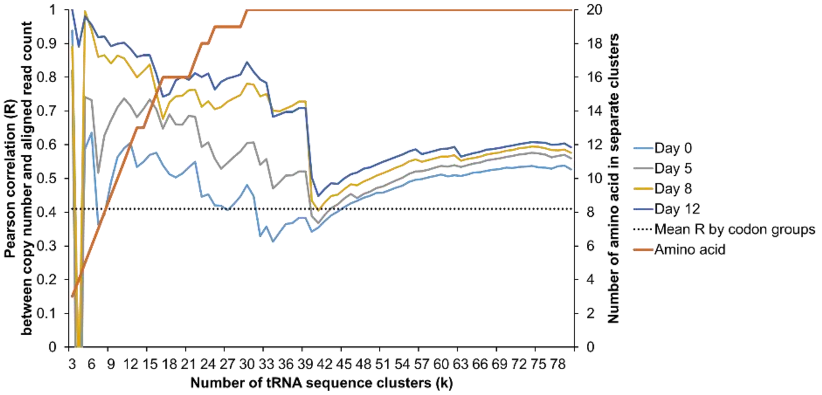

Supplementary Figure 1. Clustering of unique tRNA sequences. (A) Heat map of percent match scores from pairwise alignment. A 178x178 matrix with individual rows and columns indicating unique tRNA sequences is shown with two added columns at the left side. Diagonals in the matrix indicate the self-alignment from which the percent match score is 100. With the dendrogram from a hierarchical clustering, the first added column shows 30 different clusters with alternating colors. The second added column shows the number of genomic copies as heat map. **(B)** Correlation between tRNA sequence copy number and the number of aligned reads with different number of clusters (k). With this correlation analysis, k = 30 was chosen as the optimal number of clusters. The Pearson correlation (R) is shown for all ages at every k in a range of 2-80. Number of amino acids coded by tRNA sequences in separate clusters is also shown for each k. For k smaller than 30, not every amino acids are represented by one or more clusters. Among k of 30 or greater than 30, 30 resulted the greatest correlation at all ages. A dotted horizontal line indicates the correlation between codon-based tRNA copy number (i.e., the number of tRNA genes encoding the same anticodon) and the number of aligned reads (the mean value of the four ages).

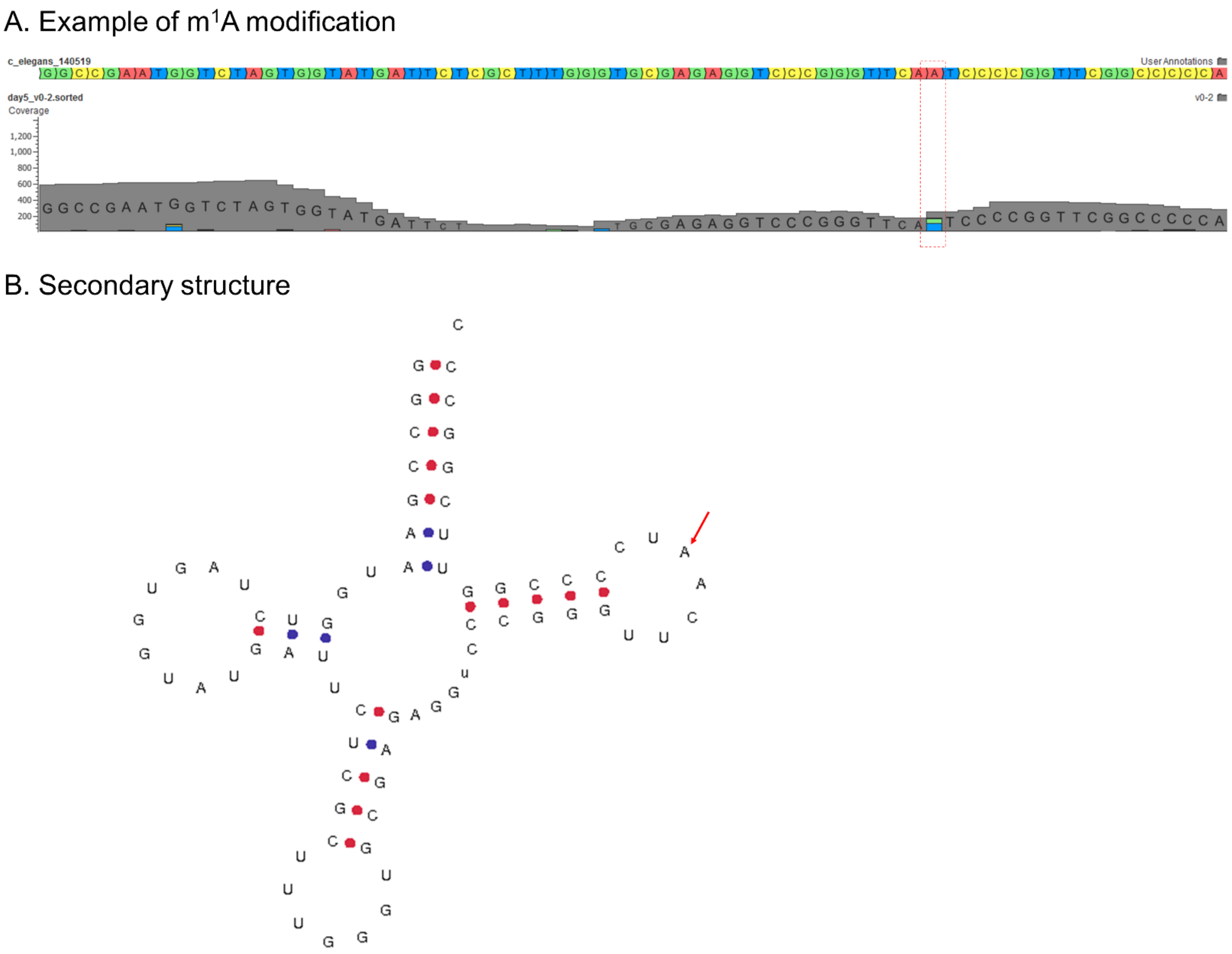

Supplementary Figure 2. N^1-methyladenosine (m^{1}A) RNA modification detected by sequencing. (A) Sequenced bases from an m^{1}A RNA modification site. The m^{1}A modification site is indicated by a red dotted box. Sequences of tRNA gene are shown at the top, and the per-base coverage is shown at the bottom. At each bar, grey color indicates sequenced base matching the gene sequence. Red, green, yellow, and blue colors indicate mismatches by A, G, C, and T bases, respectively. For the m^{1}A modification, in addition to the matching A base, sequencing generated signals G and T bases because of mispairing during the reverse transcription. **(B)** Predicted secondary structure of tRNA with three loops. Red dot indicates G-C pairing, and blue dot indicates A-U or G-U pairings. The third loop (T-loop) has the m^{1}A RNA modification (red arrow).

Original Northern blot images

Figure 4C gel before transfer (loading control)

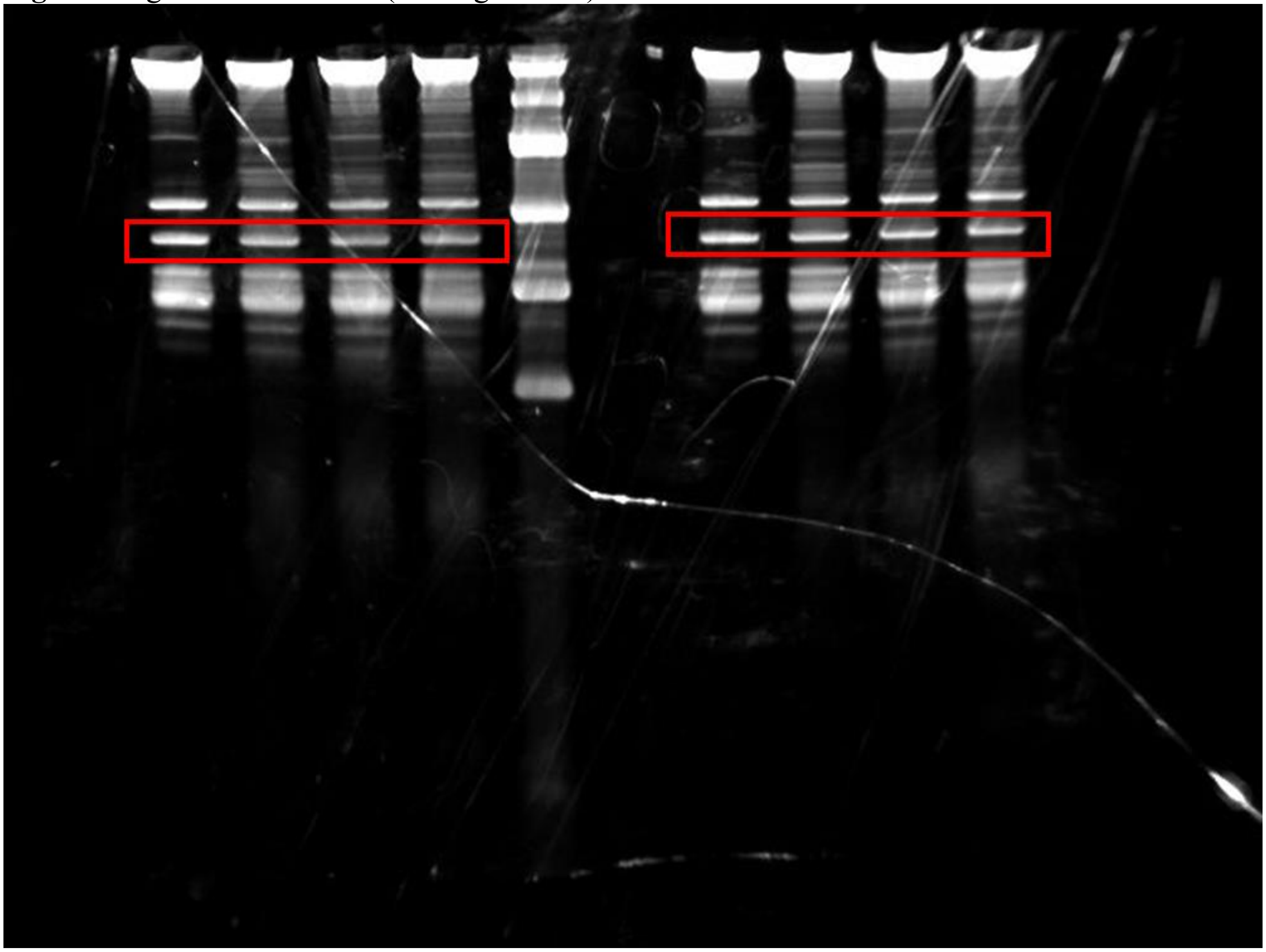

Figure 4C left panel

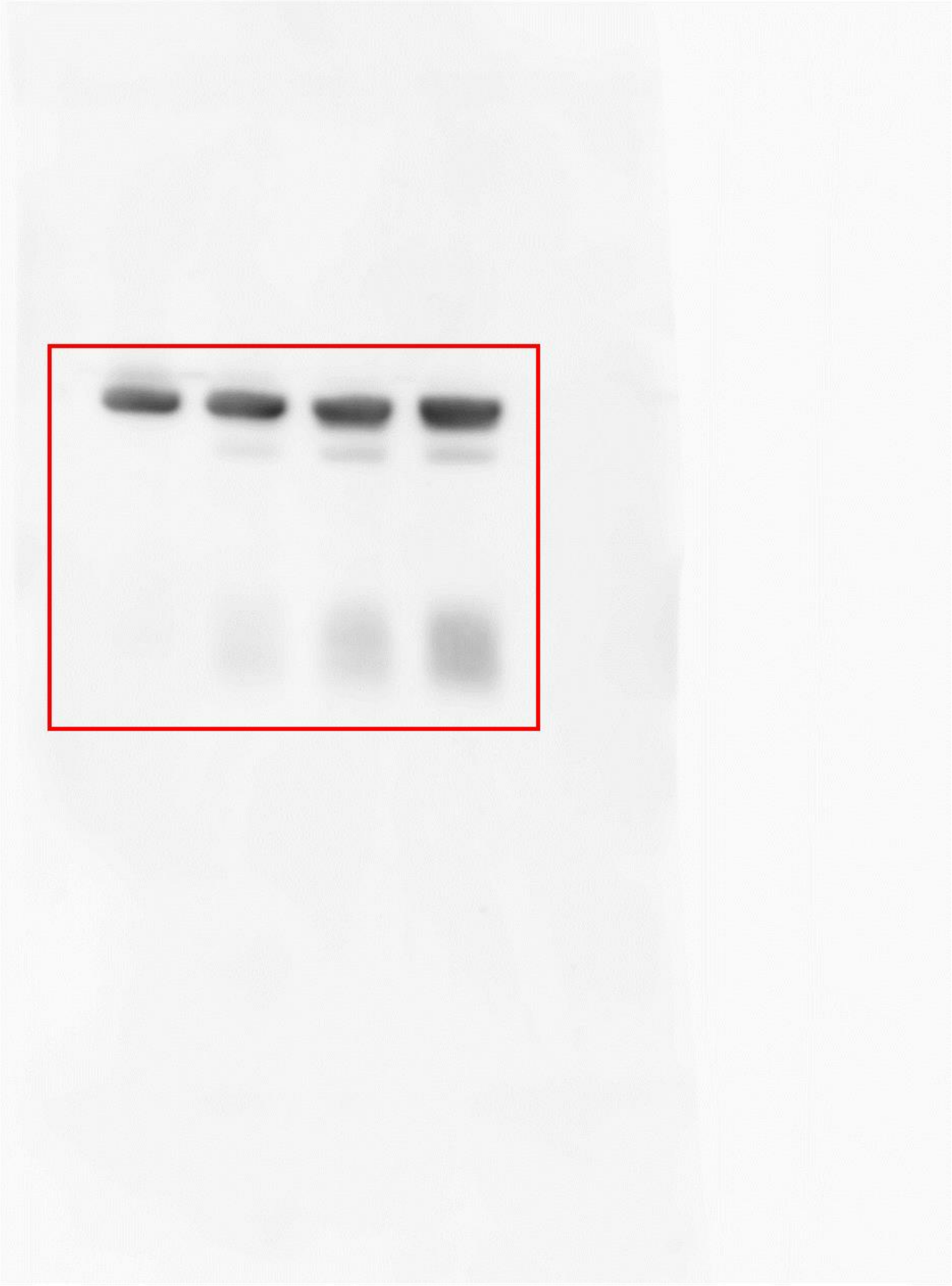

Figure 4C right panel

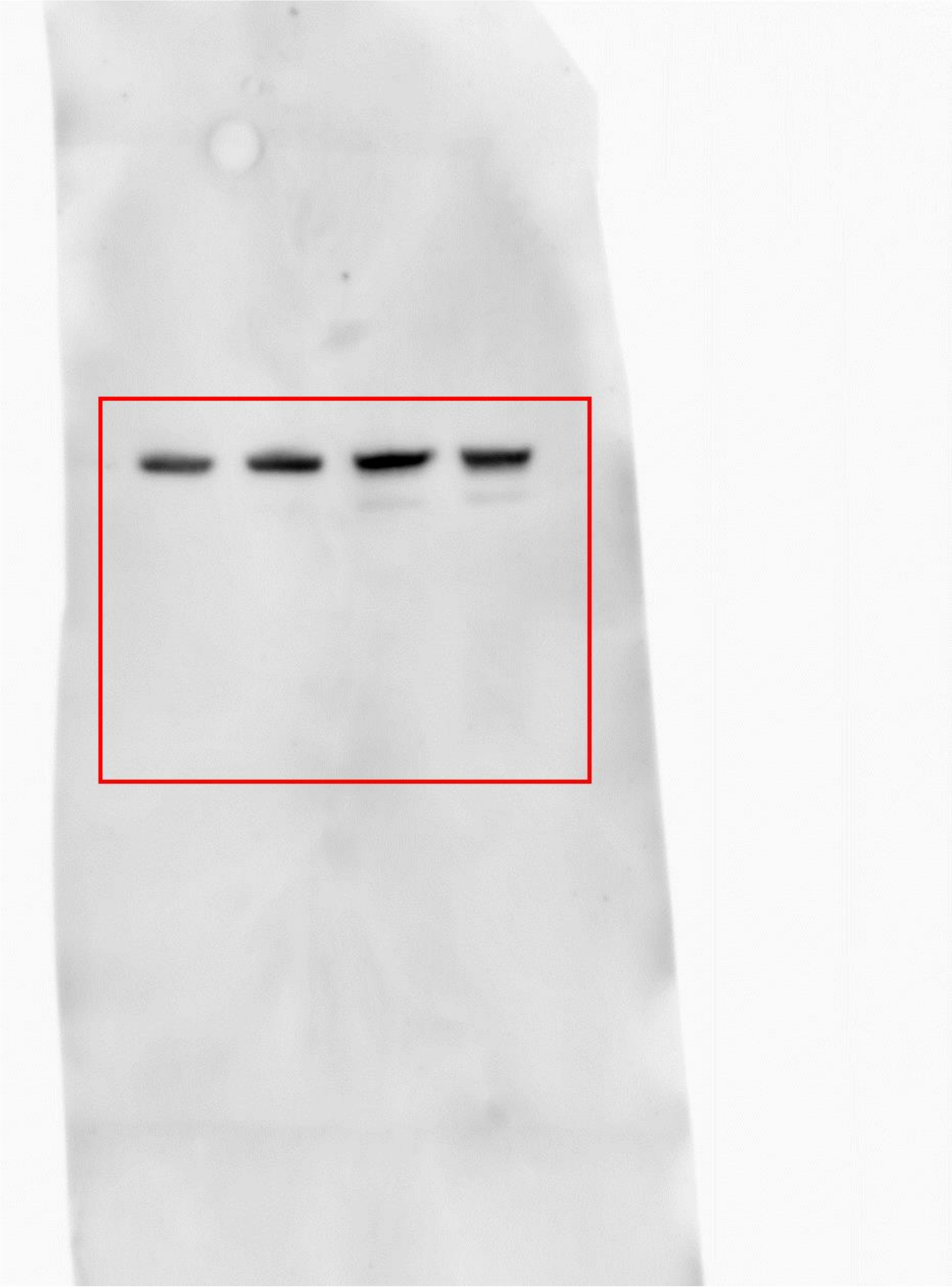

Figure 4D gel before transfer (loading control)

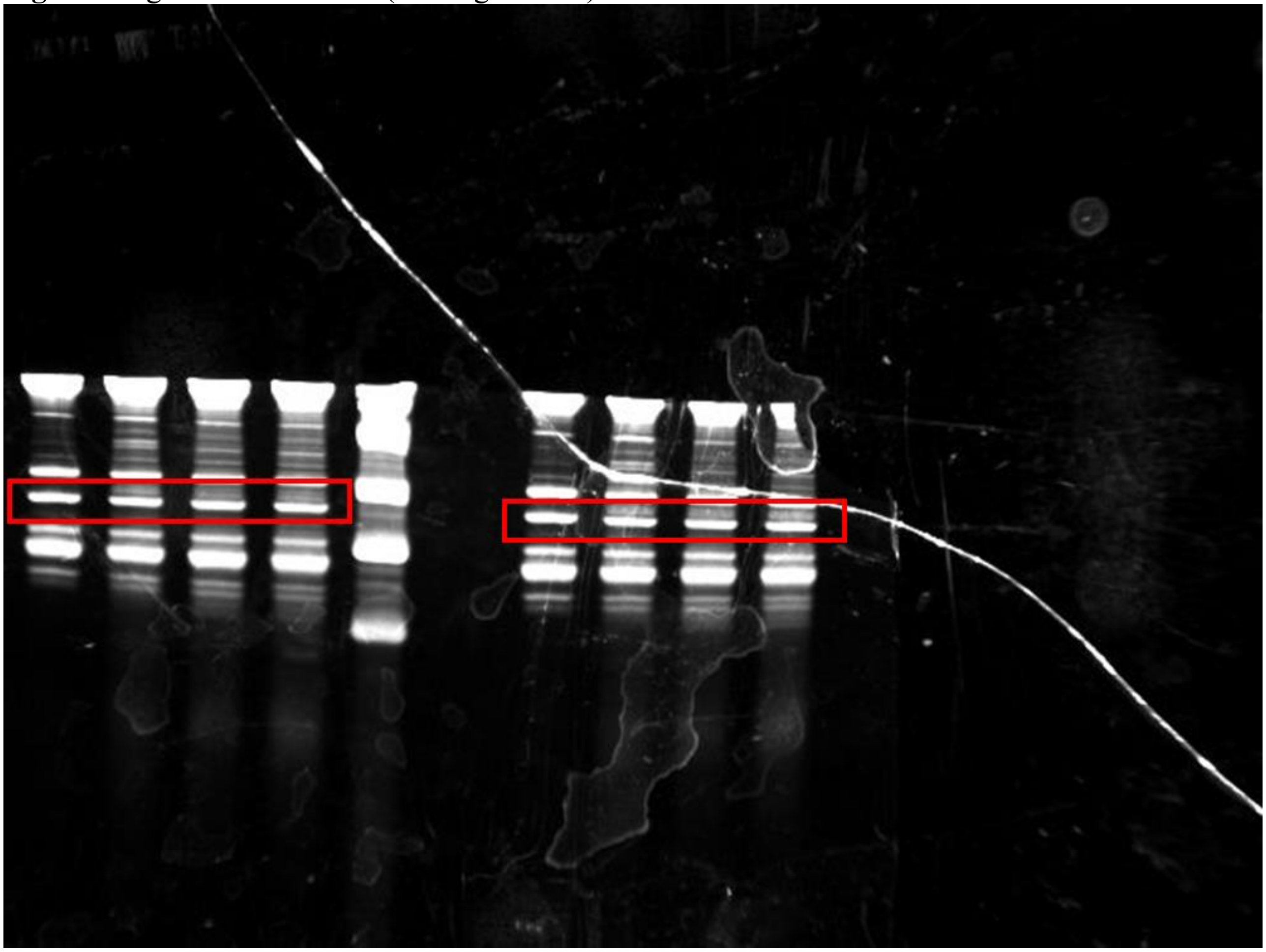

Figure 4D left panel

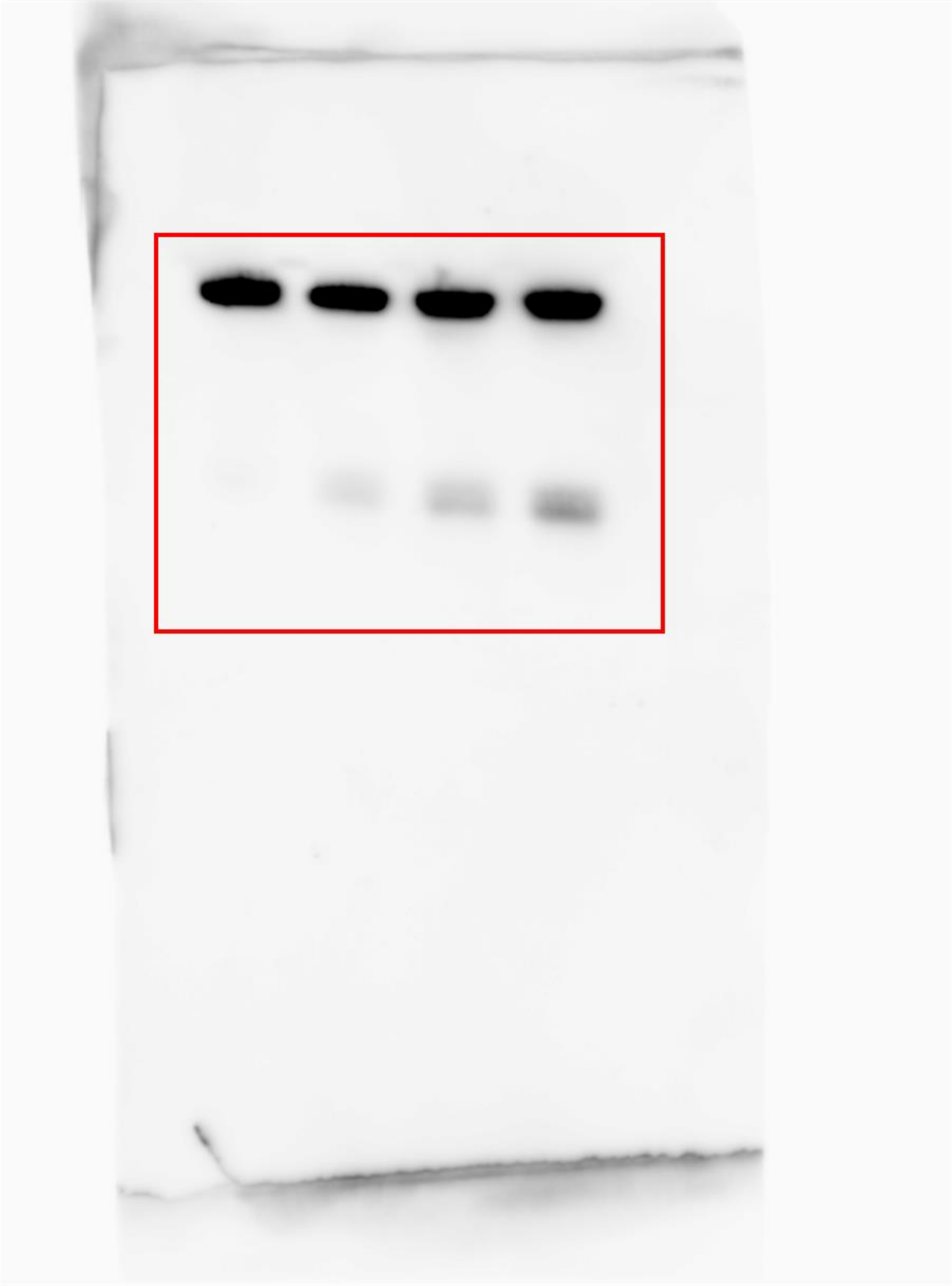

Figure 4D right panel

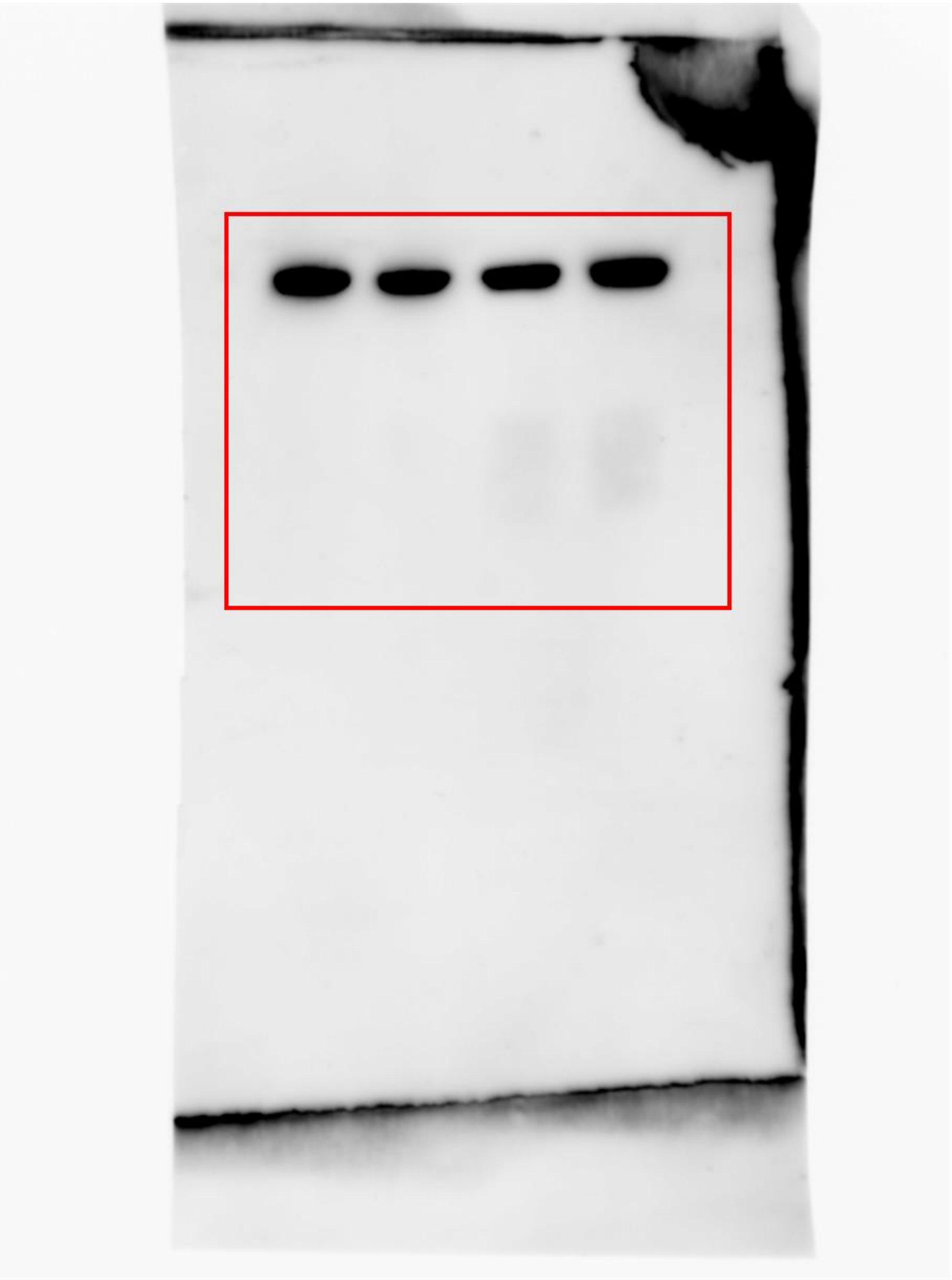

Figure 4E gel before transfer (loading control)

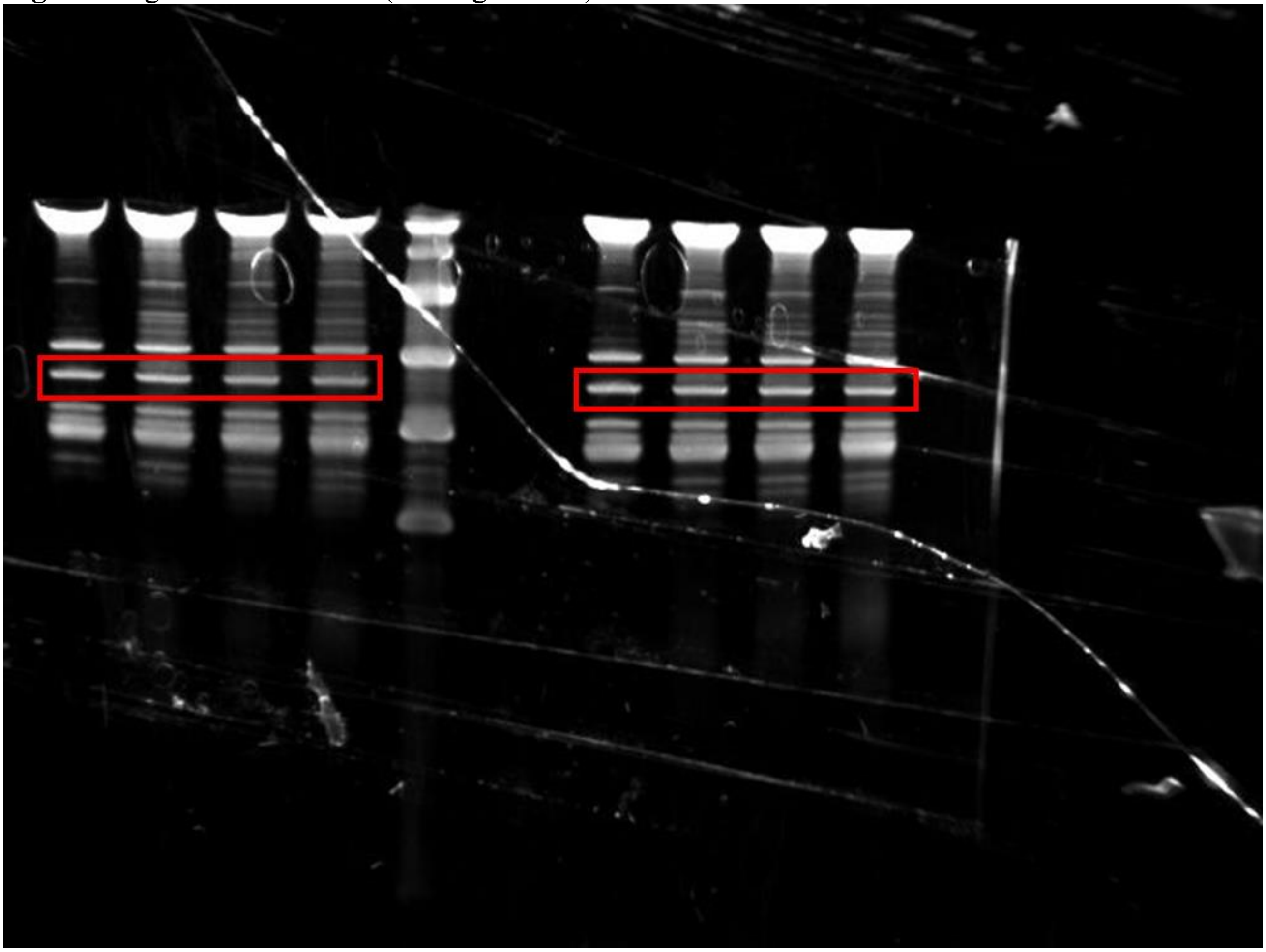

Figure 4E left panel

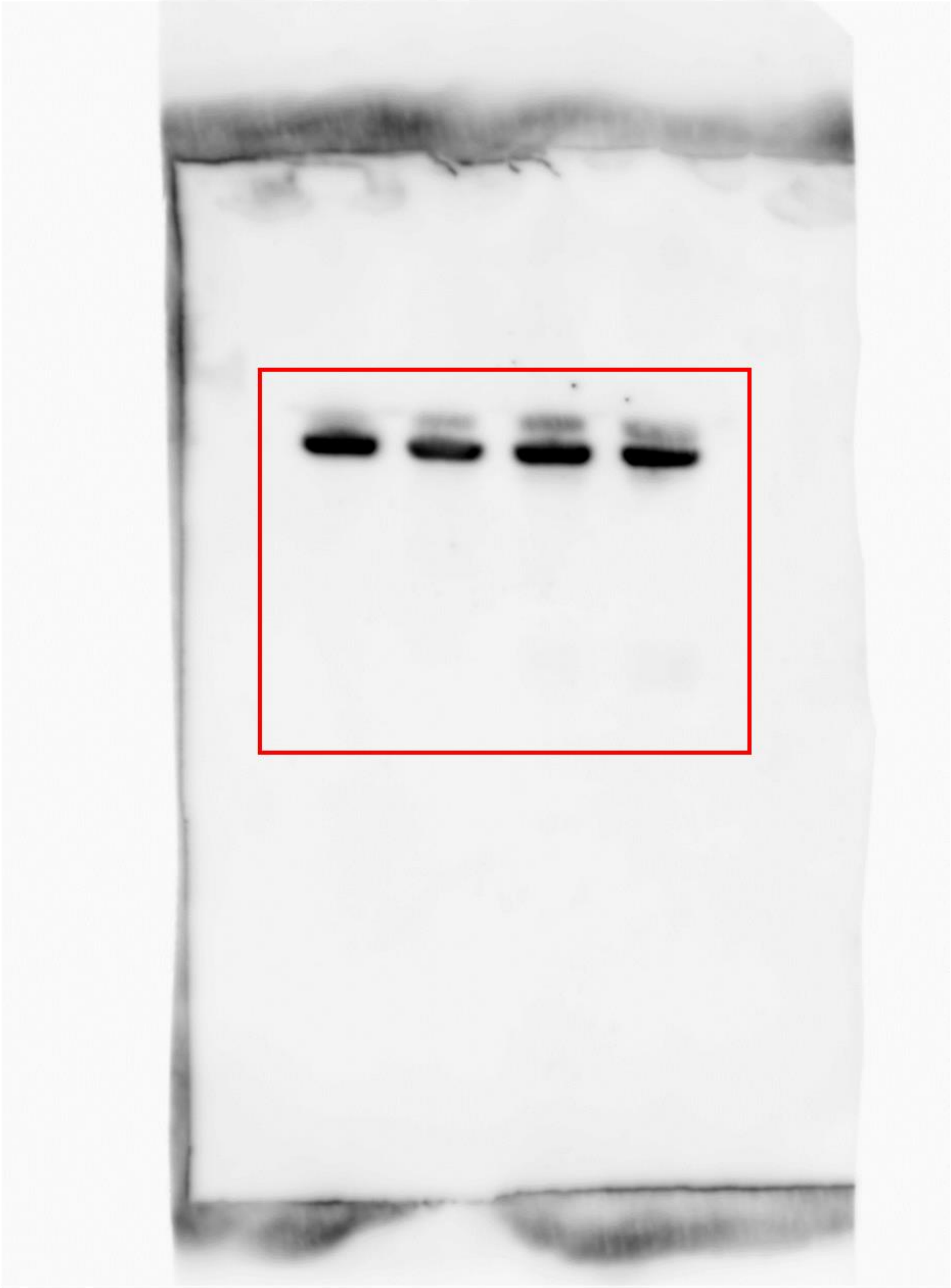

Figure 4E right panel

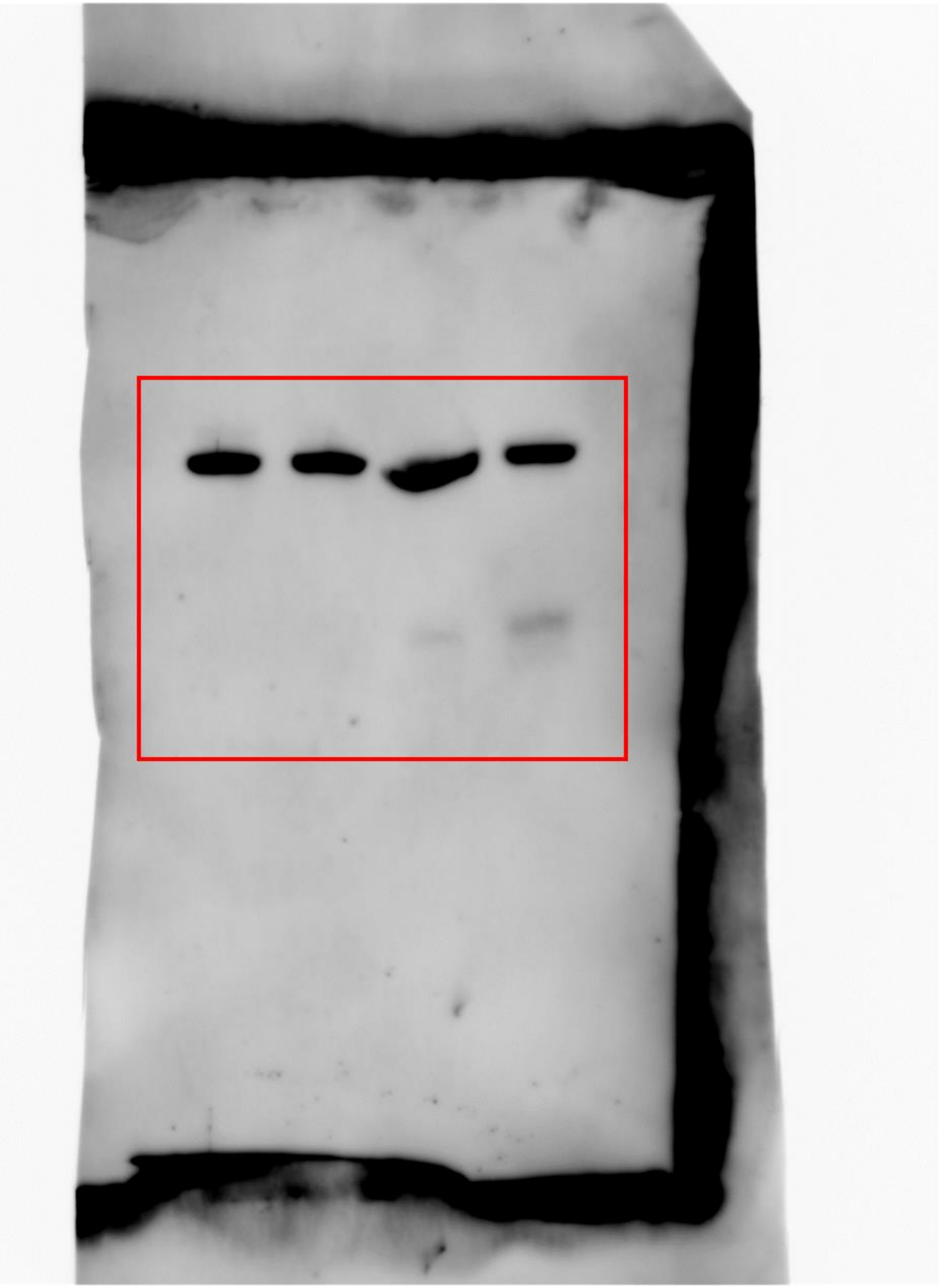

Figure 4F gel before transfer (loading control)

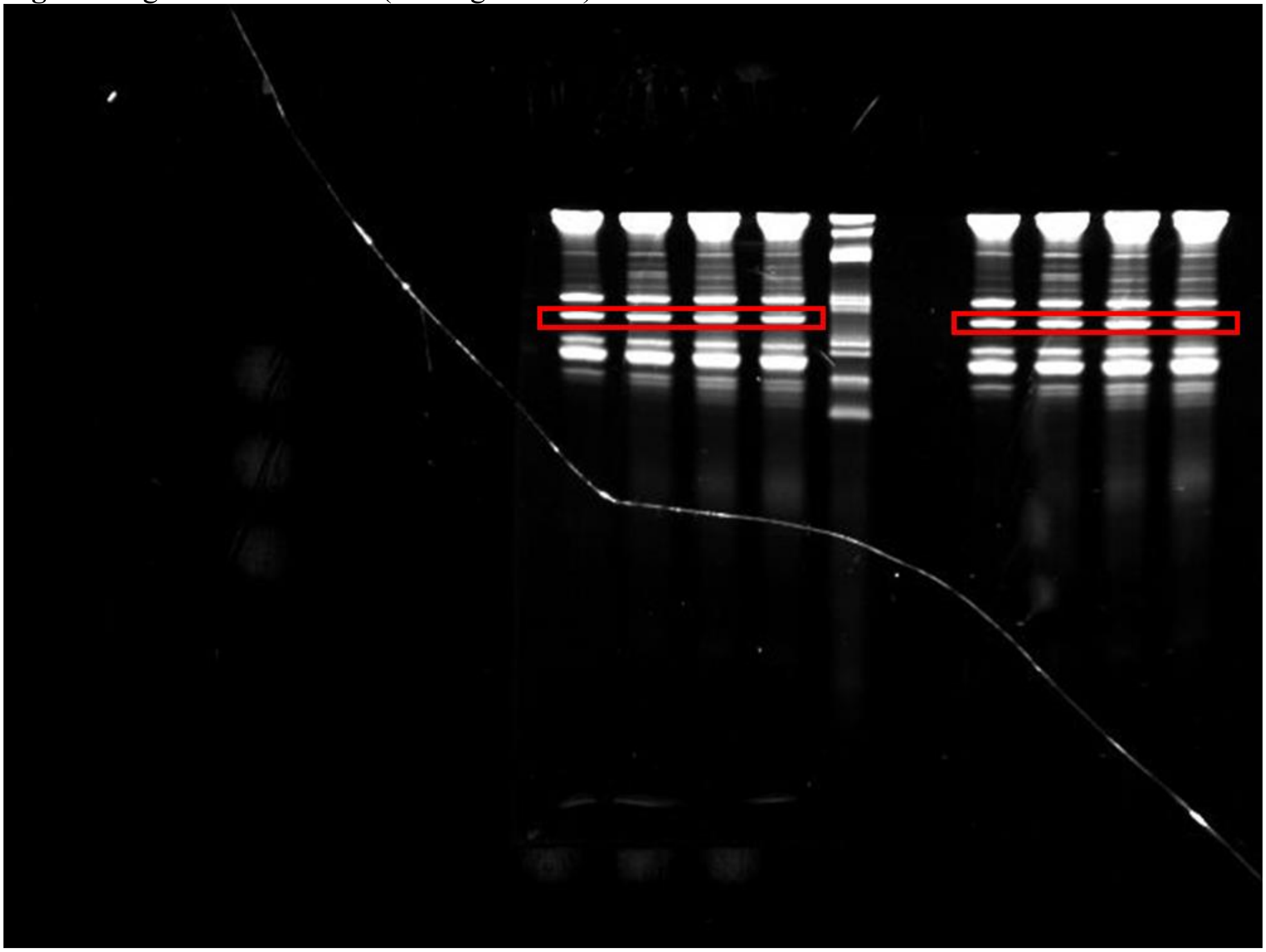

Figure 4F left panel

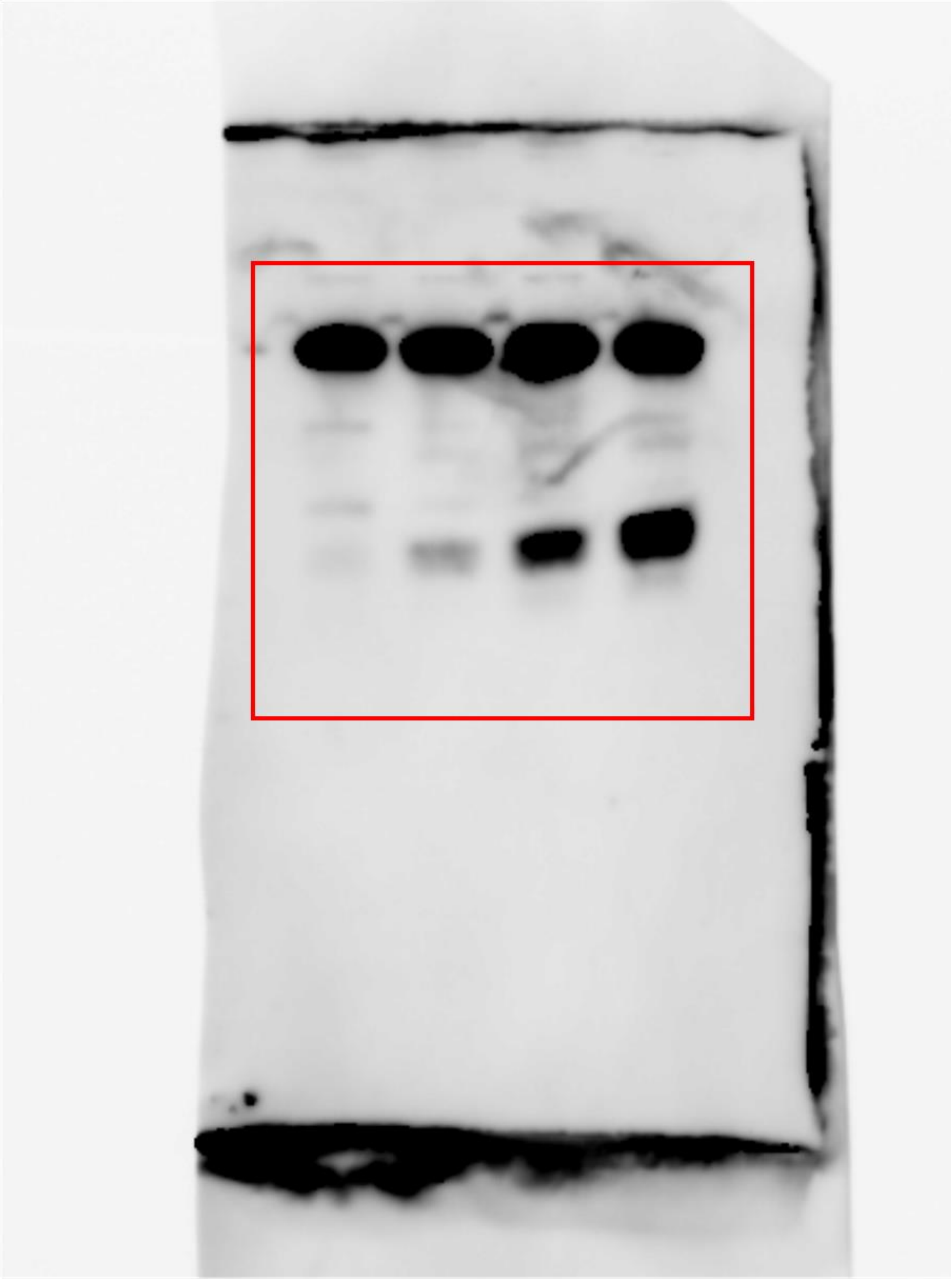

Figure 4F right panel

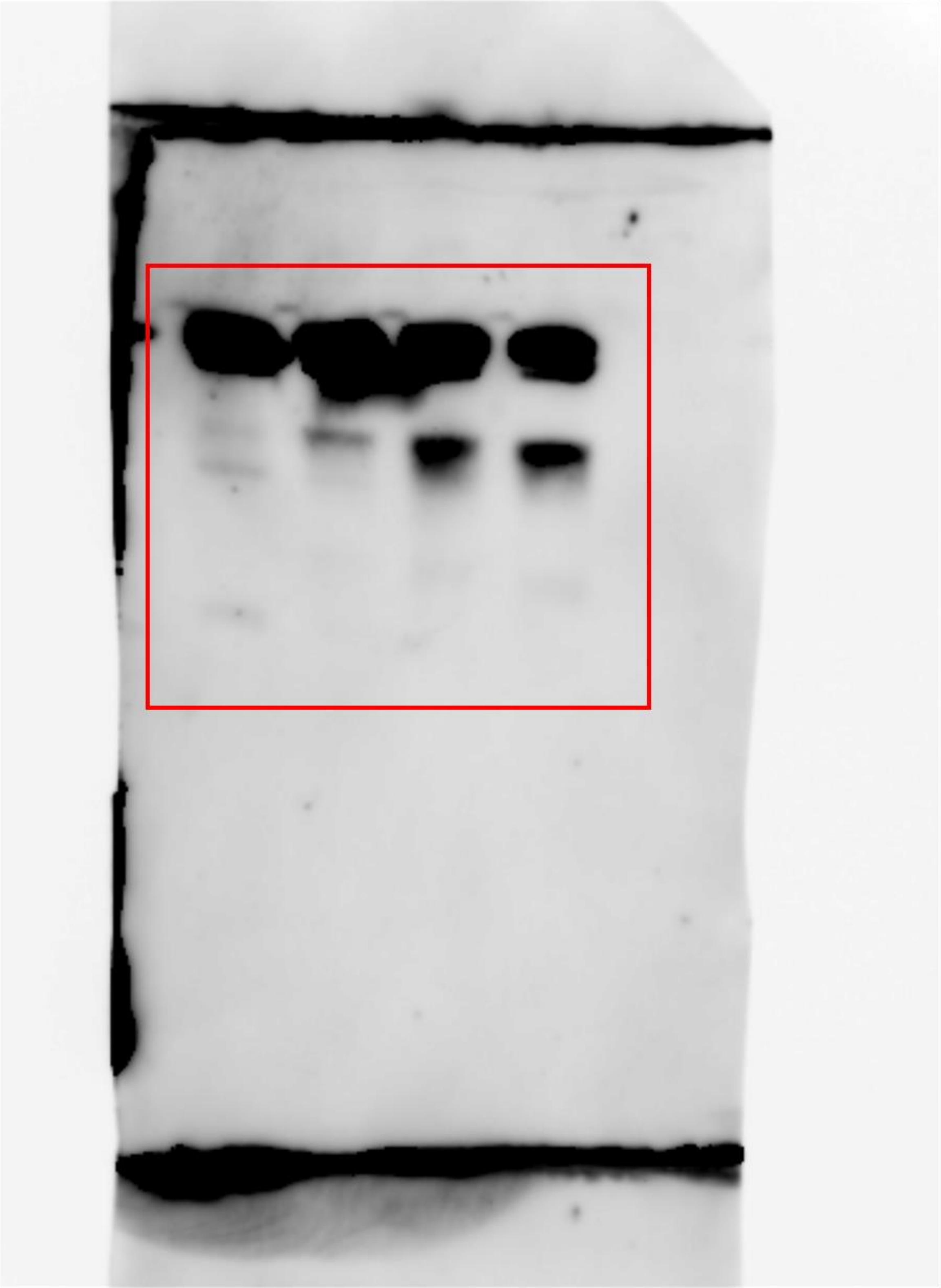

Supplementary Table S1. Count of sequencing reads aligned to tRNA genes

Chr	tRNA	tRNA Bounds		tRNA	Anticodon	Strand	All read alignment				Partial alignment			
#	#	Start	End	Isotype		Direction	Day0	Day5	Day8	Day12	Day0	Day5	Day8	Day12
I	1	185335	185412	Pseudo	CTT	1	1	1	1	0	0	0	1	0
I	2	754683	754769	Leu	CAG	1	102	62	120	226	0	4	2	2
I	3	803803	803878	Pseudo	CTA	1	1	0	1	0	0	0	0	0
I	76	946044	946118	Trp	CCA	-1	59	98	85	69	5	5	8	10
I	4	1447140	1447215	Arg	TCT	1	40	41	37	42	1	1	2	1
I	5	1575722	1575797	Arg	TCT	1	26	34	33	31	1	0	3	0
I	75	2272367	2272440	Gly	GCC	-1	10	14	82	165	1	0	0	1
I	74	3638852	3638927	Met	CAT	-1	12	20	10	18	0	1	0	1
I	73	5843083	5843158	Arg	ACG	-1	20	130	40	28	0	0	0	2
I	72	5854794	5854868	Pro	AGG	-1	11	14	24	42	0	0	0	1
I	71	6051154	6051238	Leu	AAG	-1	70	54	135	199	0	0	0	2
I	6	6135832	6135918	Tyr	GTA	1	33	88	206	373	2	2	7	1
I	7	6136599	6136674	Lys	CTT	1	165	192	203	246	7	0	1	1
I	70	6163470	6163546	Ile	AAT	-1	9	23	34	84	5	8	3	2
I	8	6164193	6164269	Ile	AAT	1	104	514	828	2175	1	6	9	1
I	69	6212820	6212894	Pseudo	AAC	-1	2	2	0	2	1	1	0	0
I	9	6781804	6781878	Cys	GCA	1	33	80	65	65	1	1	0	2
I	68	7279350	7279425	Lys	CTT	-1	11	13	16	29	9	7	2	1
I	67	7718875	7718954	Asn	GTT	-1	3	4	7	9	0	0	0	0
I	10	7842909	7842986	Pseudo	CTT	1	1	0	0	0	0	0	0	0
I	11	7960832	7960919	Ile	TAT	1	5	2	5	5	0	0	0	0
I	66	8530119	8530193	Glu	CTC	-1	4	7	4	14	0	2	2	1
I	65	8781145	8781232	Undet	???	-1	7	25	51	148	4	20	44	135
I	12	8791348	8791423	Pseudo	CTT	1	0	0	0	0	0	0	0	0
I	13	8953162	8953237	Lys	TTT	1	55	121	312	430	0	1	0	0
I	64	9051243	9051318	Asn	GTT	-1	0	9	15	17	0	1	1	0
I	14	9267115	9267190	Arg	TCG	1	202	131	231	318	1	0	1	0
I	63	9268918	9268992	Pro	AGG	-1	12	14	28	45	1	1	0	1
I	62	9319954	9320028	Gly	TCC	-1	2	3	2	2	1	1	0	1
I	15	9320170	9320244	Gly	TCC	1	30	60	69	201	0	0	1	1
I	61	9327413	9327487	Gly	TCC	-1	1	1	3	3	0	1	0	2
I	16	9327629	9327703	Gly	TCC	1	19	29	67	135	0	0	0	0
I	60	9328405	9328479	Gly	TCC	-1	0	1	0	4	0	0	0	1
I	17	9328622	9328696	Gly	TCC	1	23	39	49	113	0	0	0	0
I	18	9558784	9558869	Gln	TTG	1	3	1	1	7	0	0	0	0
I	19	9562345	9562432	Ile	TAT	1	2	0	3	5	0	0	0	0
I	59	9563832	9563919	Ile	TAT	-1	3	2	3	5	0	0	0	0
I	20	9605748	9605832	Ser	TGA	1	19	17	44	67	1	0	2	0
I	21	9679635	9679712	Pseudo	CTT	1	0	0	0	2	0	0	0	1
I	22	9883687	9883761	Glu	TTC	1	111	166	425	1138	0	0	0	0
I	58	10133588	10133661	Pseudo	TGG	-1	2	3	3	0	2	0	0	0
I	23	10593126	10593199	Gly	GCC	1	146	155	228	414	0	0	1	4

I	57	10601119	10601204	Gly	CCC	-1	0	3	1	1	0	0	0	1
I	24	10601840	10601925	Gly	CCC	1	0	2	1	1	0	1	0	0
I	56	10604120	10604205	Gly	CCC	-1	0	1	1	0	0	0	0	0
I	25	10807193	10807267	Asp	GTC	1	330	373	484	918	0	2	1	1
I	26	10871611	10871686	Pseudo	CTT	1	0	1	0	1	0	1	0	0
I	55	10872679	10872754	Pseudo	CTT	-1	0	1	0	0	0	1	0	0
I	54	10927403	10927477	Gly	TCC	-1	2	4	4	3	0	0	1	1
I	27	10927576	10927650	Gly	TCC	1	10	20	37	91	0	2	1	0
I	28	10945814	10945889	Phe	GAA	1	1	4	4	13	1	0	0	0
I	29	11158619	11158692	Gly	GCC	1	29	58	22	38	0	0	1	1
I	30	11505542	11505616	Cys	GCA	1	31	76	66	56	0	0	0	1
I	53	11579694	11579769	Lys	CTT	-1	1	3	1	12	1	3	1	5
I	52	11584110	11584185	Lys	CTT	-1	1	5	4	4	0	1	1	0
I	31	11584778	11584853	Lys	CTT	1	20	71	126	172	0	1	1	2
I	51	11585358	11585442	Leu	AAG	-1	39	51	142	221	0	2	4	1
I	50	11803304	11803388	Ser	CGA	-1	5	9	12	21	0	0	0	0
I	49	11977179	11977253	Glu	CTC	-1	2	2	4	18	0	1	0	2
I	32	11977983	11978057	Glu	CTC	1	340	201	255	526	0	1	1	0
I	33	11978755	11978829	Glu	CTC	1	339	221	273	590	0	0	0	1
I	34	12065856	12065931	Val	AAC	1	68	69	77	116	5	4	2	5
I	35	12142902	12142976	Met	CAT	1	261	152	319	529	0	0	0	2
I	36	12198594	12198669	Arg	TCT	1	22	24	28	31	1	0	1	4
I	48	12225414	12225488	Met	CAT	-1	28	17	12	46	0	1	0	0
I	37	12229300	12229374	Met	CAT	1	289	176	321	472	0	0	0	1
I	38	12601768	12601838	Ala	AGC	1	152	214	472	600	1	0	0	1
I	47	12731645	12731719	Pseudo	AAG	-1	0	0	0	2	0	0	0	2
I	46	13156343	13156418	Val	AAC	-1	23	8	6	9	22	4	2	0
I	39	13161050	13161125	Val	AAC	1	151	153	322	535	6	12	28	51
I	45	13297659	13297734	Lys	CTT	-1	2	2	7	14	1	2	1	0
I	44	13310331	13310405	Thr	TGT	-1	3	7	22	33	1	0	3	1
I	43	13324166	13324240	Gly	TCC	-1	1	3	3	2	0	0	0	0
I	40	13324984	13325058	Gly	TCC	1	53	66	101	180	37	43	41	33
I	42	13331133	13331207	Gly	TCC	-1	0	1	0	0	0	1	0	0
I	41	14162836	14162920	Ser	GCT	1	34	111	200	1063	0	0	2	4
II	80	1088448	1088523	Pseudo	GGT	-1	1	0	1	0	0	0	0	0
II	79	1102057	1102131	Pseudo	TGT	-1	0	0	1	0	0	0	0	0
II	78	1102364	1102439	Thr	TGT	-1	0	0	0	0	0	0	0	0
II	1	1331991	1332065	Pseudo	GGG	1	0	0	0	2	0	0	0	0
II	77	1333255	1333329	Pseudo	GGT	-1	0	1	0	1	0	0	0	0
II	2	1334139	1334213	Pseudo	AGT	1	0	1	0	0	0	0	0	0
II	3	1335745	1335819	Pseudo	GGG	1	0	1	0	1	0	0	0	0
II	76	1366400	1366476	Pseudo	GGT	-1	0	0	1	0	0	0	0	0
II	4	1367948	1368023	Pseudo	CGA	1	0	0	0	0	0	0	0	0
II	75	1476010	1476085	Pseudo	GGT	-1	0	0	0	0	0	0	0	0
II	74	1476160	1476237	Pseudo	GGT	-1	0	0	0	0	0	0	0	0
II	5	1478763	1478838	Pseudo	GGT	1	0	0	0	1	0	0	0	0
II	6	1481925	1482000	Pseudo	ATG	1	0	0	0	0	0	0	0	0
II	7	1520042	1520126	Ser	AGA	1	662	339	271	775	2	1	2	2

II	8	2443056	2443124	Pseudo	???	1	1	1	0	1	0	0	0	0
II	73	2735796	2735880	Leu	AAG	-1	83	47	90	134	1	3	2	5
II	72	2736278	2736362	Leu	AAG	-1	19	36	96	138	0	1	5	2
II	9	2860347	2860431	Leu	AAG	1	215	224	770	1215	1	0	1	6
II	71	2861552	2861636	Leu	AAG	-1	8	25	92	106	0	0	4	0
II	70	3062207	3062280	Gly	GCC	-1	10	25	30	69	1	5	3	4
II	10	3062387	3062460	Gly	GCC	1	175	144	230	481	5	1	2	4
II	11	3267993	3268077	Ser	AGA	1	297	195	219	693	0	4	8	2
II	69	3439219	3439293	Thr	AGT	-1	4	7	9	12	0	0	1	0
II	68	3439394	3439469	Lys	CTT	-1	0	5	12	11	0	3	4	3
II	67	3519086	3519160	Thr	AGT	-1	4	4	11	13	0	0	0	0
II	66	3519250	3519325	Lys	CTT	-1	0	5	11	9	0	3	3	0
II	65	3521487	3521561	His	GTG	-1	0	2	2	6	0	2	0	1
II	64	3567610	3567684	Thr	AGT	-1	2	2	8	14	0	0	0	0
II	63	3567764	3567839	Lys	CTT	-1	1	0	13	7	0	0	0	0
II	62	4565010	4565084	Ala	AGC	-1	10	16	15	26	0	0	0	0
II	12	4565386	4565460	Ala	AGC	1	1374	1362	1056	1324	0	0	0	0
II	13	5032762	5032838	Ile	AAT	1	178	579	902	2307	2	2	5	4
II	61	5057436	5057512	Ile	AAT	-1	15	29	21	32	1	1	2	3
II	14	5237849	5237925	Ile	AAT	1	160	530	859	2121	2	7	12	18
II	15	5292315	5292389	Ala	AGC	1	859	735	668	830	3	2	2	5
II	16	5293435	5293509	Glu	CTC	1	189	145	183	321	0	3	0	1
II	60	5577180	5577255	Val	TAC	-1	141	136	140	130	3	1	3	4
II	59	5772893	5772979	Leu	TAA	-1	44	64	176	360	0	0	0	0
II	58	5782189	5782262	Gly	GCC	-1	14	32	92	238	0	4	2	3
II	57	6358332	6358406	Pseudo	TTC	-1	0	0	0	1	0	0	0	1
II	17	6565216	6565291	Lys	TTT	1	24	87	138	543	0	0	0	0
II	18	6721729	6721814	Leu	TAA	1	126	161	381	939	1	2	1	4
II	19	6853161	6853245	Leu	TAG	1	34	98	194	709	1	0	0	4
II	20	6898150	6898224	Glu	TTC	1	193	343	398	1049	0	2	2	0
II	21	7001585	7001659	Pseudo	TGC	1	122	71	59	135	12	3	1	2
II	56	7002948	7003022	Ala	AGC	-1	8	12	11	24	1	0	0	0
II	22	7003231	7003305	Thr	AGT	1	652	815	783	1027	3	2	2	3
II	55	7756897	7756971	Gln	TTG	-1	1	2	5	15	0	0	0	0
II	54	8631904	8631990	Leu	TAA	-1	22	48	80	303	0	0	0	1
II	53	9211741	9211827	Tyr	GTA	-1	36	47	85	115	3	4	0	0
II	23	9404299	9404376	Pseudo	GTG	1	8	22	8	22	0	0	0	4
II	24	9566182	9566257	Arg	TCG	1	245	178	212	344	0	0	0	0
II	52	9713162	9713237	Phe	GAA	-1	180	476	111	152	0	6	2	7
II	25	9773595	9773672	Lys	CTT	1	0	5	9	12	0	1	1	0
II	51	10302096	10302170	Pro	TGG	-1	3	1	5	9	3	0	0	0
II	26	10302339	10302413	Pro	TGG	1	29	27	33	57	0	0	0	2
II	50	10304708	10304782	Pro	TGG	-1	3	2	4	4	1	0	1	1
II	27	10304951	10305025	Pro	TGG	1	19	31	37	46	0	1	1	0
II	49	11157741	11157816	Lys	CTT	-1	1	5	6	14	1	2	1	0
II	48	11333946	11334020	Ala	TGC	-1	4	9	14	8	0	0	0	0
II	28	11334444	11334518	Ala	TGC	1	83	271	175	212	0	1	1	1
II	29	11441126	11441203	Pseudo	CTT	1	0	0	0	2	0	0	0	2

II	47	11442183	11442260	Pseudo	CTT	-1	0	1	0	0	0	0	0	0
II	30	11959312	11959387	Val	TAC	1	63	68	75	124	5	2	4	3
II	46	12345813	12345886	Pseudo	GCT	-1	0	1	0	0	0	0	0	0
II	45	12349569	12349643	Pseudo	ATG	-1	0	2	0	2	0	1	0	1
II	31	12362385	12362461	Ile	AAT	1	137	108	185	301	1	1	4	3
II	44	12435513	12435587	Met	CAT	-1	7	11	15	30	0	0	1	0
II	32	12494677	12494753	Ile	AAT	1	87	93	199	269	2	6	17	9
II	33	12539533	12539607	Trp	CCA	1	215	373	432	424	1	0	9	2
II	43	12541621	12541696	Lys	TTT	-1	4	6	16	47	0	0	1	1
II	42	12541863	12541947	Leu	AAG	-1	3	4	12	22	0	1	2	1
II	34	12542570	12542644	Ala	AGC	1	705	570	364	530	0	2	1	3
II	41	12666090	12666164	Pseudo	???	-1	0	0	2	0	0	0	0	0
II	40	12669925	12670000	Pseudo	GTG	-1	0	0	0	0	0	0	0	0
II	35	12678245	12678319	Ala	TGC	1	79	262	206	189	1	1	0	1
II	39	12728682	12728756	Arg	CCG	-1	2	4	23	47	0	0	0	0
II	38	14474764	14474838	Gln	TTG	-1	4	2	9	18	0	0	0	0
II	36	14617006	14617078	Pseudo	GTT	1	19	65	182	265	18	65	181	264
II	37	14635257	14635341	Leu	AAG	-1	56	91	283	992	0	0	0	4
III	1	535380	535466	Tyr	GTA	1	35	91	189	354	2	4	14	2
III	2	1040666	1040739	Pseudo	GCC	1	0	1	1	1	0	0	0	0
III	3	1041543	1041616	Pseudo	GCC	1	0	0	1	0	0	0	0	0
III	4	1042576	1042649	Pseudo	GCC	1	0	0	0	0	0	0	0	0
III	5	1044987	1045060	Pseudo	???	1	0	0	1	0	0	0	0	0
III	6	1046932	1047005	Pseudo	TGG	1	0	1	0	0	0	0	0	0
III	7	1047820	1047894	Pseudo	GCC	1	0	0	0	1	0	0	0	0
III	97	1163342	1163418	Ile	AAT	-1	60	41	37	81	0	1	2	3
III	96	1164788	1164864	Ile	AAT	-1	2	14	14	62	0	1	4	2
III	95	1218365	1218441	Ile	AAT	-1	6	36	47	109	1	6	9	2
III	8	1424483	1424557	Gly	TCC	1	87	85	63	138	1	2	3	1
III	94	1575322	1575397	Pseudo	TTG	-1	0	0	0	3	0	0	0	0
III	9	1575422	1575497	Pseudo	TCG	1	0	0	0	0	0	0	0	0
III	93	1575764	1575842	Pseudo	TTG	-1	0	0	0	0	0	0	0	0
III	10	1575864	1575939	Pseudo	TCG	1	1	7	1	2	1	7	0	1
III	92	1576997	1577070	Pseudo	TTG	-1	0	0	0	0	0	0	0	0
III	11	1577093	1577168	Pseudo	CGA	1	1	0	0	0	0	0	0	0
III	91	1577307	1577382	Pseudo	CGA	-1	0	0	1	1	0	0	0	0
III	12	1577403	1577477	Pseudo	CGA	1	0	0	0	2	0	0	0	0
III	13	1604573	1604647	Met	CAT	1	207	128	261	405	0	0	1	0
III	90	1604690	1604764	Glu	CTC	-1	6	5	4	16	0	0	0	1
III	89	1743237	1743311	Pseudo	TCG	-1	0	2	1	11	0	0	1	4
III	14	1998074	1998150	Pseudo	GCG	1	1	1	0	0	0	0	0	0
III	15	2032508	2032582	Glu	CTC	1	363	249	189	392	0	1	1	1
III	88	2032884	2032958	Glu	CTC	-1	86	104	34	26	1	1	0	1
III	16	2711703	2711777	Pro	TGG	1	34	106	139	112	3	1	0	1
III	17	2879907	2879983	Pseudo	ATG	1	0	0	0	1	0	0	0	0
III	18	3099566	3099640	Pseudo	AAG	1	0	1	0	1	0	0	0	0
III	19	3101077	3101151	Pseudo	GAG	1	2	1	0	3	0	0	0	0
III	20	3103005	3103080	Pseudo	GAG	1	2	6	15	27	0	0	0	0

III	87	3104051	3104125	Pseudo	GAG	-1	1	0	0	0	0	0	0	0
III	21	3105134	3105209	Pseudo	GGG	1	1	0	0	0	1	0	0	0
III	86	3106136	3106210	Pseudo	GAG	-1	0	0	0	0	0	0	0	0
III	85	3108250	3108325	Pseudo	GAG	-1	0	0	0	0	0	0	0	0
III	22	3110790	3110865	Pseudo	GGG	1	0	0	0	0	0	0	0	0
III	84	3112133	3112207	Pseudo	GAG	-1	1	0	0	0	1	0	0	0
III	83	3186978	3187052	Pseudo	GAG	-1	0	0	0	0	0	0	0	0
III	23	3189660	3189736	Pseudo	GAG	1	0	0	0	1	0	0	0	0
III	24	3191642	3191714	Pseudo	GTC	1	0	0	0	0	0	0	0	0
III	25	3195430	3195514	Tyr	GTA	1	46	87	205	389	1	1	0	1
III	82	3339462	3339536	Pseudo	ACG	-1	0	0	0	1	0	0	0	0
III	26	3389527	3389602	Lys	CTT	1	55	187	655	747	1	1	1	0
III	81	3426900	3426975	Lys	CTT	-1	0	1	2	1	0	0	0	0
III	27	4362496	4362618	Leu	CAA	1	63	144	276	847	0	1	0	3
III	80	4428922	4428996	Ala	AGC	-1	8	6	8	15	1	0	0	0
III	79	4449499	4449574	Arg	ACG	-1	13	70	31	33	0	0	0	1
III	78	4450290	4450365	Arg	ACG	-1	16	42	29	16	0	0	0	1
III	77	4470852	4470927	Arg	TCG	-1	82	56	85	191	0	1	1	2
III	76	5294937	5295011	Met	CAT	-1	11	5	9	21	1	0	0	0
III	28	5305866	5305940	Thr	TGT	1	10	20	71	92	0	1	0	1
III	75	5762007	5762079	Gly	GCC	-1	8	4	16	51	1	0	1	0
III	29	5762239	5762312	Gly	GCC	1	167	164	213	400	2	3	4	5
III	30	5769859	5769943	Leu	AAG	1	63	149	377	967	1	3	8	7
III	74	5782188	5782261	Gly	GCC	-1	2	6	7	10	0	0	3	3
III	73	6056245	6056329	Ser	GCT	-1	1	12	29	88	0	0	0	0
III	31	6158726	6158810	Ser	AGA	1	486	238	210	258	2	3	4	3
III	32	6158913	6159001	Ser	CGA	1	26	20	54	97	0	3	12	32
III	72	6513881	6513965	Ser	CGA	-1	9	3	7	6	0	0	0	0
III	71	6724077	6724165	Lys	CTT	-1	0	3	3	7	0	0	0	0
III	70	6726933	6727021	Arg	CCT	-1	0	2	2	6	0	0	0	0
III	33	6805513	6805588	Asn	GTT	1	60	66	90	154	0	0	3	4
III	69	6887671	6887746	Lys	TTT	-1	2	8	15	30	0	1	1	3
III	68	6888553	6888627	Gly	TCC	-1	2	1	0	2	1	0	0	0
III	34	6888752	6888826	Gly	TCC	1	29	38	54	111	0	2	3	2
III	67	7030497	7030571	Thr	AGT	-1	3	4	3	10	0	1	0	1
III	66	7642090	7642164	Glu	CTC	-1	18	11	13	26	1	1	1	1
III	65	7978403	7978478	Phe	GAA	-1	3	22	26	8	0	1	1	0
III	35	8069340	8069414	Pro	TGG	1	32	15	25	43	5	0	3	2
III	36	8106587	8106662	Met	CAT	1	185	251	100	126	0	0	0	0
III	37	8109537	8109661	Leu	CAA	1	58	193	295	821	0	0	6	10
III	38	8473396	8473471	Lys	CTT	1	19	96	167	255	0	5	3	1
III	39	8639226	8639348	Leu	CAA	1	124	248	322	909	1	2	4	12
III	40	8646087	8646171	Ser	AGA	1	106	65	192	218	0	0	0	0
III	64	8650298	8650372	Pro	CGG	-1	259	679	1841	2216	1	3	4	4
III	41	8652974	8653049	Phe	GAA	1	7	37	71	42	0	1	1	0
III	63	8653524	8653599	Phe	GAA	-1	1	2	4	5	0	1	0	1
III	62	8678413	8678487	Trp	CCA	-1	31	93	121	136	1	5	3	7
III	61	9104127	9104202	Asn	GTT	-1	1	5	6	15	1	2	6	3

III	60	9861517	9861601	Ser	CGA	-1	6	6	7	12	0	0	0	0
III	59	9861932	9862016	Ser	AGA	-1	36	19	28	30	0	0	0	0
III	58	10624640	10624714	Thr	AGT	-1	3	4	7	10	0	0	0	0
III	57	10961748	10961818	Arg	GCG	-1	0	0	0	0	0	0	0	0
III	56	11066746	11066820	Pro	TGG	-1	3	9	3	5	1	1	1	2
III	42	11066928	11067002	Pro	TGG	1	38	110	141	121	0	0	0	0
III	43	11352929	11353004	Val	AAC	1	42	69	80	97	8	7	3	3
III	44	11353528	11353603	Val	AAC	1	212	183	336	513	46	42	22	17
III	45	11474362	11474437	Pseudo	TCT	1	0	1	0	2	0	0	0	1
III	46	11554725	11554800	Phe	GAA	1	7	39	59	40	1	0	1	0
III	47	11792682	11792756	Thr	CGT	1	16	25	31	87	1	1	1	0
III	55	12399805	12399879	Gln	TTG	-1	1	0	6	9	0	0	1	0
III	48	13009322	13009401	Ser	GCT	1	31	109	198	1002	0	0	0	2
III	54	13016003	13016077	Thr	CGT	-1	9	17	27	50	0	0	0	0
III	49	13224465	13224551	Tyr	GTA	1	30	114	205	394	0	2	2	2
III	53	13225058	13225133	Asn	GTT	-1	0	5	7	9	0	2	0	0
III	52	13411262	13411336	Thr	TGT	-1	101	281	120	125	0	1	2	5
III	50	13522178	13522255	Pseudo	CTT	1	0	0	1	0	0	0	0	0
III	51	13523236	13523313	Pseudo	CTT	-1	0	0	0	0	0	0	0	0
IV	1	66982	67055	Met	CAT	1	272	145	274	451	0	0	0	0
IV	2	143982	144057	Arg	CCT	1	49	121	134	228	1	7	2	4
IV	93	322629	322703	Asp	GTC	-1	8	12	16	22	3	2	3	0
IV	3	620482	620557	Val	CAC	1	107	142	147	150	2	1	1	6
IV	4	658181	658269	SeC(e)	TCA	1	6	21	52	110	1	1	0	2
IV	5	683063	683137	Asp	GTC	1	442	538	785	1319	1	1	1	1
IV	6	842809	842883	Glu	TTC	1	124	104	204	448	0	0	3	0
IV	92	1144681	1144756	Pseudo	GGC	-1	0	0	1	0	0	0	1	0
IV	91	1146071	1146145	Pseudo	ATG	-1	0	0	0	0	0	0	0	0
IV	90	1355410	1355485	Pseudo	???	-1	5	50	38	37	0	0	0	1
IV	7	1365190	1365264	Pseudo	TCG	1	0	0	0	1	0	0	0	0
IV	89	1385619	1385694	Pseudo	???	-1	2	3	2	1	0	1	0	0
IV	88	1388212	1388286	Glu	TTC	-1	4	5	10	38	0	1	2	2
IV	8	1407264	1407337	Pseudo	ATA	1	0	0	0	1	0	0	0	0
IV	9	1464680	1464755	Pseudo	???	1	0	2	0	0	0	0	0	0
IV	87	1474648	1474722	Pseudo	GTG	-1	9	5	18	4	0	0	1	0
IV	10	1514591	1514665	Asp	GTC	1	173	240	439	669	5	2	1	3
IV	86	2639804	2639878	Glu	TTC	-1	4	6	17	34	0	0	0	0
IV	85	2659008	2659083	Pseudo	TTG	-1	1	3	0	2	0	0	0	0
IV	11	2664240	2664315	Pseudo	TCC	1	24	21	12	17	23	18	10	16
IV	12	2740212	2740287	Pseudo	???	1	0	1	1	0	0	0	0	0
IV	13	2800097	2800171	Asp	GTC	1	49	128	71	197	3	2	1	0
IV	84	2801146	2801220	Asp	GTC	-1	21	8	11	11	0	1	3	2
IV	14	2802335	2802409	Asp	GTC	1	350	354	490	976	4	2	3	1
IV	15	2805625	2805699	Asp	GTC	1	19	29	62	129	12	3	11	17
IV	16	2808353	2808427	Asp	GTC	1	47	108	91	205	0	0	2	0
IV	83	3501641	3501718	Pseudo	CTT	-1	0	0	0	1	0	0	0	1
IV	17	3524815	3524890	Arg	ACG	1	106	264	131	97	0	1	1	4
IV	82	3552182	3552256	Pro	CGG	-1	242	699	1798	2256	0	1	6	4

IV	18	3589258	3589333	Pseudo	CTT	1	0	0	1	1	0	0	0	0
IV	81	3590283	3590358	Pseudo	CTT	-1	0	0	0	0	0	0	0	0
IV	80	3681872	3681946	Trp	CCA	-1	121	216	187	200	0	4	4	10
IV	79	4106564	4106639	Pseudo	GGG	-1	1	1	1	4	0	0	0	0
IV	19	4861746	4861818	His	GTG	1	11	26	37	81	1	0	0	0
IV	20	5038912	5038987	Lys	TTT	1	46	111	228	567	0	0	0	0
IV	21	5334704	5334778	Gln	CTG	1	157	253	605	1765	0	1	0	0
IV	78	5344289	5344363	Trp	CCA	-1	26	67	119	97	1	0	0	0
IV	22	5349341	5349416	Pseudo	CTT	1	0	0	0	0	0	0	0	0
IV	23	5967148	5967223	Lys	CTT	1	46	211	582	715	0	1	0	1
IV	24	6481150	6481225	Val	CAC	1	117	146	132	177	0	0	0	1
IV	25	6556807	6556893	Leu	CAG	1	58	56	87	118	9	6	3	7
IV	26	6562191	6562265	Ala	AGC	1	130	212	278	403	0	1	0	3
IV	27	7275316	7275390	Gln	TTG	1	49	67	180	505	0	0	0	1
IV	77	7344142	7344216	Trp	CCA	-1	4	18	23	24	1	3	9	4
IV	28	8927187	8927263	Pseudo	GTC	1	6	41	211	269	6	40	207	266
IV	76	9000786	9000860	Thr	TGT	-1	1	7	14	7	0	0	1	0
IV	75	10311206	10311280	Gln	TTG	-1	1	2	11	17	0	0	0	0
IV	29	11231543	11231617	Pro	AGG	1	20	20	32	41	0	0	0	0
IV	30	11362103	11362178	Pseudo	CTT	1	0	0	3	1	0	0	1	1
IV	74	11363169	11363244	Pseudo	CTT	-1	0	0	0	0	0	0	0	0
IV	31	11721126	11721216	His	GTG	1	0	2	0	9	0	0	0	0
IV	73	11928452	11928573	Leu	CAA	-1	13	16	35	79	0	0	2	2
IV	72	12005572	12005646	Pro	CGG	-1	249	770	1889	2358	0	3	7	12
IV	71	12416916	12416990	Gln	CTG	-1	25	44	46	104	0	0	0	0
IV	70	12417223	12417297	Gln	TTG	-1	6	1	5	14	1	0	0	0
IV	69	12417900	12417975	Arg	ACG	-1	15	28	24	14	0	0	0	0
IV	32	12594716	12594791	Lys	CTT	1	19	63	105	173	0	1	2	2
IV	33	13043668	13043743	Pseudo	CTT	1	0	1	1	2	0	0	0	0
IV	68	13044433	13044508	Pseudo	CTT	-1	0	1	0	0	0	0	0	0
IV	34	13870240	13870314	His	GTG	1	0	1	5	12	0	0	1	0
IV	67	14013777	14013851	Ala	TGC	-1	93	33	14	14	2	0	1	1
IV	35	14225077	14225151	Pro	TGG	1	5	29	30	44	0	0	1	0
IV	66	14576591	14576665	Gly	TCC	-1	2	1	4	2	1	0	2	0
IV	65	14611276	14611351	Arg	ACG	-1	14	16	15	20	2	0	1	4
IV	64	14888987	14889061	His	GTG	-1	19	15	24	41	1	6	0	4
IV	36	15187312	15187386	His	GTG	1	4	14	8	31	1	6	1	0
IV	63	15187619	15187693	His	GTG	-1	8	15	24	39	1	2	1	2
IV	37	15235356	15235431	Val	AAC	1	134	110	168	289	2	1	11	26
IV	38	15289671	15289745	Trp	CCA	1	190	407	496	418	0	4	10	13
IV	62	15311083	15311167	Ser	AGA	-1	13	7	6	8	1	1	1	1
IV	61	15318443	15318527	Ser	AGA	-1	3	0	3	6	1	0	2	1
IV	60	15604277	15604351	Ala	TGC	-1	63	19	9	13	0	0	0	0
IV	39	15793383	15793458	Lys	CTT	1	52	184	587	634	1	2	1	1
IV	59	15931307	15931381	Asp	GTC	-1	18	18	21	29	5	1	1	0
IV	40	15931978	15932052	Asp	GTC	1	151	261	388	633	1	2	2	3
IV	58	15933126	15933200	Glu	TTC	-1	4	6	16	31	0	0	0	0
IV	41	15933321	15933395	Glu	TTC	1	58	99	352	978	0	0	2	2

IV	57	16382396	16382471	Val	AAC	-1	49	15	16	11	45	5	5	3
IV	42	16388424	16388498	His	GTG	1	21	49	75	177	1	15	17	42
IV	56	16389176	16389250	Ala	AGC	-1	25	23	24	26	0	0	0	0
IV	43	16398352	16398426	Ala	AGC	1	850	1060	1187	1526	2	2	1	3
IV	44	16399152	16399226	Ala	AGC	1	812	906	935	1229	0	1	0	6
IV	45	16400098	16400173	Val	AAC	1	41	59	89	111	18	20	17	43
IV	46	16400864	16400938	His	GTG	1	25	57	62	162	9	18	15	31
IV	47	16552187	16552262	Pseudo	TTT	1	1	0	0	0	0	0	0	0
IV	55	16552807	16552882	Pseudo	TTT	-1	1	1	0	0	0	0	0	0
IV	48	16577273	16577357	Ser	TGA	1	15	42	75	120	0	1	1	0
IV	54	16583706	16583790	Ser	TGA	-1	6	22	59	102	0	0	0	0
IV	53	16670830	16670904	Gly	TCC	-1	1	3	4	5	1	1	1	1
IV	49	16681974	16682048	Gly	TCC	1	29	21	15	60	1	0	0	1
IV	52	16684347	16684421	Gly	TCC	-1	4	4	2	9	1	0	0	0
IV	50	16775149	16775223	Gly	TCC	1	78	70	59	151	1	0	1	1
IV	51	16776055	16776129	Gly	TCC	1	24	26	41	109	3	2	0	3
V	1	857326	857400	Ala	AGC	1	131	247	411	578	0	0	0	0
V	170	2149173	2149248	Pseudo	???	-1	2	0	0	2	0	0	0	2
V	169	2664662	2664736	Thr	AGT	-1	3	4	7	14	0	1	3	5
V	2	2674008	2674082	Thr	AGT	1	205	393	361	627	1	0	1	5
V	168	3455320	3455395	Met	CAT	-1	17	16	19	17	0	0	0	0
V	167	3582125	3582199	Pro	TGG	-1	1	1	2	1	1	0	1	0
V	3	3582317	3582391	Pro	TGG	1	33	71	156	126	2	0	0	0
V	166	3588298	3588372	Pro	TGG	-1	2	5	10	7	0	0	3	1
V	4	3588490	3588564	Pro	TGG	1	9	21	27	68	0	0	2	2
V	5	4310596	4310669	Gly	GCC	1	163	253	143	289	0	5	3	5
V	165	4782212	4782287	Pseudo	CTT	-1	0	0	0	0	0	0	0	0
V	164	5330120	5330194	Gln	TTG	-1	0	3	5	8	0	1	0	0
V	6	6335381	6335455	Cys	GCA	1	27	23	38	45	3	0	1	1
V	7	6417958	6418033	Pseudo	CTT	1	0	1	0	1	0	0	0	0
V	163	6421706	6421781	Pseudo	CTT	-1	0	0	0	0	0	0	0	0
V	162	6551834	6551918	Ser	GCT	-1	2	6	7	38	1	0	0	0
V	8	6559740	6559814	Cys	GCA	1	10	13	46	47	0	0	2	1
V	9	6917448	6917535	Ile	TAT	1	37	230	528	1333	2	9	8	12
V	161	7069727	7069800	Glu	TTC	-1	4	6	10	25	0	0	0	0
V	10	7506376	7506451	Arg	TCT	1	31	42	26	31	0	0	0	2
V	160	7730632	7730706	Thr	CGT	-1	22	87	109	135	2	0	0	0
V	159	8230183	8230258	Met	CAT	-1	6	13	12	9	0	0	1	0
V	158	8235435	8235519	Leu	TAG	-1	36	83	202	726	0	1	2	5
V	11	8247461	8247547	Tyr	GTA	1	23	43	82	113	4	1	3	1
V	12	8495281	8495356	Lys	TTT	1	59	107	227	540	2	0	0	0
V	157	8496080	8496154	Thr	AGT	-1	2	0	2	7	0	0	0	0
V	156	8499540	8499615	Lys	TTT	-1	2	6	12	25	0	2	2	0
V	13	9318178	9318252	Ala	CGC	1	82	271	237	267	0	0	0	2
V	155	9407013	9407087	Thr	AGT	-1	8	5	7	16	3	0	2	2
V	154	9412178	9412267	Tyr	GTA	-1	12	16	31	73	0	0	1	0
V	14	9503538	9503612	Ala	CGC	1	77	285	266	261	0	1	2	3
V	153	11174769	11174855	Leu	CAG	-1	120	60	145	212	1	1	6	2

V	152	11183645	11183747	Leu	CAG	-1	18	3	9	19	0	0	0	0
V	151	11412214	11412289	Val	CAC	-1	126	151	127	158	0	0	5	6
V	15	12002157	12002244	Ile	TAT	1	39	226	523	1363	3	6	18	25
V	150	12331943	12332018	Asn	GTT	-1	0	1	4	11	0	0	1	3
V	149	12683331	12683406	Val	TAC	-1	105	125	110	138	0	0	2	5
V	148	12780366	12780450	Ser	TGA	-1	12	17	37	39	0	1	1	1
V	147	13003491	13003565	Ala	AGC	-1	29	32	30	34	1	3	3	5
V	146	13027010	13027085	Lys	TTT	-1	1	10	15	29	1	0	0	0
V	145	13507187	13507261	Pro	TGG	-1	1	5	7	1	1	1	1	1
V	16	13509680	13509754	Pro	TGG	1	5	22	33	59	1	1	1	1
V	17	14231349	14231423	Gln	CTG	1	58	96	138	256	0	0	0	0
V	18	14689517	14689603	Tyr	GTA	1	25	59	155	158	1	3	2	1
V	144	14855769	14855843	Cys	GCA	-1	5	12	22	22	0	0	0	0
V	143	14876371	14876446	Phe	GAA	-1	3	2	2	5	0	0	0	3
V	142	15013014	15013089	Val	AAC	-1	12	1	5	8	9	1	2	0
V	19	15156665	15156740	Asn	GTT	1	59	79	120	157	1	0	6	2
V	20	15465068	15465143	Lys	TTT	1	26	90	158	510	2	1	1	1
V	141	15465204	15465278	Pro	TGG	-1	0	2	4	2	0	0	0	0
V	140	15479964	15480038	Ala	CGC	-1	105	614	535	434	4	2	5	6
V	21	15480676	15480762	Tyr	GTA	1	27	71	114	113	2	1	0	0
V	139	15505187	15505260	Gly	GCC	-1	5	4	5	4	0	0	0	0
V	138	15515485	15515560	Val	CAC	-1	101	85	72	76	0	0	7	6
V	22	15517383	15517453	Met	CAT	1	105	62	31	45	1	0	0	0
V	137	15547853	15547937	Ser	TGA	-1	10	12	31	33	0	0	0	0
V	23	15550593	15550677	Ser	TGA	1	2	6	5	14	0	0	0	0
V	136	15551777	15551851	Asp	GTC	-1	12	8	8	8	7	2	0	1
V	24	15554011	15554085	His	GTG	1	20	40	136	189	0	3	4	2
V	25	16219139	16219213	Thr	CGT	1	8	5	7	19	5	0	0	0
V	135	16338347	16338422	Asn	GTT	-1	0	7	13	22	0	1	0	1
V	26	16338528	16338603	Asn	GTT	1	60	90	98	125	0	4	1	2
V	134	16341578	16341653	Asn	GTT	-1	1	3	1	6	0	1	1	1
V	27	16341759	16341834	Asn	GTT	1	97	57	91	84	0	2	8	2
V	28	16564041	16564116	Pseudo	CTT	1	0	0	0	0	0	0	0	0
V	133	16565109	16565184	Pseudo	CTT	-1	0	0	0	0	0	0	0	0
V	132	16592213	16592287	Gln	CTG	-1	25	50	72	130	0	0	0	1
V	131	16622035	16622110	Lys	TTT	-1	1	7	11	37	0	1	1	7
V	29	16640557	16640631	Asp	GTC	1	362	399	515	993	1	1	0	2
V	130	16645682	16645756	Asp	GTC	-1	7	7	5	15	1	0	0	0
V	129	16648742	16648816	Asp	GTC	-1	6	2	3	9	1	0	1	0
V	128	17317851	17317937	Tyr	GTA	-1	7	16	16	19	3	0	0	0
V	127	17542417	17542503	Tyr	GTA	-1	13	31	41	39	3	2	8	1
V	126	17660749	17660823	Pseudo	ATG	-1	0	2	0	1	0	0	0	0
V	30	17660857	17660933	Pseudo	TTG	1	0	1	1	1	0	0	0	1
V	31	17662145	17662220	Pseudo	ACG	1	0	0	0	0	0	0	0	0
V	125	17668331	17668406	Pseudo	TTT	-1	1	1	0	0	1	1	0	0
V	32	17686355	17686429	Pseudo	ATG	1	0	1	0	0	0	0	0	0
V	33	17686630	17686704	Pseudo	GTG	1	0	0	0	0	0	0	0	0
V	124	17688405	17688480	Pseudo	???	-1	1	0	0	1	0	0	0	0

V	34	17690482	17690556	Pseudo	ATG	1	0	0	0	0	0	0	0	0
V	35	17691257	17691331	Pseudo	GAG	1	0	1	0	2	0	0	0	0
V	123	17693770	17693845	Pseudo	TTG	-1	0	0	1	2	0	0	0	0
V	122	17694065	17694139	Pseudo	???	-1	0	0	0	0	0	0	0	0
V	121	17734607	17734681	Pseudo	TTA	-1	0	3	1	7	0	1	0	2
V	36	17734675	17734750	Pseudo	TTG	1	1	3	2	2	0	1	0	1
V	120	17739461	17739536	Pseudo	TTA	-1	0	3	18	26	0	0	2	0
V	37	17739530	17739605	Pseudo	TTG	1	1	2	2	1	0	0	2	0
V	119	17765655	17765730	Pseudo	ATG	-1	1	1	6	25	0	0	0	0
V	38	17783135	17783207	Pseudo	GGT	1	0	0	0	0	0	0	0	0
V	118	17784486	17784559	Pseudo	GAT	-1	0	0	0	1	0	0	0	0
V	117	17785685	17785759	Pseudo	GGT	-1	0	0	0	0	0	0	0	0
V	116	17786857	17786930	Pseudo	AGT	-1	0	0	0	0	0	0	0	0
V	39	17930325	17930398	Pseudo	ACC	1	0	0	0	0	0	0	0	0
V	40	17930768	17930842	Pseudo	CCC	1	1	0	0	0	0	0	0	0
V	41	17931052	17931123	Gly	GCC	1	0	0	0	1	0	0	0	1
V	42	17931668	17931741	Pseudo	GCC	1	0	0	6	5	0	0	0	0
V	43	17932735	17932808	Pseudo	ACC	1	1	0	1	1	0	0	0	0
V	44	17933304	17933378	Pseudo	GCC	1	1	1	0	0	0	1	0	0
V	45	18367111	18367185	Pseudo	ATG	1	3	2	3	3	0	0	0	0
V	46	18371201	18371276	Pseudo	ATG	1	0	0	1	0	0	0	0	0
V	115	18372850	18372925	Undet	???	-1	0	0	0	0	0	0	0	0
V	114	18373635	18373710	Pseudo	???	-1	0	0	0	0	0	0	0	0
V	113	18373932	18374008	Ile	TAT	-1	0	0	0	0	0	0	0	0
V	112	18412452	18412526	Thr	TGT	-1	2	6	11	6	1	3	2	1
V	47	18461799	18461873	Pseudo	TCG	1	0	0	1	1	0	0	0	0
V	48	18622724	18622799	Pseudo	CAA	1	0	0	0	1	0	0	0	1
V	111	18801943	18802016	Pseudo	ACC	-1	0	1	0	1	0	0	0	0
V	110	18804678	18804753	Pseudo	CGA	-1	0	0	0	1	0	0	0	0
V	109	18805534	18805607	Pseudo	GGC	-1	0	1	1	1	0	0	0	0
V	49	18825353	18825426	Pseudo	???	1	0	0	0	2	0	0	0	0
V	50	18825958	18826031	Pseudo	ACC	1	0	0	0	0	0	0	0	0
V	108	18827844	18827917	Pseudo	ACC	-1	0	0	0	1	0	0	0	0
V	107	18829016	18829089	Pseudo	ACC	-1	0	0	0	1	0	0	0	0
V	106	18906264	18906338	Pseudo	???	-1	2	2	3	12	0	0	0	0
V	105	19057473	19057547	Pseudo	GGT	-1	0	0	0	3	0	0	0	0
V	51	19058263	19058337	Pseudo	GGT	1	0	0	1	0	0	0	0	0
V	104	19058970	19059045	Pseudo	AGT	-1	0	0	0	0	0	0	0	0
V	103	19059254	19059328	Pseudo	GGT	-1	0	0	0	0	0	0	0	0
V	102	19059562	19059636	Pseudo	GGT	-1	1	0	1	3	0	0	0	0
V	101	19060477	19060551	Pseudo	GGT	-1	1	0	0	0	0	0	0	0
V	52	19082913	19082987	Pseudo	GGG	1	0	0	0	3	0	0	0	0
V	53	19084576	19084650	Pseudo	GGG	1	0	0	0	0	0	0	0	0
V	54	19126015	19126090	Phe	GAA	1	8	32	45	36	0	0	0	0
V	55	19142759	19142833	Glu	TTC	1	81	72	132	370	0	1	1	1
V	56	19347553	19347628	Pseudo	GAC	1	0	0	0	0	0	0	0	0
V	57	19393491	19393566	Pseudo	ATG	1	0	0	1	1	0	0	0	0
V	100	19394749	19394821	Pseudo	ATG	-1	2	1	0	1	1	0	0	0

V	99	19396663	19396738	Pseudo	ATG	-1	0	11	4	2	0	0	0	0
V	98	19396957	19397032	Pseudo	ATG	-1	3	71	42	42	0	0	0	0
V	58	19398559	19398632	Pseudo	ATG	1	0	1	2	0	0	1	0	0
V	59	19431262	19431337	Pseudo	TTG	1	0	0	3	5	0	0	0	0
V	97	19557606	19557681	Pseudo	???	-1	1	0	0	2	0	0	0	0
V	96	19557914	19557990	Pseudo	???	-1	1	7	5	3	0	0	0	0
V	95	19558996	19559071	Pseudo	???	-1	0	0	0	0	0	0	0	0
V	60	19577360	19577434	Pseudo	ATG	1	1	0	0	0	0	0	0	0
V	94	19578432	19578507	Pseudo	ATG	-1	1	1	2	2	0	0	0	0
V	93	19578737	19578812	Pseudo	ATG	-1	0	0	0	1	0	0	0	1
V	61	19579676	19579751	Pseudo	???	1	2	4	1	1	0	0	0	0
V	62	19581683	19581757	Pseudo	???	1	0	0	1	0	0	0	0	0
V	63	19581972	19582046	Pseudo	???	1	0	1	0	3	0	0	0	0
V	64	19584795	19584870	Pseudo	GTG	1	0	1	0	0	0	0	0	0
V	92	19587231	19587305	Pseudo	CTG	-1	0	1	0	0	0	0	0	0
V	91	19594008	19594082	Pseudo	ATG	-1	0	0	2	0	0	0	0	0
V	90	19594472	19594546	Pseudo	TTG	-1	0	1	1	1	0	0	0	0
V	89	19594772	19594845	Pseudo	ATG	-1	0	0	0	2	0	0	0	0
V	88	19595554	19595630	Pseudo	ATG	-1	0	0	0	1	0	0	0	0
V	87	19595833	19595906	Pseudo	???	-1	0	1	3	0	0	0	0	0
V	65	19596936	19597011	Pseudo	???	1	0	1	1	0	0	0	0	0
V	86	19598566	19598637	Pseudo	GGG	-1	0	1	0	0	0	0	0	0
V	66	19599485	19599561	Pseudo	GTG	1	0	1	0	2	0	0	0	0
V	85	19624010	19624086	Pseudo	ATG	-1	0	1	1	2	0	0	0	0
V	67	19629798	19629872	Pseudo	ATC	1	2	0	1	0	0	0	0	0
V	68	19656173	19656249	Pseudo	GTG	1	0	0	1	0	0	0	0	0
V	84	19665297	19665372	Pseudo	TTG	-1	0	2	0	0	0	1	0	0
V	83	19665915	19665990	Pseudo	TGG	-1	0	1	0	2	0	0	0	0
V	69	19667873	19667948	Pseudo	GGG	1	0	0	2	2	0	0	1	0
V	70	19723001	19723076	Asn	GTT	1	61	54	68	62	1	4	11	8
V	82	20016541	20016616	Pseudo	ATG	-1	0	0	0	0	0	0	0	0
V	81	20021057	20021132	Pseudo	ATG	-1	0	0	0	1	0	0	0	0
V	71	20123203	20123275	Pseudo	TTC	1	1	1	1	0	0	0	0	0
V	80	20123700	20123775	Pseudo	TTG	-1	0	1	0	1	0	0	0	0
V	72	20123814	20123889	Pseudo	TTG	1	0	0	0	0	0	0	0	0
V	79	20126136	20126211	Pseudo	TTG	-1	1	2	0	1	0	1	0	0
V	73	20134972	20135047	Pseudo	TTA	1	1	0	2	4	0	0	0	0
V	78	20214831	20214914	Pseudo	TTG	-1	0	1	0	2	0	0	0	0
V	74	20219270	20219342	His	ATG	1	0	0	0	0	0	0	0	0
V	77	20347763	20347838	Asn	GTT	-1	4	3	6	7	0	1	0	0
V	76	20350495	20350570	Asn	GTT	-1	3	4	3	5	2	1	1	0
V	75	20694087	20694161	Cys	GCA	-1	0	2	15	10	0	0	0	0
X	1	86887	86961	Pseudo	CTC	1	1	0	0	1	1	0	0	0
X	2	288447	288524	Pseudo	CTT	1	0	0	0	1	0	0	0	0
X	304	289510	289587	Pseudo	CTT	-1	0	2	0	0	0	0	0	0
X	303	353252	353336	Leu	AAG	-1	40	46	140	238	0	0	4	5
X	3	410392	410466	Gln	TTG	1	31	44	135	355	0	0	2	2
X	4	487816	487890	Thr	TGT	1	3	21	43	86	0	0	0	2

X	302	489866	489940	Asp	GTC	-1	4	5	4	4	2	0	1	1
X	301	865385	865471	Tyr	GTA	-1	8	28	31	9	1	7	8	1
X	300	1089167	1089242	Arg	TCG	-1	24	21	40	45	0	0	0	0
X	5	1089397	1089474	Arg	TCG	1	9	52	52	45	0	0	1	0
X	6	1089835	1089910	Arg	TCG	1	147	94	98	140	0	0	1	1
X	299	1379595	1379669	Gly	TCC	-1	0	3	5	6	0	3	5	6
X	298	1433055	1433138	Pseudo	???	-1	3	1	4	2	0	1	3	1
X	297	1473998	1474082	Pseudo	ATC	-1	8	13	41	50	7	13	38	45
X	296	1589659	1589735	Ile	AAT	-1	4	20	34	60	0	1	1	0
X	295	1590072	1590148	Ile	AAT	-1	5	7	9	17	1	1	3	3
X	294	1590500	1590576	Ile	AAT	-1	4	6	8	18	1	1	1	2
X	7	1620270	1620353	Pseudo	ATC	1	0	2	2	4	0	2	2	4
X	293	1629873	1629947	Arg	CCG	-1	3	11	31	50	0	0	1	0
X	8	1643845	1643920	Pseudo	ATC	1	29	35	88	88	16	28	74	81
X	292	1691075	1691145	Pseudo	CGA	-1	4	5	6	14	0	0	2	0
X	9	1692729	1692799	Pseudo	CGA	1	21	28	34	83	0	0	0	1
X	10	1698669	1698744	Arg	TCG	1	126	119	146	187	0	0	0	0
X	291	1764246	1764322	Arg	CCT	-1	0	1	1	2	0	0	0	0
X	11	1775483	1775558	Pseudo	CCT	1	1	2	3	0	0	0	0	0
X	12	1830647	1830744	Pseudo	TAT	1	1	1	4	10	0	0	2	1
X	13	1858813	1858887	Glu	TTC	1	64	103	293	971	0	0	1	0
X	290	1880808	1880882	Pro	CGG	-1	245	733	1896	2372	0	3	4	8
X	14	1891440	1891514	Pseudo	TAA	1	0	0	1	0	0	0	0	0
X	289	2125753	2125827	Pseudo	???	-1	0	0	0	0	0	0	0	0
X	288	2227180	2227254	Asp	GTC	-1	3	3	3	10	1	3	0	0
X	15	2462547	2462622	Pseudo	CTT	1	0	0	1	0	0	0	0	0
X	287	2463475	2463550	Pseudo	CTT	-1	0	0	0	0	0	0	0	0
X	286	2526208	2526284	Ile	AAT	-1	39	32	13	38	1	1	1	3
X	16	2528035	2528109	Thr	AGT	1	244	362	362	645	1	2	1	4
X	17	2531354	2531430	Ile	AAT	1	77	64	105	230	3	1	2	2
X	285	2549518	2549593	Asn	GTT	-1	1	11	19	20	0	5	1	5
X	18	2553558	2553632	Asp	GTC	1	45	104	116	196	7	2	14	10
X	19	2613235	2613308	Gly	GCC	1	185	156	243	447	1	3	1	4
X	284	3035963	3036037	Glu	CTC	-1	124	110	35	39	0	0	4	5
X	20	3036154	3036228	Gly	TCC	1	40	46	33	72	2	0	1	0
X	283	3371406	3371480	Thr	AGT	-1	1	1	3	6	1	0	0	1
X	21	3697743	3697818	Met	CAT	1	198	206	93	156	0	0	0	0
X	282	3793545	3793620	Val	TAC	-1	147	117	117	134	0	1	2	2
X	22	3969536	3969610	Pro	TGG	1	23	18	23	56	0	0	2	2
X	23	3970076	3970150	Pro	AGG	1	132	285	680	920	0	3	4	5
X	24	3970245	3970319	Pro	AGG	1	132	278	694	900	2	1	6	2
X	25	3994581	3994660	Gly	TCC	1	12	34	36	63	5	5	2	3
X	26	4242499	4242578	Pseudo	AAT	1	32	47	144	193	0	1	3	1
X	27	4409759	4409833	Glu	CTC	1	43	74	113	219	0	3	5	4
X	28	4410627	4410701	Glu	CTC	1	32	72	107	210	0	2	2	0
X	29	4444237	4444311	Glu	TTC	1	99	111	209	491	1	2	2	2
X	30	4444989	4445063	Thr	AGT	1	226	398	358	618	2	1	0	4
X	31	4789102	4789177	Lys	TTT	1	37	111	224	507	0	0	1	0

X	281	5022783	5022857	His	GTG	-1	2	5	20	39	0	1	1	4
X	280	5085588	5085664	Ile	AAT	-1	9	39	51	107	3	22	18	47
X	32	5284387	5284464	Pseudo	CTT	1	0	0	1	0	0	0	1	0
X	279	5285444	5285521	Pseudo	CTT	-1	0	0	0	0	0	0	0	0
X	278	5319350	5319425	Phe	GAA	-1	3	10	13	9	0	0	0	0
X	277	5407378	5407453	Met	CAT	-1	6	13	8	14	0	1	1	0
X	276	5451169	5451244	Lys	TTT	-1	0	4	7	24	0	1	1	0
X	33	5485080	5485155	Met	CAT	1	162	204	113	130	0	0	0	0
X	34	5485423	5485498	Met	CAT	1	184	182	106	147	0	0	0	0
X	35	5485714	5485789	Met	CAT	1	207	216	96	126	0	0	0	0
X	36	5707993	5708077	Ser	GCT	1	26	123	271	1064	0	8	52	51
X	37	5709340	5709424	Ser	GCT	1	28	137	209	1046	1	1	0	2
X	38	5713617	5713692	Lys	TTT	1	42	75	205	205	0	0	4	2
X	275	5801173	5801247	Pro	TGG	-1	0	3	2	2	0	0	0	1
X	39	5801437	5801511	Pro	TGG	1	17	9	22	32	0	0	0	1
X	274	6220032	6220107	Arg	ACG	-1	234	478	199	115	5	1	6	14
X	40	6286285	6286359	Ala	TGC	1	146	329	202	192	0	0	3	2
X	273	6292239	6292313	Ala	TGC	-1	5	10	11	10	2	0	4	4
X	41	6371342	6371416	Trp	CCA	1	202	376	498	400	10	4	12	12
X	42	6593559	6593634	Phe	GAA	1	17	34	25	48	0	2	1	0
X	43	6600191	6600266	Pseudo	CTT	1	0	0	0	0	0	0	0	0
X	272	6601269	6601344	Pseudo	CTT	-1	0	0	0	0	0	0	0	0
X	271	6903071	6903155	Leu	AAG	-1	12	32	64	257	0	0	5	5
X	270	7176937	7177011	Thr	AGT	-1	1	1	1	1	0	0	0	0
X	44	7323707	7323781	Ala	AGC	1	735	539	382	475	0	3	1	5
X	269	7378735	7378809	Ala	AGC	-1	6	10	6	6	0	0	0	0
X	268	7507242	7507316	Ala	AGC	-1	25	14	21	30	0	0	1	3
X	45	7680989	7681063	Glu	CTC	1	198	117	110	240	0	1	2	6
X	46	7773139	7773214	Asn	GTT	1	3	6	24	15	1	0	0	3
X	47	7773667	7773742	Asn	GTT	1	50	10	24	25	0	1	5	6
X	267	7773911	7773986	Asn	GTT	-1	2	9	4	6	0	0	0	3
X	266	7794282	7794356	Gly	TCC	-1	5	6	2	9	5	4	0	4
X	48	7794552	7794626	Gly	TCC	1	49	41	33	67	1	1	3	1
X	265	7898257	7898331	Glu	CTC	-1	87	96	18	22	0	1	0	0
X	49	7906537	7906611	Glu	CTC	1	140	91	82	189	0	1	0	0
X	264	8036633	8036708	Arg	ACG	-1	277	389	265	116	0	4	2	4
X	50	8037458	8037532	Pro	AGG	1	4	47	90	47	1	0	2	0
X	51	8149107	8149181	Asp	GTC	1	86	156	249	355	0	0	0	1
X	263	8152494	8152568	Asp	GTC	-1	4	13	3	6	2	1	1	1
X	262	8152760	8152844	Ser	AGA	-1	2	0	2	4	1	0	2	1
X	52	8154396	8154480	Ser	AGA	1	594	267	272	241	0	1	3	0
X	53	8154673	8154747	Asp	GTC	1	330	393	420	893	2	0	2	0
X	54	8155413	8155497	Ser	AGA	1	126	132	193	642	0	4	1	1
X	55	8155703	8155777	Asp	GTC	1	29	143	258	324	7	11	3	1
X	56	8156129	8156203	Met	CAT	1	251	134	217	280	1	2	1	2
X	261	8156582	8156658	Ile	AAT	-1	1	4	9	32	0	1	2	5
X	260	8381739	8381814	Arg	TCT	-1	18	17	17	24	1	2	0	0
X	57	8386151	8386226	Arg	TCT	1	20	33	24	22	0	0	0	0

X	58	8386561	8386636	Arg	TCT	1	29	32	33	36	0	1	1	1
X	59	8404446	8404521	Asn	GTT	1	89	106	140	185	0	4	6	4
X	259	8409240	8409324	Leu	AAG	-1	0	2	6	11	0	0	0	0
X	258	8417856	8417940	Leu	AAG	-1	8	19	53	217	1	0	5	2
X	257	8419358	8419442	Leu	AAG	-1	19	21	46	115	0	0	1	2
X	256	8458983	8459055	Pseudo	CTT	-1	0	3	2	0	0	3	2	0
X	255	8608779	8608853	Gln	CTG	-1	17	24	72	115	0	0	0	0
X	60	8608986	8609060	Gln	TTG	1	189	255	597	1853	1	1	6	16
X	61	8623551	8623626	Arg	ACG	1	492	941	577	467	0	2	0	5
X	254	8623709	8623783	Cys	GCA	-1	0	5	26	18	0	0	0	1
X	253	8623943	8624017	Cys	GCA	-1	4	8	12	10	0	0	1	0
X	62	8624230	8624304	Cys	GCA	1	28	86	69	61	0	0	0	2
X	252	8624405	8624480	Arg	ACG	-1	118	240	159	97	0	0	0	0
X	251	8638565	8638639	Cys	GCA	-1	1	4	24	17	1	0	1	1
X	63	8638870	8638944	Cys	GCA	1	21	73	78	50	0	0	0	1
X	250	8639170	8639243	Arg	ACG	-1	97	255	211	185	0	0	0	1
X	249	8640739	8640814	Arg	ACG	-1	131	283	117	92	0	0	4	0
X	64	8646428	8646502	Glu	TTC	1	105	116	219	467	1	2	1	1
X	248	8650654	8650729	Val	AAC	-1	10	17	23	15	8	12	13	10
X	247	8796766	8796839	Gly	GCC	-1	3	0	10	6	0	0	3	2
X	246	8818044	8818118	Cys	GCA	-1	0	2	1	1	0	1	0	0
X	245	8818522	8818596	Gly	TCC	-1	2	2	4	6	2	0	3	3
X	65	8819628	8819702	Gly	TCC	1	31	21	4	12	2	0	1	1
X	244	8819868	8819942	Gly	TCC	-1	1	2	6	4	0	0	1	1
X	66	8820132	8820206	Gly	TCC	1	18	25	3	8	1	1	1	7
X	67	8836060	8836134	Glu	TTC	1	105	97	167	370	1	0	3	1
X	243	8838282	8838356	Gly	TCC	-1	3	3	6	4	2	2	1	1
X	242	8846449	8846524	Gly	TCC	-1	2	1	1	2	1	0	1	2
X	68	8846668	8846742	Gly	TCC	1	9	6	6	12	0	2	0	0
X	69	8877287	8877361	Trp	CCA	1	199	428	471	428	6	1	10	9
X	70	8925588	8925662	Gln	TTG	1	136	204	481	1502	0	0	0	0
X	71	8939496	8939580	Leu	AAG	1	238	293	803	1373	0	0	2	4
X	241	8944175	8944249	Trp	CCA	-1	12	39	36	44	4	11	18	19
X	240	8945103	8945177	Trp	CCA	-1	9	26	13	30	3	0	2	9
X	239	8955863	8955947	Leu	AAG	-1	10	26	121	90	0	0	1	3
X	238	8969431	8969505	Pro	TGG	-1	1	1	3	0	0	0	0	0
X	72	8969684	8969758	Pro	TGG	1	33	114	127	106	0	0	0	1
X	73	8971546	8971630	Ser	AGA	1	476	193	222	247	5	2	5	1
X	237	9024355	9024429	Thr	TGT	-1	1	4	16	44	0	0	1	0
X	236	9025605	9025679	Thr	CGT	-1	1	0	4	3	0	0	0	0
X	235	9059222	9059339	Undet	???	-1	4	21	18	73	0	4	0	2
X	234	9074614	9074688	Gln	TTG	-1	3	3	8	15	1	0	0	0
X	233	9078196	9078270	Gln	TTG	-1	5	6	11	11	1	0	1	0
X	232	9090568	9090642	Gln	TTG	-1	0	5	11	9	0	0	0	1
X	74	9090774	9090848	Gln	CTG	1	67	104	235	527	0	1	3	1
X	231	9091038	9091112	Gln	TTG	-1	1	3	1	4	0	0	0	0
X	75	9091254	9091328	Gln	TTG	1	50	58	137	397	0	0	0	3
X	230	9091498	9091572	Gln	TTG	-1	1	3	2	2	0	0	0	0

X	76	9237752	9237826	Ala	AGC	1	751	830	828	1058	1	0	1	2
X	77	9238805	9238879	Ala	AGC	1	221	255	306	424	1	6	3	27
X	229	9373088	9373162	Met	CAT	-1	8	15	2	9	0	2	0	2
X	228	9467667	9467741	Pro	TGG	-1	1	3	1	4	0	0	0	0
X	227	9561028	9561103	Val	AAC	-1	18	14	23	38	6	2	7	19
X	226	9622764	9622838	Ala	AGC	-1	8	13	20	28	1	1	0	2
X	225	9637792	9637866	Ala	AGC	-1	7	18	10	18	1	0	2	1
X	78	9743531	9743605	Ala	TGC	1	101	57	64	83	0	1	0	0
X	79	9743689	9743763	Ala	AGC	1	7	3	1	1	1	1	1	1
X	224	9829606	9829693	Ile	TAT	-1	3	1	0	2	0	0	0	0
X	223	10018364	10018439	Arg	ACG	-1	102	252	101	81	0	0	1	1
X	80	10124439	10124523	Ser	TGA	1	14	35	71	63	0	0	0	0
X	81	10132619	10132694	Arg	ACG	1	495	1064	650	435	0	2	2	3
X	222	10132909	10132984	Arg	ACG	-1	0	6	10	4	0	0	0	0
X	82	10145097	10145172	Arg	ACG	1	476	1055	627	454	0	0	2	1
X	221	10148587	10148662	Arg	ACG	-1	21	131	45	48	0	0	0	0
X	220	10666956	10667030	Glu	TTC	-1	6	2	2	10	1	0	0	1
X	83	10667530	10667604	Glu	TTC	1	51	95	308	962	3	1	0	0
X	219	10731921	10731996	Arg	ACG	-1	1	2	4	1	0	1	1	0
X	218	10821259	10821333	Glu	TTC	-1	5	3	8	14	0	0	0	1
X	84	10963762	10963836	Gly	TCC	1	13	13	24	35	0	0	4	5
X	85	11095607	11095691	Leu	TAG	1	22	84	204	766	0	1	1	4
X	217	11162850	11162934	Leu	AAG	-1	4	10	33	83	0	0	4	2
X	216	11163289	11163373	Leu	AAG	-1	1	20	47	169	0	1	6	6
X	215	11252522	11252597	Arg	ACG	-1	14	132	135	141	0	0	0	5
X	86	11645616	11645691	Asn	GTT	1	67	58	79	77	0	2	5	6
X	214	11757761	11757847	Leu	TAA	-1	39	63	129	330	0	0	0	5
X	213	11806517	11806592	Arg	TCG	-1	24	25	21	22	0	0	4	2
X	212	11872170	11872244	Gln	TTG	-1	1	3	8	8	0	1	0	0
X	87	11872438	11872512	Gln	CTG	1	140	228	494	1531	0	1	0	0
X	88	11933660	11933734	Thr	CGT	1	21	46	48	98	4	0	0	1
X	211	11935009	11935083	Thr	CGT	-1	16	25	24	38	0	1	0	0
X	210	11993450	11993524	His	GTG	-1	0	0	1	11	0	0	0	2
X	209	12106076	12106151	Lys	CTT	-1	2	3	2	6	2	0	1	0
X	89	12166152	12166227	Pseudo	CTT	1	0	1	0	0	0	1	0	0
X	208	12167039	12167114	Pseudo	CTT	-1	0	0	0	0	0	0	0	0
X	207	12337927	12338002	Arg	TCG	-1	73	57	100	189	1	1	0	1
X	206	12347936	12348058	Leu	CAA	-1	3	8	14	48	0	1	1	4
X	205	12367571	12367645	Glu	TTC	-1	2	1	7	7	0	0	0	0
X	204	12458105	12458179	Ala	AGC	-1	10	16	11	20	1	3	1	2
X	90	12563719	12563793	Thr	TGT	1	7	30	47	73	0	0	1	2
X	91	12661093	12661179	Ile	TAT	1	33	208	562	1377	2	18	37	42
X	92	12665157	12665243	Tyr	GTA	1	23	45	111	211	1	3	7	0
X	93	12843618	12843693	Val	AAC	1	145	171	304	503	9	13	31	59
X	203	12854767	12854841	Pro	TGG	-1	6	6	7	8	3	2	2	0
X	94	12889870	12889954	Ser	TGA	1	82	150	102	200	0	1	0	0
X	202	12890212	12890286	Ala	CGC	-1	118	1703	1130	632	0	0	3	5
X	95	12937631	12937705	Gln	TTG	1	118	197	542	1668	0	0	1	2

X	96	12938112	12938186	Gln	TTG	1	29	42	106	229	0	0	0	1
X	97	12942388	12942472	Leu	AAG	1	207	276	795	1411	0	1	5	9
X	201	13038888	13038974	Leu	CAG	-1	1520	876	467	411	3	2	3	9
X	200	13046294	13046378	Ser	CGA	-1	2	8	13	13	1	0	0	1
X	98	13046509	13046593	Ser	AGA	1	640	259	242	238	0	5	5	4
X	199	13121188	13121261	Gly	GCC	-1	4	5	4	9	0	0	0	1
X	99	13261134	13261208	Thr	AGT	1	177	330	326	642	0	2	0	2
X	198	13264611	13264697	Tyr	GTA	-1	3	11	18	16	0	2	1	3
X	197	13283565	13283639	Thr	AGT	-1	0	0	1	5	0	0	0	0
X	196	13284694	13284780	Tyr	GTA	-1	39	32	16	14	34	21	2	2
X	100	13293123	13293197	Thr	AGT	1	77	85	81	133	0	2	0	2
X	101	13306965	13307040	Val	TAC	1	144	139	139	145	4	3	9	14
X	102	13307961	13308035	Pro	TGG	1	6	8	5	6	0	0	0	0
X	195	13415049	13415125	Ile	AAT	-1	0	1	6	3	0	1	5	2
X	103	13435898	13435974	Ile	AAT	1	104	515	843	2095	1	5	3	7
X	104	13442575	13442659	Ser	GCT	1	27	121	229	1007	0	0	1	3
X	194	13443315	13443436	Leu	CAA	-1	15	18	34	60	0	1	7	1
X	105	13448609	13448683	Glu	CTC	1	92	103	145	316	0	2	4	8
X	193	13448802	13448886	Ser	GCT	-1	0	7	11	36	0	0	0	1
X	192	13511897	13511971	Pro	TGG	-1	0	1	0	0	0	0	0	0
X	106	13653658	13653732	Thr	TGT	1	8	24	47	72	1	0	0	0
X	191	13655844	13655919	Lys	CTT	-1	2	5	3	3	0	4	1	1
X	107	13665485	13665606	Leu	CAA	1	62	152	290	877	0	3	3	2
X	108	13714819	13714894	Lys	CTT	1	31	83	139	211	1	1	2	0
X	109	13716424	13716499	Lys	CTT	1	8	19	49	50	1	1	1	0
X	110	13717126	13717201	Lys	CTT	1	18	62	119	179	0	3	2	0
X	111	13729836	13729911	Lys	CTT	1	10	39	122	181	1	0	0	0
X	112	13730288	13730363	Lys	CTT	1	20	44	135	196	0	1	0	1
X	113	13730709	13730784	Lys	CTT	1	39	177	561	671	0	0	1	1
X	114	13781572	13781647	Lys	CTT	1	9	21	73	115	0	0	0	0
X	115	13782181	13782256	Lys	CTT	1	46	184	546	639	0	1	0	3
X	190	13886298	13886373	Pseudo	TCC	-1	1	1	1	0	0	0	0	0
X	116	14010184	14010258	Gly	TCC	1	36	25	27	53	0	0	0	0
X	117	14015875	14015949	Glu	CTC	1	1	4	9	8	0	2	3	7
X	118	14061603	14061680	Pseudo	CTT	1	1	0	0	0	1	0	0	0
X	189	14062367	14062444	Pseudo	CTT	-1	0	0	0	0	0	0	0	0
X	188	14063874	14063949	Lys	TTT	-1	0	4	12	9	0	1	3	0
X	119	14115622	14115697	Arg	CCT	1	46	125	134	223	0	3	5	2
X	120	14134976	14135050	His	GTG	1	23	52	64	175	6	19	12	43
X	187	14135236	14135310	His	GTG	-1	1	0	0	8	1	0	0	2
X	121	14135805	14135879	His	GTG	1	24	40	71	134	5	17	24	35
X	186	14136052	14136126	His	GTG	-1	0	2	1	5	0	1	1	0
X	122	14136621	14136695	His	GTG	1	17	38	59	97	4	15	20	36
X	185	14136867	14136941	His	GTG	-1	0	0	3	5	0	0	0	0
X	184	14161954	14162028	Asp	GTC	-1	20	4	10	10	17	1	3	4
X	123	14162146	14162230	Ser	AGA	1	119	145	224	663	0	6	5	7
X	183	14228848	14228922	Trp	CCA	-1	13	16	27	24	10	9	17	13
X	182	14437800	14437874	Pro	TGG	-1	2	0	0	0	0	0	0	0

X	124	14438032	14438106	Pro	TGG	1	20	35	47	70	0	0	0	0
X	181	14478804	14478879	Lys	CTT	-1	2	0	0	4	2	0	0	0
X	180	14486138	14486213	Lys	CTT	-1	2	5	5	14	0	0	0	0
X	125	14679091	14679167	Ile	AAT	1	123	480	820	2123	0	4	5	2
X	126	14804808	14804882	Cys	GCA	1	29	53	51	43	0	0	0	0
X	179	15146489	15146564	Arg	TCT	-1	11	10	14	21	0	0	1	0
X	178	15200745	15200819	Gly	TCC	-1	0	2	0	3	0	2	0	3
X	127	15200938	15201012	Gly	TCC	1	43	43	35	71	0	1	2	0
X	128	15236055	15236130	Val	AAC	1	133	148	288	515	1	4	26	45
X	177	15241283	15241358	Lys	CTT	-1	0	2	3	3	0	2	0	0
X	129	15248904	15248977	Pseudo	CGT	1	18	23	33	60	1	0	0	0
X	130	15297402	15297478	Ile	AAT	1	114	86	173	210	0	1	4	7
X	131	15368606	15368680	Pseudo	TTC	1	0	1	1	2	0	0	0	1
X	132	15741982	15742068	Leu	CAG	1	87	63	101	139	29	19	33	20
X	176	15747735	15747801	Pro	TGG	-1	1	2	0	1	0	0	0	0
X	133	15747963	15748036	Pro	TGG	1	8	27	19	25	0	0	0	0
X	175	15748245	15748319	Pseudo	TGG	-1	1	3	1	3	0	0	0	0
X	134	15751352	15751426	Pro	TGG	1	11	20	35	67	1	0	0	0
X	174	15769938	15770022	Ser	AGA	-1	21	19	7	23	0	1	0	1
X	173	15773260	15773344	Ser	CGA	-1	1	3	3	3	0	2	0	0
X	135	15773459	15773543	Ser	AGA	1	31	40	172	611	1	1	2	11
X	172	15796552	15796626	Pro	TGG	-1	1	1	0	3	0	0	0	1
X	136	15796787	15796861	Pro	TGG	1	25	69	121	104	0	0	0	0
X	171	16042533	16042617	Ser	TGA	-1	8	11	32	52	3	2	0	2
X	170	16208188	16208274	Tyr	GTA	-1	2	8	6	8	2	3	2	2
X	169	16208750	16208836	Tyr	GTA	-1	13	20	30	7	2	7	6	0
X	168	16209153	16209239	Tyr	GTA	-1	1	4	13	16	1	1	1	0
X	167	16209694	16209780	Tyr	GTA	-1	6	8	16	11	0	1	0	0
X	166	16217820	16217895	Lys	TTT	-1	1	2	14	13	1	1	0	0
X	165	16271220	16271294	Glu	CTC	-1	1	8	4	6	0	2	1	0
X	137	16273694	16273768	Glu	CTC	1	74	19	33	89	0	0	1	0
X	138	16274425	16274499	Glu	CTC	1	23	38	57	119	0	0	0	0
X	164	16284247	16284321	Glu	CTC	-1	2	4	9	8	0	1	0	0
X	163	16285219	16285293	Glu	CTC	-1	2	3	2	8	0	0	0	0
X	139	16298269	16298344	Val	AAC	1	70	432	531	362	4	3	6	5
X	140	16299321	16299396	Val	AAC	1	55	485	585	375	3	6	7	8
X	141	16299991	16300066	Val	AAC	1	76	351	501	416	2	12	30	45
X	142	16300517	16300592	Val	AAC	1	50	419	488	316	1	2	4	4
X	143	16378041	16378115	Glu	CTC	1	31	55	101	226	0	0	1	0
X	144	16441074	16441148	Met	CAT	1	148	119	218	455	1	1	1	3
X	145	16461405	16461479	Arg	TCG	1	135	92	125	137	0	0	1	4
X	162	16503068	16503143	Phe	GAA	-1	3	12	22	8	0	0	0	0
X	146	16522697	16522771	Pseudo	GAA	1	7	32	64	29	0	1	0	0
X	147	16523271	16523346	Phe	GAA	1	10	32	21	37	0	2	0	0
X	148	16523850	16523925	Phe	GAA	1	16	20	30	23	1	2	1	0
X	149	16551438	16551513	Phe	GAA	1	9	16	19	15	0	2	0	0
X	150	16558869	16558943	Glu	CTC	1	142	171	102	230	0	1	8	4
X	151	16590322	16590397	Val	CAC	1	107	134	130	156	0	1	0	1

X	161	16590855	16590930	Val	CAC	-1	63	71	62	86	1	0	1	7
X	160	16604644	16604718	Asp	GTC	-1	6	10	7	6	4	4	4	0
X	159	16617816	16617890	Asp	GTC	-1	18	7	8	7	7	5	5	1
X	158	16618220	16618294	Asp	GTC	-1	4	1	2	4	4	1	1	1
X	157	16619257	16619331	Asp	GTC	-1	5	1	2	6	1	1	0	1
X	156	16634754	16634828	His	GTG	-1	1	1	3	4	1	1	2	2
X	155	16689909	16689993	Ser	GCT	-1	1	4	14	21	0	1	2	0
X	154	16928195	16928268	Gly	GCC	-1	6	17	65	177	0	0	0	0
X	152	17611213	17611288	Val	AAC	1	37	56	102	151	3	7	9	23
X	153	17611424	17611499	Val	AAC	-1	14	23	80	77	2	5	14	19

Supplementary Table S2. Count of sequencing reads aligned to introns of tRNA genes

Chr	tRNA	tRNA Intron Bounds		tRNA	Anticodon	Mean Read Alignment in tRNA	Intron Alignment			
#	#	Start	End	Isotype			Day0	Day5	Day8	Day12
I	6	6135868	6135879	Tyr	GTA	175	0	1	0	0
II	53	9211776	9211787	Tyr	GTA	71	0	0	1	0
III	1	535416	535427	Tyr	GTA	167	0	1	1	2
III	25	3195464	3195475	Tyr	GTA	182	0	1	1	0
III	27	4362533	4362569	Leu	CAA	333	0	0	2	0
III	37	8109574	8109612	Leu	CAA	342	0	0	0	0
III	39	8639263	8639299	Leu	CAA	401	0	0	0	1
III	49	13224501	13224512	Tyr	GTA	186	0	1	1	0
IV	73	11928500	11928535	Leu	CAA	36	0	2	6	5
V	9	6917485	6917496	Ile	TAT	532	0	0	0	0
V	11	8247497	8247508	Tyr	GTA	65	0	0	0	0
V	154	9412213	9412224	Tyr	GTA	33	0	0	0	0
V	15	12002194	12002205	Ile	TAT	538	0	0	0	0
V	18	14689553	14689564	Tyr	GTA	99	0	0	0	0
V	21	15480712	15480723	Tyr	GTA	81	0	0	0	0
V	128	17317886	17317897	Tyr	GTA	15	0	1	5	6
V	127	17542452	17542463	Tyr	GTA	31	0	0	2	7
X	301	865420	865431	Tyr	GTA	19	0	0	0	0
X	7	1620305	1620315	Pseudo	ATC	2	0	0	0	0
X	12	1830683	1830706	Pseudo	TAT	4	0	0	0	0
X	235	9059262	9059304	Undet	???	29	0	0	0	1
X	206	12347984	12348020	Leu	CAA	18	0	0	2	3
X	91	12661130	12661140	Ile	TAT	545	0	0	0	0
X	92	12665193	12665204	Tyr	GTA	98	0	0	1	4
X	198	13264646	13264657	Tyr	GTA	12	0	4	11	7
X	196	13284729	13284740	Tyr	GTA	25	0	2	2	2
X	194	13443363	13443398	Leu	CAA	32	0	0	0	0
X	107	13665522	13665557	Leu	CAA	345	0	0	1	0
X	170	16208223	16208234	Tyr	GTA	6	0	0	1	2
X	169	16208785	16208796	Tyr	GTA	18	1	0	2	1
X	168	16209188	16209199	Tyr	GTA	9	0	0	1	2
X	167	16209729	16209740	Tyr	GTA	10	0	0	1	0

Supplementary Table S3. Unique mature tRNA sequences

tRNA Isotype	Anticodon	Full seq + 'CCA'	Chr #	tRNA Bounds Start	tRNA Bounds End	Strand Dir	Homology Group (k =30)
Phe	GAA	GCCTCGATAGCTCAGTTGGGAGAGCGCACGACTGAAGATCGTGAGGTCACCAGTTCGATCCTGGTTCGGGGCACCA	II	9713162	9713237	-1	1
Phe	GAA	GCCTCGATAGCTCAGTTGGGGTGAGCGTACGACTGAAAATCGTTAGGTCACCAGTTCGATCCTGGTTCGGGCACCA	I	10945814	10945889	1	1
Phe	GAA	GCCTCGATAGCTCAGTTGGGAGAGCGTACGACTGAAGATCGTAAGGTCACCAGTTCGATCCTGGTTCGGGGCACCA	III	8652974	8653049	1	1
Phe	GAA	GCCTCAATAGCTCAGTTGGGAGAGCGTACGACTGAAGATCGTAAGGTCACCAGTTCGATCCTGGTTTGGGGCACCA	X	5319350	5319425	-1	1
Tyr	GTA	CCGTCGATAGCTCAGTAGTTGGTAGAGCGGAGGACTGTAGATCCTTAGATCGCTGGTTCGAATCCGGCTCGACGGACCA	V	9412178	9412264	-1	2
Tyr	GTA	CCGTCGATAGCTCAGTTGGTAGAGCGGAGGACTGTAGATCCTTAGGTCACTGGTTCGAATCCGGTTGGACGGACCA	X	13284694	13284780	-1	2
Tyr	GTA	CCGTCGATAGCTCAGTTGGTAGAGCGGAGGACTGTAGATCCTTAGGTCACTGGTTCGAATCCGGTTCGACGGACCA	X	12665157	12665243	1	2
Tyr	GTA	CCGTCGATAGCTCAGTTGGTAGAGCGCAGGACTGTAGATCCTTAGGTCACTGGTTCGAATCCGGTTCGACGGACCA	V	17317851	17317937	-1	2
Tyr	GTA	CCTGTGAGCTCAGTTGGTAGAGCGGAGGACTGTAGATCCTTAGGTCACTGGTTCGAATCCGGTTCGACGGACCA	III	3195428	3195514	1	2
Tyr	GTA	CCGTCGATAGCTCAGTTGGTAGAGCGGAGGACTGTAGATCCTTAGGTCGCTGGTTCGAATCCGGCTCGACGGACCA	V	8247461	8247547	1	2
Tyr	GTA	CAGTCGATAGCTCAGTTGGTAGAGCGGAGGACTGTAGATCCTTAGGTCGCTGGTTCGAATCCGGCTCGACGGACCA	II	9211741	9211827	-1	2
Lys	CTT	GCCCGGTTAGCTCAGTCTACCGACTGCACCAGACTCTTAATCTGGTTGTCGTGGGTTCGAGTCCCGCATTGGGCTCCA	II	9773597	9773672	1	3
Lys	CTT	GCCCGGTTAGCTCAGTCGGTAGAGCACCAGACTCTTAATCTGGTTGTCGCGGGTTCGAGCCCCGCATTGGGCTCCA	I	6136599	6136674	1	3
Lys	CTT	GCCCGGTTAGCTCAGTCGGTAGAGCACCAGACTCTTAATCTGGTTCTCGCGGGTTCGAGCCCCGCATTGGGCTCCA	X	13729836	13729911	1	3
Lys	CTT	GCCTGGTTAGCTCAGTCGGTAGAGCACCAGACTCTTAATCTGGTTGTCGCGGTTTCGAGCCCCGCATTGGGCTCCA	X	13716424	13716499	1	3
Lys	CTT	GCCCGGTTAGCTCAGCCGGTAGAGCACCAGACTCTTAATCTGGTTGTCGCGGGTTCGAGCCCCGCATTGGGCTCCA	I	7279350	7279425	-1	3
Lys	CTT	GTAAAAATAGCTCAGTCGGTAGAGCACCAGACTCTTAATCTGGTTGTCGCGGGTTCGAGCCCCGCATTGGGCTCCA	X	14486138	14486213	-1	3
Thr	AGT	GCCTCATTGGCTCAGTGGCAGAGCGTCTGTCTAGTAAACAGAAGGTCGCTGGTTCGATTCCAGCATGAGGCACCA	II	3567610	3567684	-1	4
Thr	AGT	GCCTCATTGGCTCAGTGGCAGAGCGTCTGTCTAGTAAACAGAAGGTCGCTGGTTCGATTCCAGCATGAGGCCCCA	II	3519086	3519160	-1	4
Thr	CGT	GCCCGTATAGCTCAGAGGCAGAGCGTCTGTCTCGTAAACAGAAGGTCGGCGGTTCAATCCCGCCTGTGGGCACCA	X	11933660	11933734	1	4
Thr	CGT	GCCCGTATAGCTCAGTGGCAGAGCGTCTGTCTCGTAAACAGAAGGTCGACGGTTCAATCCCGTCTGGGGGCACCA	V	7730632	7730706	-1	4
Thr	CGT	GCCCGTATAGCTCAGTGGCAGAGCGTCTGTCTCGTAAACAGAAGGTCGGCGGTTCAATCCCGCCTGGGGGCACCA	III	11792682	11792756	1	4
Thr	CGT	GCCCGTATAGCTCAGTGGCAGAGCGTCTGTCTCGTAAACAGAAGGCCGGCGGTTCAATCCCGCCTGGGGGCACCA	V	16219139	16219213	1	4
Ile	AAT	GCCGCCATAGCTCAGTCGGTTAGAGCGTGGGTCTAATAAGCCCAAGGTCGCAGGTTCGACCCCTGCTGGCGGCACCA	I	6164193	6164269	1	5
Ile	AAT	GCCGCCATAGCCCAGTCGGTTAGAGCGTGGGTCTAATAAGCCCAAGGTCGCAGGTTCGACCCCTGCTGGCGGCACCA	X	2531354	2531430	1	5
Thr	CGT	GCCCTTATAGCTCAGTGGTAGAGCGTTGGTCTCGTAAACCAAAGGTCCGTAGTTCAATCCTGCGTGAGGGCACCA	X	9025605	9025679	-1	5
Thr	TGT	GCCCTTATAGCTCAGTGGTAGAGCGTTGGTCTTGTAAACCAAAGGTCCGTAGTTCAATCCTGCGTGGGGGCACCA	I	13310331	13310405	-1	5
Lys	TTT	GCCTCCTTAGCTCAGTTGGTAGAGCGTGAGACTTTTAATCTTAAGGTCAGGGGTTCGAGTCCCCTAGGTGGCTCCA	II	6565216	6565291	1	5
Lys	TTT	GTGTCCTTAGCTCAGTTGGTAGAGCGTGAGACTTTTAATCTTAAGGTCAGGGGTTCGAGTCCCCTAGGTGGCTCCA	I	8953162	8953237	1	5
Ile	TAT	GCCCCATTGGCGCAGTCGGTTAGCGCGTGGTACTTATAATGCCAAGGTCGCCAGTTCGAGCCTGGCATGGGGCACCA	V	6917448	6917485	1	5
Met	CAT	GCTTCCGTAGCGCAGTAGGCAGCGCGTCAGTCTCATAATCTGAAGGTCGTGAGTTCGAGCCTCACCGGGAGCACCA	I	3638852	3638927	-1	5
Trp	CCA	GACTGCTTGGCGCAATGATAGCGCGTTCGACTCCAGATCGAAAGGTTGGGCGTTCGATCCGCTCAGTGGTCACCA	II	12539533	12539607	1	6

Trp	CCA	GACTGCTTGGCGCAATGGTAGCGCGTTCGACTCCAGATCGAAAGGTTGGGCGTTCGATCCGCTCAGTGGTCACCA	I	946044	946118	-1	6
Trp	CCA	GACTGCTTGGCGCAATGGTAGCGCGTTCGACTCCAGATCGAAAGATTGGGCGTTCGATCCGCTCAGTGGTCACCA	X	14228848	14228922	-1	6
Asn	GTT	GCTTTACCTGTGGGCGCAATAGGCAGCGCGTTCGGCTGTTAACCGAAAGGTTGGTGGTTCGAGCCCACCCGAGAGCGCCA	I	7718875	7718950	-1	7
Asn	GTT	GCTTCCGTGGCGCAATAGGCAGCGCGTTCGGCTGTTAACCGAAAGGTTGGTGGTTCGAGCCCACCCGGGAGCGCCA	I	9051243	9051318	-1	7
Asn	GTT	GCTTCCGTGGCGCAATAGGCAGCGCGTTCGGCTGTTAACCGAAAGGTTGGTGATTCGAGCCCACCCGGGAGCGCCA	X	7773139	7773214	1	7
Arg	TCG	GGTCGCGTCGCCTAATGGATAAGCCACCAGACTTCGAATCTGGGGATTACAGGTTCGATCCCTGCCGTGGTCGCCA	X	11806517	11806592	-1	8
Arg	TCG	GGCCGCGTGGCCTAATGGATAAGGCACCAGACTTCGAATCTGGGGATTGCAGGTTCGAGTCCTGCCGTGGTCGCCA	I	9267115	9267190	1	8
Arg	TCG	GGCCGCGTGGCCTAATGGATAAGGCATCAGACTTCGAATCTATGGGGATTGCAGGTTCGATCCCTGCCGTGGTCGCCA	X	1089397	1089474	1	8
Arg	TCG	GGCCGCGTGGCCTAATGGATAAGGCATCAGACTTCGAATCTGGGGATTGCAGGTTCGATCCCTGCCGTGGTCGCCA	X	1089835	1089910	1	8
Arg	TCG	GGCCGCGTGGCTAATGGATAAGGCATCAGACTTCGAATCTGGGGATTGCAGGTTCGATCCCTGCCGTGGTCGCCA	X	16461404	16461479	1	8
Arg	CCT	ACCCGTGTAGCCTAAATGGATAAGGCATCGGTCTCCTAAACCAAAGGATGCGGGTTCGAGTCCTGCCACGGGTGCCA	X	1764246	1764322	-1	9
Arg	CCG	GCTCGCGTGGCCTAATGGATAAGGCACCGGACTCCGGAACCGGGAATGGGGTTCAAGTCCCTCCGCGAGCTCCA	II	12728682	12728756	-1	9
Arg	CCG	GCCCGCGTGGCCTAATGGATAAGGCACCGGACTCCGGAACCGGGAATGGGGTTCGAGTCCCCCCGCGAGCTCCA	X	1629873	1629947	-1	9
Arg	ACG	GGCCGCGTGGGGCAATGGATAACGCGTCTGCCTACGGAGCAGAAGATTGTAGGTTCGAATCCTGCCGTGGTCGCCA	X	8623551	8623626	1	10
Arg	ACG	GGCCGCGTGGCGCAATGGATAACGCGTCTGCCTACGGAGCAGAAGATTGCAGGTTCGAATCCTGCCGTGGTCGCCA	I	5843083	5843158	-1	10
Arg	ACG	GGCCGTGGCGCAATGGATAACGCGTCTGCCTACGGAGCAGAAGATTGTAGGTTCGAATCCTGCCGTGGTCGCCA	X	8639170	8639245	-1	10
Arg	ACG	GGCCGCGTGGCGCAATGGATAACGCGTCTGCCTACGGAGCAGAAGATTGTAGGTTCGAATCCTGCCGTGGTCGCCA	X	10132619	10132694	1	10
Arg	ACG	GGCCGCGTGGCGCAATGGATAACGCGTCTGCCTACGGAGCAGAAGATTGTAGGTTCGAATCCTACCGTGGTCGCCA	X	10018364	10018439	-1	10
Arg	CCT	GGCCGTGTGGCCTAATGGATAAGGCGTCGGTCTCCTAAACCGAAGACTGCAGGTTCGAGTCCTGCCTCGGTCGCCA	IV	143982	144057	1	11
Arg	TCT	GGCCTTGTGGCCTAATGGATAAGGCGTCTGACTTCTAATCAGAAGATTGCAGGTTCGAGCCCTGCCTGGGTCACCA	I	1447140	1447215	1	11
Arg	TCT	GGCCTTGTGGCCTAATGGATAAGGCGTCTGACTTCTAATCAGAAGATTGCAGGTTCGACCCCTGCCTGGGTCACCA	X	15146489	15146564	-1	11
Arg	TCT	GGCCTTGTGGCCTAATGGATAAGGCGTCTGACTTCTAATCAGAAGATTGCAGGTTCGATCCCTGCCTGGGTCACCA	I	12198594	12198669	1	11
Ser	CGA	GTGGTTAAGAATGTCCGAGTGGTTAAGGAGTTTGACTCGAAATCAAATGGGCTCTGTCCGCGTAGGTTCGAATCCTGCTGACTGCGCCA	III	6158917	6159001	1	12
Ser	AGA	GCCGTCATGTCCGAGTGGTTAAGGAGATTGACTAGAAATCAATTGGGCTCTGCCCGCGTAGGTTCGAATCCTGCTGACTGCGCCA	X	15769938	15770022	-1	12
Ser	AGA	GCAGTCATGTCCGAGTGGTTAAGGAGATTGACTAGAAATCAATTGGGCTCTGTCCGCGTAGGTTCGAATCCTGCTGACTGCGCCA	X	15773459	15773543	1	12
Ser	AGA	GCAGTCATGTCCGAGTGGTTAAGGAGATTGACTAGAAATCAATTGGGCTCTGCCCGCGTAGGTTCGAATCCTGCTGACTGCGCCA	II	1520042	1520126	1	12
Ser	AGA	GCAGTCATGTCCGAGTGGTTAAGGAGATTGACTAGAAATCAATTGGGCTTTGCCCGCGTAGGTTCGAATCCTGCTGACTGCGCCA	III	9861932	9862016	-1	12
Ser	CGA	GCAGTCATGTCCGAGTGGTTAAGGAGTTTGACTCGAAATCAAATGGGCTCTGCCCGCGTAGGTTCGAATCCTGCTGACTGCGCCA	I	11803304	11803388	-1	12
Ser	CGA	GCAGTCATGTCCGAGTGGTTAAGGAGATTGACTCGAAATCAATTGGGCTCTGCCCGCGTAGGTTCGAATCCTGCTGACTGCGCCA	III	6513881	6513965	-1	12
Ser	TGA	GCAACGATGTCCGAGTGGTTAAGGAGATGGACTTGAAATCCATTGGGCTTTGCCCGCGTAGGTTCGATTCCTGCTCGTTGCGCCA	X	12889870	12889954	1	13
Ser	TGA	GCTGCGATGTCCGAGCGGTTAAGGAGTTGGACTTGAAATCCAATGGGCATTGCCCGCGTAGGTTCAAATCCTGCTCGCAGCGCCA	IV	16577273	16577357	1	13
Ser	TGA	GCTGCGATGTCCGAGTGGTTAAGGAGTTGGACTTGAAATCCAATGGGCATTGCCCGCGTAGGTTCAAATCCTGCTCGCAGCGCCA	X	10124439	10124523	1	13
Ser	TGA	GCAGCGATGTCCGAGTGGTTAAGGAGTTGGACTTGAAATCCAATGGGCTTTGCCCGCGTAGGTTCGAACCCTGCTCGCTGCGCCA	I	9605748	9605832	1	13
Ser	TGA	GCTGCGATGCCCGAGTGGTTAAGGAGTTGGACTTGAAATCCAATGGGCATTGCCCGCATAGGTTCGAACCCTGCTTGCAGCGCCA	V	15550593	15550677	1	13
Ser	TGA	GCTGCGATGTCCGAGTGGTTAAGGAGTTGGACTTGAAATCCAATGGGCATTGCCCGCGTAGGTTCGAACCCTGCTCGCAGCGCCA	V	12780366	12780450	-1	13
Ser	TGA	GCTGCGATGTCCGAGTGGTTAAGGAGTTGGACTTGAAATCCAATGGGCATTGCCCGCGTAGGTTCGAACCCTGCTTGCAGCGCCA	V	15547853	15547937	-1	13

Leu	AAG	GGAGAGATGGCCGAGCGGTCCAAGGCGCTGGTTTAAGGCACCGTTCCATTCGGGGGCGTGGGTTCGAATCCCACTCTCTTAACCA	III	5769859	5769943	1	14
Leu	AAG	GGAGAGATGCCCGAGCTGTCTAAGGCGCTGATTTAAGGCACCAGCCTTTTCGGGGGCGTGAGTTCGAATCCCACTCTCTTCTCCA	X	8409240	8409324	-1	14
Leu	AAG	GGTGAGATGGCCGAGCGGTCTAAGGCGCTGGTTTAAGGCACCAGTCCCTTCGGGGGCGTGGGTTCGAATCCCACTCTCATCACCA	II	14635257	14635341	-1	14
Leu	TAG	GGTGAGATGGCCGAGTGGTCTAAGGCGCTGGTTTTAGGCACCAGTCCCTCCGGGGGCGTGGGTTCGAATCCCACTCTCATCACCA	II	6853161	6853245	1	14
Leu	TAA	GGAGAGATGGCCGAGCGGTCCAAGGCGCTGGTTTAAGGCAACCAGTAGCTTCGGGGGCGTGGGTTCGAATCCCACTCTCTTCACCA	II	6721729	6721814	1	14
Leu	AAG	GGAGAGATGGCCGAGCGGTCTAAGGCGCTGGTTTAAGGCACCAGTCCCTTCGGGGGCGTGGGTTCGAATCCCACTCTCTTCACCA	X	8939496	8939580	1	14
Leu	AAG	GGAGAGATGGCCGAGCGGTCCAAGGCGCTGGTTTAAGGCACCAGTCCCTTCGGGGGCGTGGGTTCGAATCCCACTCTCTTCACCA	I	11585358	11585442	-1	14
Leu	CAG	GCTGTTTTGCATGGCCGAGTGGTCTAATTAGAGCCTTAAGGCGCTGCGTTCAGGTCGCAGTCCTCTCAGGAGGGCGCAGGTTCAAATCCTGCGGACAGCACCA	V	11183645	11183731	-1	15
Leu	CAG	GCCGTTCTGGCCGAGTGGTCTAAGGCGCTGCGTTCAGGTCGCAGTCCTCTCCGGAGGGCGCAGGTTCGAATCCTGCGGACGGCACCA	I	754683	754769	1	15
Leu	CAG	GCCGTTCTGGCCGAGTGGTCTAAGGCGCTGCGTTCAGGTCGCAGTCCTCTCAGGAGGGCGCAGGTTCGAACCCTGCGGACGGCACCA	IV	6556807	6556893	1	15
Gly	CCC	GCGGTGGTGGCCGAGCGGTCAAGGCGTAGGACTCCCGATCCTATTCTGGTAACAGAGCGCGGGTTCGAATCCCGTCCACCGCACCA	I	10601840	10601925	1	16
Gln	TTG	GCCCCGGTGGCCGAGCGGTCGAAGGCGTGAGACTTGTTCTCATTGGGTTAAACCAGTCGCGGGTTCGAATCCCGCCCGGGGCACCA	I	9558784	9558869	1	16
Ile	TAT	GCCCCGGTGGCCGAGCGGTCGAAGGCGTGAGACTTATGATCTCATTGGGTTAAACCAGTCGCGGGTTCGAATCCCGCCCGGGGCACCA	I	7960832	7960919	1	16
Lys	CTT	GACACGGTGGCCGAGTGGTTTAAGGCATGAGACACTTGATCTCAAACGGTTCTAACCGAACGCAGGTTCGAATCCTGCCCGTGTCACCA	III	6724077	6724165	-1	16
Arg	CCT	GCCACGGTGGCCGAGTGTGGTCAAAGGCGTGAGACTCCTGATCTCTTTCGGGCAACCGATCGCAGGTTCGAATCCTGCCCGTGGCACCA	III	6726933	6727021	-1	16
His	GTG	GCCACGGTGGCCGAGTGGTCAAGGCGTGAGCTTGTGGGATGCGCTCATGGGGTTAAACCCATCGCAGGTTCGAATCCTGCCCGTGGCACCA	IV	11721126	11721216	1	16
Ser	GCT	GATCAGGTGGCCGAGTGGTTAAGGCGATGGACTGCTAATCCATTGGGGTTTCCCCGCGTCAAATCTCATCCTGATCGAACATCCA	III	13009322	13009406	1	17
Ser	GCT	GATCAGGTGGCCGAGTGGTTAAGGCGATGGACTGCTAATCCATTGGGGTTTCCCCGCGTGAGTTCGAATCTCATCCTGATCGCCA	I	14162836	14162920	1	17
Ser	GCT	GATCAGGTGGCCGAGTGGTTAAGGCGATGGACTGCTAATCCATTGGGGTTTCCCCGCGTGAGTTCGAATCTCATCTTGATCGCCA	X	13442575	13442659	1	17
Leu	CAA	GCACGGATGGCCGAGTGGTCTAAGGCGCCAGACTCAAGTTCTGGTACTCGTATGGGTGCGTGGGTTCGAATCCCACTTCGTGCACCA	III	4362496	4362533	1	17
Leu	CAA	GCACGGATGGCCGAGTGGTCTAAGGCGCCAGACTCAAGTTCTGGTACTCGTACGGGTGCGTGGGTTCGAATCCCACTTCGTGCACCA	X	13443315	13443363	-1	17
Leu	TAA	AGCACGATGGCCGAGTGGTTAAGGCGTTGGACTTAAGTTCCAATGGTGGATAACACCTCGTGGGTTCGAACCCCACTCGTGCTACCA	II	5772893	5772979	-1	17
Leu	TAA	AGCACGATGGCCGAGTGGTTAAGGCGTTGGCCTTAAGTTCCAATGGTGGATAACACCGCGTGGGTTCGAACCCCACTCGTGCTACCA	II	8631904	8631990	-1	17
Met	CAT	AGCAGCGTGGCGCAGTGGAAGCGTGCTGGGCCCATAACCCAGAGGTCGGTGGATCGAAACCACTCGCTGCTACCA	I	12142902	12142976	1	18
Met	CAT	ATCAACTGTGGCAGTGGAAGCGTGCTGGGCCCATAACCCAGAGGTCGGTGGATCGAAACCACTCGCTGCTACCA	IV	66981	67055	1	18
SeC	TCA	GCCCGGATGAACCATGGCGGTCTGTGGTGCAGACTTCAAATCTGTAGGCGGTTAGCGCCGCAGTGGTTCGACTCCACCTTTCGGGTCCA	IV	658181	658269	1	19
Arg	GCG	TGGCAGTGGCTCAACTGGGTAGAGCTTTGCCTGCGACGCATAAGACCAGGGTTCGAGTCCCGCTGTAGCCA	III	10961748	10961818	-1	19
Gly	GCC	TGCAAGTGGCTCAATCGGTTAGAGAGATGGTTGCCACCCACAAGTCCGGGGTTCGAACCCCGACTGTGGCCA	V	17931052	17931123	1	19
His	ATG	CGCTGTGGCTCAAGTGGGAAGAGGGATGGCTATGGTGCAAAAGGTCACGGGTTCGAACACCGGGTAGTGGCCA	V	20219270	20219342	1	19
Ile	TAT	GTCGCAGTGGCTCAGGTGGGTAGAGTAATGACTATGGGGAAATAGGTCCGGGGTTCGAGCCCCCGCTGATGGCACCA	V	18373932	18374008	-1	19
Thr	TGT	GAGTTTTGGCTCGACTGGTAAGAGGTGTGACTTGTGATCAATAGGTCCGGGGTTCGACCCCTCGTAAGGGTCACCA	II	1102364	1102439	-1	19
Asp	GTC	TCCTCGGTAGTATAGTGGTGAGTATCCGCGTCTGTCACATGCGAGACCCGGGTTCAATTCCCGGCCGGGAGACCA	X	8155703	8155777	1	20
Asp	GTC	TCCTCGGTAGTATAGTGGTGAGTATCCGCGTCTGTCACATGCGAGACCCGGGTTCAATTCCCGGCCGGGGAGCCA	I	10807193	10807267	1	20
Asp	GTC	TCCTCGGTAGTATAGTGGTGAGTATCCGCGTCTGTCACATGCGAGACCCGGGTTCAATTCCCGGCCGGTGAGCCA	IV	2808353	2808427	1	20
Asp	GTC	TCCTCGGTAGTATAGTGGTGAGTATCCGCGTCTGTCACATGCGAGGCCCGGGTTCAATTCCCGGCCGGGGAGCCA	IV	2800097	2800171	1	20

Asp	GTC	TCCTCGGTAGTATAGTGGTGAGTATCCGCGTCTGTCACATGCGAGACCCTGGTTCAATTCCCGGCCGGGGAGCCA	X	16617816	16617890	-1	20
Asp	GTC	TCCTCGGTAGTATAGTGGTGAGTATCCGCGTCTGTCGCATGCGAGACCCGGGTTCAATTCCCGGCCGGGGAGCCA	IV	2802335	2802409	1	20
Asp	GTC	TCCTCGGTAGTGTAGTGGTGAGTATCCGCGTCTGTCACATGCGAGACCCGGGTTCAATTCCCGGCCGGGGAGCCA	IV	2805625	2805699	1	20
Gly	GCC	GCATTGGTGGCTCAGTGGTAGAATGCTCGCCTGCCACGCGGGCAGCCCGGGTCCAATTCCCGGTCGATGCACCA	I	11158619	11158692	1	21
Gly	GCC	GCATCGGTGGTTCAGTGGTAGAAAGCTCGCCTGCCACGCGGGCGGCCCGGGTTCGATTCCCGGTCGATACACCA	II	5782189	5782262	-1	21
Gly	GCC	GCATCGGTGGTTCAGTGGTAGAATGCTCGCCTGCCACGCGTGCGGCCCGGGTTCGATTCCCGGTCGATGCACCA	V	4310596	4310669	1	21
Gly	GCC	GCATCGGTGGTTCAGTGGTAGAATGCTCGCCTGCCACGCGGGCGGCCCGGGTTCGATTCCCGGTCGATGCACCA	I	10593126	10593199	1	21
Gly	GCC	GCATCGGTGGTTCAGTGGTAGAATGCTCGCCTGCCACGCGGCGGCCCGGGTTCGATTCCCGGTCGATGCACCA	III	5762007	5762079	-1	21
His	GTG	GCCTGCGTAGTATAGTGGTTAGTACTCCACGTTGTGGCCGTGGCGACGCTGGTTCGATTCCAGCCGCAGGCACCA	II	3521487	3521561	-1	22
His	GTG	GCCCTCTTAGTATAGTGGCTAGTACTCCACGTTGTGGTCGTGGCAACGCGGGTTCGATTCCAGCAGCAGGCACCA	IV	13870240	13870314	1	22
His	GTG	TACTACTATACAGTGGTTAGTACTCCACGTTGTGGCCGTGGCGACGCTGGTTCGATTCCAGCAGCAGGCACCA	IV	4861744	4861818	1	22
His	GTG	GCCTGCTTAGTATAGTGGTTAGTACTCCACGTTGTGGCCGTGGCGACGCTGGTTCGATTCCAGCAGCAGGCACCA	IV	15187619	15187693	-1	22
His	GTG	GCCTGCTTAGTATAGTGGTTAGTACTCCACGTTGTGGCCGTGGCGACGCTGGTTAGATTCCAGCAGCAGGCACCA	IV	15187312	15187386	1	22
Glu	TTC	TCCTATGTGGTCTAGTGGTTAGGATTCGTGGTTTTCACCCACGCGGCCCGGTTTCGATTCCCGGCATGGGAACCA	IV	2639804	2639878	-1	23
Glu	TTC	TCCCATGTGGTCTAGTGGTTAGGATTCGTGGTTTTCACCCACGCGGCCCGGGTTCGATTCCCGGCATGGGAACCA	I	9883687	9883761	1	23
Glu	TTC	TCCCATGTGGTCTAGTGGTTAGGATTCGTGGTTTTCACCCACGCGGCCCGGTTCGATTCCCGGCATGGGAACCA	V	7069726	7069800	-1	23
Glu	CTC	TCCGTTGTTGTCTAGTGGTTAGGATTTATGGCTCTCACCCATAAGGCCGGGGTTCGATTCCCCGCAACAAATCCA	X	16274425	16274499	1	23
Glu	CTC	TCCGTTGTGGTCTAGTGGTTAGGATTTATGGCTCTCACTCATAAGGCCGGGGTTCGATTCCCCGCAACGGAACCA	III	7642090	7642164	-1	23
Glu	CTC	TCCGTTGTGGTCTAGTAGTTAGGATTTATGGCTCTCACCCATAAGGCCGGGGTTCGATTCCCCGCAACGGAACCA	X	16273694	16273768	1	23
Glu	CTC	TCCGTTGTGGTCTAGTGGTTAGGATTTATGGCTCTCACCCATAAGGCCGGGGTTCGATTCCCCGCAACGGAACCA	I	11977983	11978057	1	23
Glu	CTC	TCCGTTGTGGTCTAGTGGTTAGGATTTATGGCTCTCACCCATAAGGCCGGGGTTCGATTCCCCGCAACGAAACCA	X	7906537	7906611	1	23
Gly	TCC	GCGGTTTACCTGTGATGGTCAGCATGGATGCCTTCCAAGCATTCGACGGGGGTTCGATTCCCCCCGAACGCACCA	X	14010184	14010258	1	24
Gly	TCC	GCGTTCGTGGTGTAATGGTCAGCATGGATGCCTTCCAAGCATTCGACCGGGGTTCGATTCCCCCCGAAGGCACCA	IV	16684347	16684421	-1	24
Gly	TCC	GCGTTCGTGGTGTAATGGTCAGCATGGATGCCTTCCAAGCATTCGACGGGGGTTCGATTCCCCTCGAACGCACCA	X	8846668	8846742	1	24
Gly	TCC	GCGTTCGTGGTGTAATGGTCGGCATGGATGCCTTCCAAGCATTCGACGGGGGTTCGATTCCCCCCCCCCGAACGCACCA	X	3994581	3994655	1	24
Gly	TCC	GCGTTCGGGGTGTAATGGTCAGCATGGATGCCTTCCAAGCATTCGACGGGGGTTCGATTCCCCCCGAACGCACCA	I	13324984	13325058	1	24
Gly	TCC	GAGTTCGTGGTGTAATGGTCAGCATGGATGCCTTCCAAGCATTCGACGGGGGTTCGATTCCCCCCGAACGCACCA	X	8819628	8819702	1	24
Gly	TCC	GCGTTCGTGGTGTAATGGTCAGCATGGATGCCTTCCAAGCATTCGACGGGGGTTCGATTCCCCCCGAACGCACCA	I	9320170	9320244	1	24
Gly	TCC	GCGTTCGTGGTGTAATGGTCAGCATGGATGCCTTCCAAGCATTCGACGGGGGTTCGATTCCCCCCCGAACGCACCA	X	8846450	8846524	-1	24
Gln	CTG	GGTTCCATGGTGTAGCGGTTAGCACTCAGTACTCTGAATCCTGCGACCCGAGTTCAAATCTCGGTGGGACCTCCA	X	11872438	11872512	1	25
Gln	CTG	GGTTCCATGGTGTAGCGGTTAGCACTCAGGACTCTGAATCCTGCGACCCGAGTTCAAATCTCGGTGGGACCTCCA	IV	5334704	5334778	1	25
Gln	CTG	GGTTCCATGGTGTAGTGGTTAGCACTCAGGACTCTGAATCCTGCGACCCGAGTTCAAATCTCGGTGGGACCTCCA	V	14231349	14231423	1	25
Gln	TTG	GGTTCCATGGTGTAGCGGTTAGCACTCATGACTTTGAATCCTGCGACCCGAGTTCAAATCTCGGTGGAACCTCCA	X	8925588	8925662	1	25
Gln	TTG	GGTTCCATGGTGTAGCGGTTAGCACTCAGGACTTTGAATCCTGCGATCCAAGTTCAAATCTCGGTGGAACCTCCA	X	9074614	9074688	-1	25
Gln	TTG	GGTTCCATGGTGTAGCGGTTAGCACTCAGGACTTTGAATCCTGCGACCCGAGTTCAAATCTCGGTGGAACCTCCA	II	14474764	14474838	-1	25
Gln	TTG	GGTTCCATGGTGTAGCGGTTAGCACTCAGGACTTTGAATCCTGCGACCCGAGTTCAAATCTCAGTGGAACCTCCA	X	9091254	9091328	1	25

Val	AAC	GGTTTCGTGGTGTAGTGGTTATCACATCTGTCTAACACACAGAAGGTCGGTGGTTCGAGCCCGCCCGAGATCACCA	IV	15235356	15235431	1	26
Val	AAC	GGTTTCGTGGTGTAGTGGTTATCACATCTGTCTAACACACAGAAGGTCGGTGGTTCGAGCCCGCCCGAGATCTCCA	X	17611213	17611288	1	26
Val	AAC	GGTCTCGTGGTGTAGTGGTTATCACATCTGTCTAACACACAGAAGGTCGGTGGTTCGAGCCCGCCCGAGATCACCA	I	12065856	12065931	1	26
Val	AAC	GGTCTCGTGGTGTAGTGGTTATCACATCTGTCTAACACACAGAAGGTCGGCGGTTCGATCCCGCCCGAGATCACCA	X	16298269	16298344	1	26
Val	CAC	GGTCCTGTGGTGTAGTGGTTATCACGTCTGCTTCACACGCAGAAGGTCGCCGGTTCGAACCCGGCCAGGACCTCCA	IV	6481150	6481225	1	27
Val	CAC	GGTCCTCTGGTGTAGTGGTTATCACGTCTGCTTCACACGCAGAAGGTCGCCGGTTCGAACCCGGCGAGGACCTCCA	IV	620482	620557	1	27
Val	TAC	GGTCCTGTGGTGTAGTGGTTATCACGTCTGCTTTACACGCAGTAGGCCGCCGGTTCGATCCCGGCCAGGACCTCCA	II	11959312	11959387	1	27
Val	TAC	GGTCCTGTGGTGTAGTGGTTATCACGTCTGCTTTACACGCAGAAGATCGCCGGTTCGAACCCGGCCAGGACCTCCA	V	12683331	12683406	-1	27
Val	TAC	GGTCCTGTGGTGTAGTGGTTATCACGTCTGCTTTACACGCAGAAGATCGCCGGTTCGATCCCGGCCAGGACCTCCA	X	3793545	3793620	-1	27
Val	TAC	GGTCCTATGGTGTAGTGGTTATCACGTCTGCTTTACACGCAGAAGATCGCCGGTTCGAACCCGGCTAGGACCTCCA	II	5577180	5577255	-1	27
Met	CAT	GGTCCTGTAGTGGTTATCACGTCTGCTTCATACACAGAAGGTCGCCGGTTCGAACCCGGCCAGGACCTCCA	V	15517378	15517453	1	27
Pro	AGG	GGTCGGATGGCCTAGAGGTAAGGCGCTTGCTTAGGGTGCAAGAGATCCCGGGTTCGATCCCCGGTTCGACCCCCA	X	8037458	8037532	1	28
Pro	TGG	CATGGTCTAGTGGTATGATTCTCGCTTTGGGTGCGAGATGTCCCGGGTTCAATCCCCGGTTCGGCCA	X	15747732	15747806	-1	28
Pro	TGG	GGCCGAATGGTCTAGTGGTTTGATTCTCGCTTTGGGTGCGACAAGTCCCGGTTCAATCCCCGGTTCGGCCCCCA	X	15747963	15748037	1	28
Pro	TGG	GGCCGAATGGTCTATTGGTATGATTCTCGCTTTGGGTGCGAGAGGTCCCGGGTTCAATCCCCGGTTCGGCCCCCA	X	12854767	12854841	-1	28
Pro	TGG	GGCCGAATGGTCTAGTGGTATGATTCTCGCTTTGGGTGCGAGAGGTCCCGGGTTCAATCCCCGGCTCGGCCCCCA	II	10302096	10302170	-1	28
Pro	TGG	GGCCGAATGGTCTAGTGGTATGATTCTCGCTTTGGGTGCGAGAGGTCCCGGGTTCAATCCCCAGTTCGGCCCCCA	V	3582125	3582199	-1	28
Pro	TGG	GGCCGAATGGTCTAGTGGTATGATTCTCGCTTTGGGTGCGAGAGGTCCCGCGTTCAATCCCCGGTTCGGCCCCCA	V	3588298	3588372	-1	28
Pro	TGG	GGCCGAATGGTCTAGTGGTATGATTCTCGCTTTGGGTGCGAGAGGTCCCTGGTTCAATCCCCGGTTCGGCCCCCA	II	10304708	10304782	-1	28
Pro	TGG	GGCCGAATGGTCTAGTGGTATGATTCTCGCTTTGGGTGCGAGAGGTCCCGGGTTCAATCCCCGGTTCGGCCCCCA	II	10302339	10302413	1	28
Pro	TGG	GGCCGAATGGTCTAGTGGTATGATTCTCGCTTTGGGTGCGAGAGGTCCCGGGTTGAATCCCCGGTTCGGCCCCCA	III	8069340	8069414	1	28
Pro	CGG	GGCCGGATGGTCTAGAGGTATGATTCTCGCTTCGGGTGCGAGAGGTCCCGGGTTCGATTCCCGGTTCGGCCCCCA	III	8650298	8650372	-1	28
Pro	AGG	GGCTGAGTGGTCTAGTGGTATGATTCTCGCTTAGGGTGCGAGAGGTCCCGGGATCGATCCCCGGCTCAGCCCCCA	I	9268918	9268992	-1	28
Pro	AGG	GGCCGGATGGTCTAGTGGTATGATTCTCGCTTAGGGTGCGAGAGGTCCCGGGATCGATCCCCGGTCCGGCCCCCA	X	3970076	3970150	1	28
Pro	AGG	GGCCGGATGGTCTAGTGGTATGATTCTCGCTTAGGGTGCGAGAGGTCCCGGGATCGATCCCCGGTTCGGCCCCCA	X	3970245	3970319	1	28
Cys	GCA	GGGGGTATAGCTCAGTGGCAGAGCATTCGACTGCAGATCGAGAGGTCCCTGGTTCAACTCCGGGTGCCCCCTCCA	I	6781804	6781878	1	29
Cys	GCA	TGGGGTATAGCTCAGTGGCAGAGCATTCGATTGCAGATCGAGAGGTCCCTGGTTCAACTCCGGGTGCCCCCTCCA	V	6335381	6335455	1	29
Ala	AGC	GGGGGTATAGCTCAGTGGTAGAGCGCTCCCTTAGCATGGGAGAGGGCTGGGGTTCAATTCCCATTTTCCGTTCCA	II	4565010	4565084	-1	30
Ala	AGC	GGGGGTATAACTCAGTGGTAGATCGCTCCCTTAGCATGGGAGAGGGCTGGGGTTCAATTCCCCAAACCTCCACCA	V	857326	857400	1	30
Ala	AGC	GTGTAGCTCAGTGGTAGAGCGCTCCCTTAGCATGGGAGAGGGCTGGGGTTCAATTCCCCATAACTCCACCA	I	12601764	12601838	1	30
Ala	AGC	GGGGGTATAGCTCAGTAGTAGAGCGCTCCCTTAGCATGGGAGAGGGCTGGGGTTCAATTCCCCATACCTCCACCA	IV	6562191	6562265	1	30
Thr	AGT	GGGGGTATAGCTCAGTGGTAGAGCGCTCCCTTAGTATGGGAGAGGGCTGGGGTTCAATTCCCCATACCTCCACCA	II	7003231	7003305	1	30
Ala	AGC	GGGGGTATAGCTCAGTGGTAGAGCGCTCCCTTAGCATGGGAGAGGGCTGGGGTTCAATTCCCCATACCTCCACCA	II	4565386	4565460	1	30
Ala	AGC	CGGGGTATAGCTCAGTGGTAGAGCGCTCCCTTAGCATGGGAGAGGGCTGGGGTTCAATTCCCCATACCTCCACCA	III	4428922	4428996	-1	30
Ala	TGC	GGGGGTATAGCTCAGGGGTAGAGCGCTCGCTTTGCATGCGAGAAGTCTGGGGTTCGATTCCCCATACCTCCACCA	II	11334444	11334518	1	30
Ala	CGC	GGGGGTATAGCTCAGGGGTAGAGCGCTCGCTTCGCATGTGAGAAGTCTGGGGTTCAATTCCCCATACCTCCACCA	V	9318178	9318252	1	30

Ala	CGC	GGGGGTATAGCTCAGGGGTAGAGCGCTCGCTTCGCATGCGAGAAGTCTGGGGTTCAATTCCCCATACCTCCACCA	V	9503538	9503612	1	30
Ala	CGC	GGGGGCATAGCTCAGAGGTAGAGCGCCCGCTTCGCATGCGGGAAGTCCGGGGTTCAATCCCCCGTGCCTCCACCA	V	15479964	15480038	-1	30
Ala	CGC	GGGGGCATAGCTCAGGGGTAGAGCGCTCGCTTCGCATGCGAGAAGTCCGGGGTTCAATTCCCCGTGCCTCCACCA	X	12890212	12890286	-1	30

Supplementary Table S4. tRNA gene copy number per anticodon

3rd base	2nd base																1st base
	A				G				T				C				
	A.A.	A.C.	Unique copy	All copy	A.A.	A.C.	Unique copy	All copy	A.A.	A.C.	Unique copy	All copy	A.A.	A.C.	Unique copy	All copy	
A	Phe	AAA	no gene		Ser	AGA	4	15	Tyr	ATA	Pseudo		Cys	ACA	no gene		A
		GAA	4	14		GGA	no gene			GTA	7	19		GCA	2	13	G
	Leu	TAA	3	4		TGA	7	9	Stop	TTA	Pseudo		SeC(e)	TCA	1	1	T
		CAA	2	7		CGA	3	6		CTA	Pseudo		Trp	CCA	3	12	C
G		AAG	5	19	Pro	AGG	4	6	His	ATG	1	1	Arg	ACG	5	19	A
		GAG	Pseudo			GGG	Pseudo			GTG	6	19		GCG	1	1	G
		TAG	1	3		TGG	9	32	Gln	TTG	5	20		TCG	5	10	T
		CAG	3	6		CGG	1	4		CTG	3	7		CCG	2	2	C
T	Ile	AAT	2	21	Thr	AGT	3	17	Asn	ATT	no gene		Ser	ACT	no gene		A
		GAT	Pseudo			GGT	Pseudo			GTT	3	20		GCT	3	9	G
		TAT	3	8		TGT	2	10	Lys	TTT	2	15	Arg	TCT	3	8	T
	Met	CAT	4	20		CGT	5	7		CTT	7	31		CCT	3	4	C
C	Val	AAC	4	19	Ala	AGC	6	22	Asp	ATC	Pseudo		Gly	ACC	Pseudo		A
		GAC	Pseudo			GGC	Pseudo			GTC	7	27		GCC	6	16	G
		TAC	4	5		TGC	1	8	Glu	TTC	3	17		TCC	8	36	T
		CAC	2	6		CGC	4	4		CTC	5	24		CCC	1	3	C

Supplementary Table S5. Alignment of abundant tRFs (top 1%)

tRF group for alignment	tRF sequence alignment	Count sequence read				True positive?
		Day 0	Day 5	Day 8	Day 12	
1	[GGCCGAATGGTCTAGTGGTATGATTCTCGCTTTG]	11	9	256	50	yes
1	[GGCCGAATGGTCTAGTGGTATGATTCTCGCT---]	3	12	160	23	no
1	[GGCCGAATGGTCTAGTGGTATGATTCTCGC----]	1	8	55	20	no
1	[GGCCGAATGGTCTAGTGGTATGATTCTC------]	7	19	77	42	no
1	[GGCCGAATGGTCTAGTGGTATGATTCT-------]	22	25	47	368	no
1	[GGCCGAATGGTCTAGTGGTAT-------------]	10	81	70	67	no
1	[GGCCGAATGGTCTAGTGG----------------]	9	95	72	60	no
2	[TCCTCGGTAGTATAGTGGTGAGTATCCGCGTCTG]	10	13	409	135	yes
2	[TCCTCGGTAGTATAGTGGTGAGTATCCGCGT---]	5	8	161	61	no
2	[TCCTCGGTAGTATAGTGGTGAGTATCCGCG----]	43	36	477	162	no
2	[TCCTCGGTAGTATAGTGGTGAGTATCCGC-----]	9	23	111	96	no
2	[--CTCGGTAGTATAGTGGTGAGTATCCGC-----]	4	5	5	164	no
2	[TCCTCGGTAGTATAGTGGTGAGTATCC-------]	20	6	4	231	no
2	[----CGGTAGTATAGTGGTGAGTATCCGCG----]	38	37	5	103	no
2	[--CTCGGTAGTATAGTGGTGAGTATCCG------]	4	4	9	138	no
2	[TCCTCGGTAGTATAGTGGTGAGTATC--------]	23	15	6	92	no
2	[---TCGGTAGTATAGTGGTGAGTATCCGC-----]	1	4	6	135	no
2	[-CCTCGGTAGTATAGTGGTGAGTATC--------]	37	97	14	86	no
2	[--CTCGGTAGTATAGTGGTGAGTATCC-------]	14	52	66	232	no
2	[TCCTCGGTAGTATAGTGGTGAGTAT---------]	64	29	26	230	no
2	[---TCGGTAGTATAGTGGTGAGTATCCG------]	3	4	11	103	no
2	[-CCTCGGTAGTATAGTGGTGAGTAT---------]	43	127	47	95	no
2	[--CTCGGTAGTATAGTGGTGAGTATC--------]	7	57	101	134	no
2	[TCCTCGGTAGTATAGTGGTGAGTA----------]	36	34	40	187	no
2	[---TCGGTAGTATAGTGGTGAGTATCC-------]	5	17	87	190	no
2	[--CTCGGTAGTATAGTGGTGAGTAT---------]	4	108	232	221	no
2	[TCCTCGGTAGTATAGTGGTGAGT-----------]	81	45	76	210	no
2	[--CTCGGTAGTATAGTGGTGAGTA----------]	2	128	268	202	no
2	[TCCTCGGTAGTATAGTGGTGAG------------]	139	75	122	194	no
2	[--CTCGGTAGTATAGTGGTGAGT-----------]	3	62	104	107	no
2	[TCCTCGGTAGTATAGTGGTGA-------------]	247	299	418	697	no
2	[TCCTCGGTAGTATAGTGGTG--------------]	314	385	641	1017	no
2	[TCCTCGGTAGTATAGTGGT---------------]	91	109	142	227	no
2	[--CTCGGTAGTATAGTGGTG--------------]	5	64	83	101	no
2	[TCCTCGGTAGTATAGTGG----------------]	39	70	97	227	no
2	[TCCTCGGTAGTATAGT------------------]	40	59	63	133	no
3	[TCCTTAGGTCACTGGTTCGAATCCGGTTCGACGG]	0	2	418	5	yes
4	[ATGGGAGAGGGCTGGGGTTCAATTCCCCATA]	1	0	6	32	yes
4	[-TGGGAGAGGGCTGGGGTTCAATTCCCCATA]	1	2	11	129	no
4	[ATGGGAGAGGGCTGGGGTTCAATTCCCCA--]	4	5	35	242	no
4	[-TGGGAGAGGGCTGGGGTTCAATTCCCCAT-]	0	9	52	249	no
4	[ATGGGAGAGGGCTGGGGTTCAATTCCCC---]	41	8	23	128	no
4	[-TGGGAGAGGGCTGGGGTTCAATTCCCCA--]	11	11	62	342	no

4	[ATGGGAGAGGGCTGGGGTTCAATTCCC----]	85	38	97	178	no
4	[-TGGGAGAGGGCTGGGGTTCAATTCCC----]	39	16	60	59	no
4	[-----AGAGGGCTGGGGTTCAATTCCCC---]	112	21	19	37	no
4	[-----AGAGGGCTGGGGTTCAATTCCC----]	99	15	12	24	no
4	[ATGGGAGAGGGCTGGGGTTCAA---------]	2	62	120	142	no
4	[-----AGAGGGCTGGGGTTCAATTCC-----]	156	26	19	15	no
4, 26	[ATGGGAGAGGGCTGGGGTTCA----------]	10	67	134	184	no
4	[--GGGAGAGGGCTGGGGTTCAAT--------]	9	61	58	50	no
4	[-TGGGAGAGGGCTGGGGTTCAA---------]	1	39	102	105	no
4, 26	[ATGGGAGAGGGCTGGGGTTC-----------]	13	96	145	202	no
4	[--GGGAGAGGGCTGGGGTTCAA---------]	10	134	148	157	no
4, 26	[-TGGGAGAGGGCTGGGGTTCA----------]	7	56	91	147	no
4, 26	[ATGGGAGAGGGCTGGGGTT------------]	13	81	85	126	no
4	[-----------CTGGGGTTCAATTCCCCAT-]	0	11	54	44	no
4, 26	[--GGGAGAGGGCTGGGGTTCA----------]	10	185	181	243	no
4, 26	[-TGGGAGAGGGCTGGGGTTC-----------]	2	65	92	149	no
4, 26	[ATGGGAGAGGGCTGGGGT-------------]	14	97	192	284	no
4, 26	[--GGGAGAGGGCTGGGGTTC-----------]	21	695	582	727	no
4, 12, 26	[ATGGGAGAGGGCTGGGG--------------]	44	69	151	139	no
4, 26	[---GGAGAGGGCTGGGGTTC-----------]	2	63	87	163	no
4, 26	[--GGGAGAGGGCTGGGGTT------------]	27	288	281	280	no
4, 26	[-TGGGAGAGGGCTGGGGT-------------]	9	39	122	185	no
4, 12, 26	[ATGGGAGAGGGCTGGG---------------]	325	175	307	267	no
4, 26	[---GGAGAGGGCTGGGGTT------------]	7	66	120	168	no
4, 26	[--GGGAGAGGGCTGGGGT-------------]	20	321	427	375	no
4, 26	[-----AGAGGGCTGGGGTTC-----------]	20	71	153	228	no
4, 12, 26	[ATGGGAGAGGGCTGG----------------]	351	317	824	1616	no
4, 26	[---GGAGAGGGCTGGGGT-------------]	19	116	231	380	no
4, 12, 26	[--GGGAGAGGGCTGGGG--------------]	8	164	219	170	no
5	[GCCGCCATAGCTCAGTCGGTTAGAGCGTGGG]	5	76	70	56	yes
5	[GCCGCCATAGCTCAGTCGGT-----------]	65	480	729	2107	no
5	[GCCGCCATAGCTCAGTCGG------------]	184	824	1518	4773	no
5	[GCCGCCATAGCTCAG----------------]	58	171	400	1260	no
6	[AAGCATTCGACGGGGGTTCGATTCCCCCC]	0	17	89	117	yes
6	[-AGCATTCGACGGGGGTTCGATTCCCCCC]	1	32	145	221	no
7	[CGCGGGTTCGAGCCCCGCATTGGGCTCCA]	60	10	6	3	yes
7	[--------------CCGCATTGGGCTCCA]	184	130	35	13	no
8	[GGGGGTATAGCTCAGTGGTAGAGCGCTCC]	4	288	358	113	yes
8	[GGGGGTATAGCTCAGTGGTAGAGCGCTC-]	4	110	120	62	no
8	[GGGGGTATAGCTCAGTGGTAGAGCGCT--]	20	206	122	35	no
8	[GGGGGTATAGCTCAGTGGTAGAGCGC---]	7	131	57	18	no
8	[GGGGGTATAGCTCAGTGGTAGAGCG----]	8	157	62	19	no
8	[GGGGGTATAGCTCAGTGG-----------]	9	65	147	364	no
8, 13	[GGGGGTATAGCTCAG--------------]	5	38	60	111	no
9	[ACTGGTTCGAATCCGGTTCGACGGACCA]	68	36	13	18	yes
9	[-CTGGTTCGAATCCGGTTCGACGGACCA]	219	313	139	209	no
9	[---GGTTCGAATCCGGTTCGACGGACCA]	120	116	25	16	no
9	[------TCGAATCCGGTTCGACGGACCA]	313	329	85	75	no

9	[-------CGAATCCGGTTCGACGGACCA]	133	252	61	34	no
9	[--------GAATCCGGTTCGACGGACCA]	132	221	60	37	no
9	[----------ATCCGGTTCGACGGACCA]	108	126	62	29	no
9	[-----------TCCGGTTCGACGGACCA]	360	186	122	73	no
9	[------------CCGGTTCGACGGACCA]	663	349	217	145	no
9	[-------------CGGTTCGACGGACCA]	1435	673	450	200	no
10	[GGGGGCATAGCTCAGAGGTAGAGCGCCC]	14	584	668	143	yes
10	[GGGGGCATAGCTCAGAGGTAGAGCGCC-]	14	471	662	81	no
11	[GTCGCGGGTTCGAGCCCCGCATTGGGCT]	3	11	123	139	yes
11	[GTCGCGGGTTCGAGCCCCGCATTG----]	65	186	284	234	no
11	[GTCGCGGGTTCGAGCCCCGCATT-----]	90	335	518	713	no
11	[GTCGCGGGTTCGAGCCCCGCAT------]	19	234	1529	1618	no
11	[GTCGCGGGTTCGAGCCCCGCA-------]	2	38	275	517	no
12	[CTCCCTTAGCATGGGAGAGGGCTGGGG]	14	17	124	189	yes
12	[CTCCCTTAGCATGGGAGAGGGCTGGG-]	12	28	115	140	no
12	[CTCCCTTAGCATGGGAGAGGGCTGG--]	74	128	393	467	no
12	[CTCCCTTAGCATGGGAGAGGGCTG---]	94	142	302	342	no
12	[-TCCCTTAGCATGGGAGAGGGCTGG--]	9	15	56	104	no
12	[-----TTAGCATGGGAGAGGGCTGGG-]	169	22	21	27	no
12	[-----TTAGCATGGGAGAGGGCTGG--]	254	37	28	38	no
12, 26	[---------CATGGGAGAGGGCTGGG-]	71	54	83	61	no
12, 26	[--------GCATGGGAGAGGGCTGG--]	12	53	121	193	no
12, 26	[---------CATGGGAGAGGGCTGG--]	66	53	94	115	no
13	[GGGGGTATAGCTCAGGGGTAGAGCGCT]	29	324	207	102	yes
13	[GGGGGTATAGCTCAGGGGTAGAGCGC-]	21	357	135	30	no
13	[GGGGGTATAGCTCAGGGGTAGAGCG--]	11	274	142	21	no
13	[GGGGGTATAGCTCAGGGGTAGAGC---]	11	185	67	43	no
14	[GAGTTCAAATCTCGGTGGGACCTCCA]	98	55	10	0	yes
15	[GGGGGCATAGCTCAGGGGTAGAGCGC]	19	712	271	56	yes
16	[TCCCTTCGGGGGCGTGGGTTCGAATC]	1	49	237	22	yes
16	[TCCCTTCGGGGGCGTGGGTTCGAAT-]	2	48	237	94	no
16	[TCCCTTCGGGGGCGTGGGTTCGAA--]	4	63	359	212	no
16	[TCCCTTCGGGGGCGTGGGTTCGA---]	5	27	185	191	no
16	[---CTTCGGGGGCGTGGGTTCGA---]	154	37	13	30	no
17	[TGGTGTAGCGGTTAGCACTCAGGACT]	0	2	2	128	yes
17	[---TGTAGCGGTTAGCACTCAGGACT]	7	12	93	218	no
17	[---TGTAGCGGTTAGCACTCA-----]	6	9	37	108	no
18	[GCAGTCATGTCCGAGTGGTTAAGGA]	4	3	20	122	yes
18	[GCAGTCATGTCCGAGTGGTTAAG--]	2	17	81	327	no
18	[GCAGTCATGTCCGAGTGGTTAA---]	7	44	284	1091	no
18	[GCAGTCATGTCCGAGTGGTTA----]	5	23	144	639	no
18	[GCAGTCATGTCCGAGTGGTT-----]	5	13	71	322	no
18	[GCAGTCATGTCCGAGT---------]	14	27	54	250	no
18	[GCAGTCATGTCCGAG----------]	8	21	151	894	no
19	[GGTTCCATGGTGTAGCGGTTAGCAC]	30	17	32	190	yes
19	[GGTTCCATGGTGTAGCGGTTAGCA-]	129	57	169	671	no
19	[GGTTCCATGGTGTAGCGGTTAGC--]	23	20	76	492	no
19	[GGTTCCATGGTGTAGCGGTTAG---]	21	53	285	919	no

19	[GGTTCCATGGTGTAGCGGTTA----]	4	33	84	337	no
19	[GGTTCCATGGTGTAGCGGTT-----]	31	116	330	1090	no
19	[GGTTCCATGGTGTAGCGGT------]	9	65	136	295	no
19	[GGTTCCATGGTGTAGC---------]	51	104	173	438	no
19	[GGTTCCATGGTGTAG----------]	37	192	281	833	no
20	[TGGGCTCTGCCCGCGTAGGTTCGAA]	1	24	214	57	yes
20	[TGGGCTCTGCCCGCGTAGGTTCGA-]	4	17	133	67	no
21	[CTACGGAGCAGAAGATTGTAGGTT]	307	224	77	12	yes
21	[CTACGGAGCAGAAGATTGTAGGT-]	522	583	221	25	no
21	[CTACGGAGCAGAAGATTGTAGG--]	171	246	100	24	no
21	[-----GAGCAGAAGATTGTAGGTT]	27	156	62	30	no
21	[-----GAGCAGAAGATTGTAGGT-]	57	408	82	26	no
21, 38	[-------GCAGAAGATTGTAGGTT]	257	528	122	140	no
21, 38	[-------GCAGAAGATTGTAGGT-]	101	438	130	84	no
22	[GTTCGAATCCTGCTGACTGCGCCA]	611	77	20	15	yes
22	[-TTCGAATCCTGCTGACTGCGCCA]	833	248	92	44	no
22	[--TCGAATCCTGCTGACTGCGCCA]	1260	467	133	69	no
22	[---CGAATCCTGCTGACTGCGCCA]	624	542	64	48	no
22	[----GAATCCTGCTGACTGCGCCA]	62	74	25	19	no
22	[-----AATCCTGCTGACTGCGCCA]	66	100	35	29	no
22	[------ATCCTGCTGACTGCGCCA]	91	131	32	30	no
22	[--------CCTGCTGACTGCGCCA]	50	183	175	169	no
23	[AGAAGGTCGGCGGTTCGATCCCG]	5	516	1119	583	yes
23	[AGAAGGTCGGCGGTT--------]	1	446	357	250	no
24	[CTAGTGGTTAGGATTCGTGGTTT]	0	21	54	225	yes
24	[CTAGTGGTTAGGATTCGTGGTT-]	3	144	645	2495	no
24	[CTAGTGGTTAGGATTCGTGGT--]	4	57	181	320	no
24	[-TAGTGGTTAGGATTCGTGGTT-]	3	20	118	295	no
24	[--AGTGGTTAGGATTCGTGGTT-]	4	69	193	440	no
25	[CTAGTGGTTAGGATTTATGGCTC]	137	21	45	117	yes
25	[--AGTGGTTAGGATTTATGGCT-]	30	208	356	434	no
26	[GCATGGGAGAGGGCTGGGGTTCA]	11	77	70	72	yes
27	[GGTCTCGTGGTGTAGTGGTTATC]	41	43	144	293	yes
27	[GGTCTCGTGGTGTAGTGGTTAT-]	25	51	194	429	no
28	[GTCGCTGGTTCGATTCCAGCATG]	6	122	435	587	yes
29	[TTCAATTCCCCATACCTCCACCA]	103	53	9	12	yes
29	[-TCAATTCCCCATACCTCCACCA]	488	282	92	100	no
29	[--CAATTCCCCATACCTCCACCA]	561	354	69	76	no
29	[---AATTCCCCATACCTCCACCA]	258	213	64	96	no
29	[----ATTCCCCATACCTCCACCA]	2767	1933	360	485	no
29	[----ATTCCCCATACCTCCACC-]	3	16	25	118	no
29	[-----TTCCCCATACCTCCACCA]	1625	1125	436	505	no
29	[------TCCCCATACCTCCACCA]	422	352	182	196	no
29	[-------CCCCATACCTCCACCA]	105	212	136	157	no
29	[--------CCCATACCTCCACCA]	92	259	141	157	no
30	[GATCAGGTGGCCGAGTGGTTAA]	3	20	65	280	yes
30	[GATCAGGTGGCCGAGTGGTTA-]	11	52	154	847	no
30	[GATCAGGTGGCCGAGTGGTT--]	13	120	196	1075	no

30	[GATCAGGTGGCCGAGTGG----]	15	45	138	756	no
30	[GATCAGGTGGCCGAGTG-----]	7	61	177	979	no
30	[GATCAGGTGGCCGAGT------]	7	51	79	321	no
30	[GATCAGGTGGCCGAG-------]	13	47	197	695	no
31	[GGCCGGATGGTCTAGAGGTATG]	204	659	2056	2503	yes
31	[GGCCGGATGGTCTAGAGGTAT-]	160	830	2374	2953	no
31	[GGCCGGATGGTCTAGAGGTA--]	33	326	1061	1081	no
32	[TCGAATCCTGCCGTGGTCGCCA]	368	353	74	43	yes
32	[-------CTGCCGTGGTCGCCA]	26	137	75	53	no
33	[ATAAGGCCGGGGTTCGATTCC]	81	22	9	16	yes
34	[CAGGGGTTCGAGTCCCCTAGG]	18	78	284	207	yes
35	[CGATTCCCCGCAACGGAACCA]	84	130	16	13	yes
35	[--ATTCCCCGCAACGGAACCA]	146	218	42	45	no
36	[GCACGGATGGCCGAGTGGTCT]	8	48	95	545	yes
36	[GCACGGATGGCCGAGTGG---]	16	91	322	967	no
36	[GCACGGATGGCCGAGTG----]	7	62	260	821	no
36	[GCACGGATGGCCGAGT-----]	11	150	453	1366	no
36	[-CACGGATGGCCGAGT-----]	15	159	333	537	no
36	[GCACGGATGGCCGAG------]	19	122	903	2667	no
37	[GGCCGGATGGTCTAGTGGTAT]	47	119	370	471	yes
38	[AGCAGAAGATTGTAGGTTCG]	20	97	166	333	yes
39	[CAGTGGAAGCGTGCTGGGCC]	256	31	30	45	yes
40	[GGAGAGATGGCCGAGCGGTC]	12	78	119	515	yes
40	[GGAGAGATGGCCGAGCGGT-]	23	84	101	383	no
40	[GGAGAGATGGCCGAGCGG--]	24	107	446	1146	no
40	[GGAGAGATGGCCGAGCG---]	78	140	501	1058	no
40	[GGAGAGATGGCCGAGC----]	35	209	441	1031	no
40	[GGAGAGATGGCCGAG-----]	35	208	692	1888	no
41	[ACCCCTGCTGGCGGCACCA]	754	362	95	150	yes
41	[-CCCCTGCTGGCGGCACCA]	506	381	97	66	no
42	[AGACCCGGGTTCAATTCCC]	157	97	72	136	yes
43	[ATTCCCCCCGAACGCACCA]	139	112	13	11	yes
44	[GCCCCATTGGCGCAGTCGG]	1	12	157	900	yes
45	[GCCTCCTTAGCTCAGTTGG]	7	119	172	1365	yes
46	[GGGTTCGATTCCCGGTCGA]	3	29	152	434	yes
47	[ACCCGGCCAGGACCTCCA]	88	37	6	2	yes
47	[-CCCGGCCAGGACCTCCA]	200	125	29	11	no
48	[ATCCGGCTCGACGGACCA]	163	75	34	9	yes
48	[--CCGGCTCGACGGACCA]	228	61	43	27	no
48	[---CGGCTCGACGGACCA]	335	133	90	64	no
49	[GGCCGCGTGGCGCAATGG]	5	411	108	41	yes
49	[GGCCGCGTGGCGCAATG-]	2	207	70	18	no
50	[GGTCCTGTGGTGTAGTGG]	31	50	134	327	yes
50	[GGTCCTGTGGTGTAG---]	3	22	112	310	no
51	[AGCACGATGGCCGAGTG]	5	18	102	331	yes
52	[CCTGGTTCGGGGCACCA]	145	408	81	73	yes
53	[TCGAAAGGTTGGGCGTT]	548	871	799	752	yes
53	[TCGAAAGGTTGGGCGT-]	633	542	292	189	no

54	[GCAGAAGGTCGCCGGT]	285	427	171	140	yes
55	[GGTGAGATGGCCGAGT]	7	95	302	1494	yes
55	[GGTGAGATGGCCGAG-]	23	123	510	2155	no
56	[CCGGCATGGGAACCA]	487	602	330	94	yes
57	[CTGCGGACGGCACCA]	278	579	547	367	yes

Supplementary Table S6. True positive tRFs after filtering

tRNA homology group	tRNA origin (gene copy)	Total tRNA gene copy	tRF_seq	tRF type	tRF length	Count mapped read			
						Day 0	Day 5	Day 8	Day 12
1	Ala_AGC (22), Ala_CGC (4), Ala_TGC (8), Thr_AGT (1)	35	GGGGGTATAGCTCAGTGGTAGAGCGCTCC	5'-end	29	279	1253	1197	1012
1	Ala_AGC (22), Ala_CGC (4), Ala_TGC (8), Thr_AGT (1)	35	GGGGGCATAGCTCAGAGGTAGAGCGCCC	5'-end	28	370	2120	2633	1624
1	Ala_AGC (22), Ala_CGC (4), Ala_TGC (8), Thr_AGT (1)	35	GGGGGCATAGCTCAGGGGTAGAGCGC	5'-end	26	166	1807	1631	1440
1	Ala_AGC (22), Ala_CGC (4), Ala_TGC (8), Thr_AGT (1)	35	GGGGGTATAGCTCAGGGGTAGAGCGCT	5'-end	27	192	1426	1011	788
2	Arg_ACG (19)	19	GGCCGCGTGGCGCAATGG	5'-end	18	9	672	233	68
9	Asp_GTC (27)	27	TCCTCGGTAGTATAGTGGTGAGTATCCGCGTCTG	5'-end	34	1500	2571	4787	7588
11	Gln_CTG (7), Gln_TTG (19)	26	GGTTCCATGGTGTAGCGGTTAGCAC	5'-end	25	402	782	1911	6389
11	Gln_CTG (7), Gln_TTG (19)	26	TGGTGTAGCGGTTAGCACTCAGGACT	5'-end	26	44	121	443	1227
12	Glu_CTC (24), Glu_TTC (17)	41	CTAGTGGTTAGGATTTATGGCTC	5'-end	23	195	369	676	1026
12	Glu_CTC (24), Glu_TTC (17)	41	CTAGTGGTTAGGATTCGTGGTTT	5'-end	23	133	470	1742	5098
16	Ile_AAT (21), Ile_TAT (3), Lys_TTT (15), Met_CAT (9), Thr_CGT (1), Thr_TGT (9)	58	GCCGCCATAGCTCAGTCGGTTAGAGCGTGGG	5'-end	31	509	2309	3803	10910
16	Ile_AAT (21), Ile_TAT (3), Lys_TTT (15), Met_CAT (9), Thr_CGT (1), Thr_TGT (9)	58	GCCCCATTGGCGCAGTCGG	5'-end	19	2	17	175	931
16	Ile_AAT (21), Ile_TAT (3), Lys_TTT (15), Met_CAT (9), Thr_CGT (1), Thr_TGT (9)	58	GCCTCCTTAGCTCAGTTGG	5'-end	19	12	218	352	2405
17	Leu_AAG (19), Leu_TAA (1), Leu_TAG (3)	23	GGAGAGATGGCCGAGCGGTC	5'-end	20	215	862	2430	6262
17	Leu_AAG (19), Leu_TAA (1), Leu_TAG (3)	23	GGTGAGATGGCCGAGT	5'-end	16	30	220	815	3668
18	Leu_CAA (7), Leu_TAA (3), Ser_GCT (9)	19	GCACGGATGGCCGAGTGGTCT	5'-end	21	145	979	2717	8069
18	Leu_CAA (7), Leu_TAA (3), Ser_GCT (9)	19	AGCACGATGGCCGAGTG	5'-end	17	13	52	234	674
18	Leu_CAA (7), Leu_TAA (3), Ser_GCT (9)	19	GATCAGGTGGCCGAGTGGTTAA	5'-end	22	79	455	1083	5207
21	Met_CAT (10)	10	CAGTGGAAGCGTGCTGGGCC	5'-end	20	567	158	399	1071
23	Pro_AGG (6), Pro_CGG (4), Pro_TGG (32)	42	GGCCGGATGGTCTAGTGGTAT	5'-end	21	87	272	746	911
23	Pro_AGG (6), Pro_CGG (4), Pro_TGG (32)	42	GGCCGGATGGTCTAGAGGTATG	5'-end	22	458	2180	6333	7554
23	Pro_AGG (6), Pro_CGG (4), Pro_TGG (32)	42	GGCCGAATGGTCTAGTGGTATGATTCTCGCTTTG	5'-end	34	161	645	1037	1117
24	Ser_AGA (15), Ser_CGA (6)	21	GCAGTCATGTCCGAGTGGTTAAGGA	5'-end	25	47	166	881	3933
29	Val_AAC (19)	19	GGTCTCGTGGTGTAGTGGTTATC	5'-end	23	126	363	930	1704
30	Met_CAT (1), Val_CAC (6), Val_TAC (5)	12	GGTCCTGTGGTGTAGTGG	5'-end	18	39	87	288	828
1	Ala_AGC (22), Ala_CGC (4), Ala_TGC (8), Thr_AGT (1)	35	ATGGGAGAGGGCTGGGGTTCAATTCCCCATA	3'-end (no CCA)	31	2203	4152	6642	10174
9	Asp_GTC (27)	27	AGACCCGGGTTCAATTCCC	3'-end (no CCA)	19	186	128	129	273
12	Glu_CTC (24), Glu_TTC (17)	41	ATAAGGCCGGGGTTCGATTCC	3'-end (no CCA)	21	529	175	110	187
13	Gly_GCC (15)	15	GGGTTCGATTCCCGGTCGA	3'-end (no CCA)	19	7	39	178	512
14	Gly_TCC (36)	36	AAGCATTCGACGGGGGTTCGATTCCCCCC	3'-end (no CCA)	29	99	262	731	1280
16	Ile_AAT (21), Ile_TAT (3), Lys_TTT (15), Met_CAT (9), Thr_CGT (1), Thr_TGT (9)	58	CAGGGGTTCGAGTCCCCTAGG	3'-end (no CCA)	21	19	133	477	475
20	Lys_CTT (30)	30	GTCGCGGGTTCGAGCCCCGCATTGGGCT	3'-end (no CCA)	28	205	961	3632	4370

26	Thr_AGT (16), Thr_CGT (6)	22	GTCGCTGGTTCGATTCCAGCATG	3'-end (no CCA)	23	10	196	904	1499
28	Tyr_GTA (19)	19	TCCTTAGGTCACTGGTTCGAATCCGGTTCGACGG	3'-end (no CCA)	34	101	109	658	648
29	Val_AAC (19)	19	AGAAGGTCGGCGGTTCGATCCCG	3'-end (no CCA)	23	29	2045	2321	1641
1	Ala_AGC (22), Ala_CGC (4), Ala_TGC (8), Thr_AGT (1)	35	TTCAATTCCCCATACCTCCACCA	3'-end (CCA)	23	6467	4924	1808	2618
2	Arg_ACG (19)	19	TCGAATCCTGCCGTGGTCGCCA	3'-end (CCA)	22	550	813	310	240
11	Gln_CTG (7), Gln_TTG (19)	26	GAGTTCAAATCTCGGTGGGACCTCCA	3'-end (CCA)	26	376	379	391	310
12	Glu_CTC (24), Glu_TTC (17)	41	CGATTCCCCGCAACGGAACCA	3'-end (CCA)	21	467	621	183	194
12	Glu_CTC (24), Glu_TTC (17)	41	CCGGCATGGGAACCA	3'-end (CCA)	15	487	602	330	94
14	Gly_TCC (36)	36	ATTCCCCCCGAACGCACCA	3'-end (CCA)	19	201	168	39	49
16	Ile_AAT (21), Ile_TAT (3), Lys_TTT (15), Met_CAT (9), Thr_CGT (1), Thr_TGT (9)	58	ACCCCTGCTGGCGGCACCA	3'-end (CCA)	19	1607	1055	393	430
19	Leu_CAG (6)	6	CTGCGGACGGCACCA	3'-end (CCA)	15	278	579	547	367
20	Lys_CTT (30)	30	CGCGGGTTCGAGCCCCGCATTGGGCTCCA	3'-end (CCA)	29	579	476	458	542
22	Phe_GAA (14)	14	CCTGGTTCGGGGCACCA	3'-end (CCA)	17	266	657	178	183
24	Ser_AGA (15), Ser_CGA (6)	21	GTTCGAATCCTGCTGACTGCGCCA	3'-end (CCA)	24	3857	2027	860	995
28	Tyr_GTA (19)	19	ACTGGTTCGAATCCGGTTCGACGGACCA	3'-end (CCA)	28	3903	3286	1999	2416
28	Tyr_GTA (19)	19	ATCCGGCTCGACGGACCA	3'-end (CCA)	18	925	314	243	170
30	Met_CAT (1), Val_CAC (6), Val_TAC (5)	12	ACCCGGCCAGGACCTCCA	3'-end (CCA)	18	345	204	68	31
1	Ala_AGC (22), Ala_CGC (4), Ala_TGC (8), Thr_AGT (1)	35	CTCCCTT**AGC**ATGGGAGAGGGCTGGGG	middle	27	1982	1628	3592	4780
1	Ala_AGC (22), Ala_CGC (4), Ala_TGC (8), Thr_AGT (1)	35	**GC**ATGGGAGAGGGCTGGGGTTCA	middle	23	1339	3821	5779	7686
2	Arg_ACG (19)	19	AGCAGAAGATTGTAGGTTCG	middle	20	537	1721	964	1268
2	Arg_ACG (19)	19	CT**ACG**GAGCAGAAGATTGTAGGTT	middle	24	1668	3482	1379	764
17	Leu_AAG (19), Leu_TAA (1), Leu_TAG (3)	23	TCCCTTCGGGGGCGTGGGTTCGAATC	middle	26	743	543	1902	1434
24	Ser_AGA (15), Ser_CGA (6)	21	TGGGCTCTGCCCGCGTAGGTTCGAA	middle	25	171	183	597	450
27	Trp_CCA (12)	12	TCGAAAGGTTGGGCGTT	middle	17	1268	1590	1326	1211
30	Met_CAT (1), Val_CAC (6), Val_TAC (5)	12	GCAGAAGGTCGCCGGT	middle	16	323	493	215	199

Supplementary Table S7. Base positions for coverage analysis

Target	tRNA	Strand dir	Chr	Start	End
A at 1st base position of anticodon	Arg_ACG	-1	I	5843124	5843125
A at 1st base position of anticodon	Pro_AGG	-1	I	5854835	5854836
A at 1st base position of anticodon	Leu_AAG	-1	I	6051203	6051204
A at 1st base position of anticodon	Ile_AAT	-1	I	6163511	6163512
A at 1st base position of anticodon	Ile_AAT	1	I	6164226	6164227
A at 1st base position of anticodon	Val_AAC	-1	I	6212860	6212861
A at 1st base position of anticodon	Pro_AGG	-1	I	9268959	9268960
A at 1st base position of anticodon	Leu_AAG	-1	I	11585407	11585408
A at 1st base position of anticodon	Val_AAC	1	I	12065888	12065889
A at 1st base position of anticodon	Ala_AGC	1	I	12601795	12601796
A at 1st base position of anticodon	Leu_AAG	-1	I	12731686	12731687
A at 1st base position of anticodon	Val_AAC	-1	I	13156384	13156385
A at 1st base position of anticodon	Val_AAC	1	I	13161082	13161083
A at 1st base position of anticodon	Thr_AGT	1	II	1334171	1334172
A at 1st base position of anticodon	His_ATG	1	II	1481957	1481958
A at 1st base position of anticodon	Ser_AGA	1	II	1520074	1520075
A at 1st base position of anticodon	Leu_AAG	-1	II	2735845	2735846
A at 1st base position of anticodon	Leu_AAG	-1	II	2736327	2736328
A at 1st base position of anticodon	Leu_AAG	1	II	2860380	2860381
A at 1st base position of anticodon	Leu_AAG	-1	II	2861601	2861602
A at 1st base position of anticodon	Ser_AGA	1	II	3268025	3268026
A at 1st base position of anticodon	Thr_AGT	-1	II	3439260	3439261
A at 1st base position of anticodon	Thr_AGT	-1	II	3519127	3519128
A at 1st base position of anticodon	Thr_AGT	-1	II	3567651	3567652
A at 1st base position of anticodon	Ala_AGC	-1	II	4565051	4565052
A at 1st base position of anticodon	Ala_AGC	1	II	4565417	4565418
A at 1st base position of anticodon	Ile_AAT	1	II	5032795	5032796
A at 1st base position of anticodon	Ile_AAT	-1	II	5057477	5057478
A at 1st base position of anticodon	Ile_AAT	1	II	5237882	5237883
A at 1st base position of anticodon	Ala_AGC	1	II	5292346	5292347
A at 1st base position of anticodon	Ala_AGC	-1	II	7002989	7002990
A at 1st base position of anticodon	Thr_AGT	1	II	7003262	7003263
A at 1st base position of anticodon	His_ATG	-1	II	12349609	12349610
A at 1st base position of anticodon	Ile_AAT	1	II	12362418	12362419
A at 1st base position of anticodon	Ile_AAT	1	II	12494710	12494711
A at 1st base position of anticodon	Leu_AAG	-1	II	12541912	12541913
A at 1st base position of anticodon	Ala_AGC	1	II	12542601	12542602
A at 1st base position of anticodon	Leu_AAG	-1	II	14635306	14635307
A at 1st base position of anticodon	Ile_AAT	-1	III	1163383	1163384
A at 1st base position of anticodon	Ile_AAT	-1	III	1164829	1164830
A at 1st base position of anticodon	Ile_AAT	-1	III	1218406	1218407
A at 1st base position of anticodon	His_ATG	1	III	2879939	2879940
A at 1st base position of anticodon	Leu_AAG	1	III	3099598	3099599
A at 1st base position of anticodon	Arg_ACG	-1	III	3339502	3339503
A at 1st base position of anticodon	Ala_AGC	-1	III	4428963	4428964

A at 1st base position of anticodon	Arg_ACG	-1	III	4449540	4449541
A at 1st base position of anticodon	Arg_ACG	-1	III	4450331	4450332
A at 1st base position of anticodon	Leu_AAG	1	III	5769892	5769893
A at 1st base position of anticodon	Ser_AGA	1	III	6158758	6158759
A at 1st base position of anticodon	Thr_AGT	-1	III	7030538	7030539
A at 1st base position of anticodon	Ser_AGA	1	III	8646119	8646120
A at 1st base position of anticodon	Ser_AGA	-1	III	9861982	9861983
A at 1st base position of anticodon	Thr_AGT	-1	III	10624681	10624682
A at 1st base position of anticodon	Val_AAC	1	III	11352961	11352962
A at 1st base position of anticodon	Val_AAC	1	III	11353560	11353561
A at 1st base position of anticodon	His_ATG	-1	IV	1146111	1146112
A at 1st base position of anticodon	Tyr_ATA	1	IV	1407295	1407296
A at 1st base position of anticodon	Arg_ACG	1	IV	3524847	3524848
A at 1st base position of anticodon	Ala_AGC	1	IV	6562222	6562223
A at 1st base position of anticodon	Pro_AGG	1	IV	11231574	11231575
A at 1st base position of anticodon	Arg_ACG	-1	IV	12417941	12417942
A at 1st base position of anticodon	Arg_ACG	-1	IV	14611317	14611318
A at 1st base position of anticodon	Val_AAC	1	IV	15235388	15235389
A at 1st base position of anticodon	Ser_AGA	-1	IV	15311133	15311134
A at 1st base position of anticodon	Ser_AGA	-1	IV	15318493	15318494
A at 1st base position of anticodon	Val_AAC	-1	IV	16382437	16382438
A at 1st base position of anticodon	Ala_AGC	-1	IV	16389217	16389218
A at 1st base position of anticodon	Ala_AGC	1	IV	16398383	16398384
A at 1st base position of anticodon	Ala_AGC	1	IV	16399183	16399184
A at 1st base position of anticodon	Val_AAC	1	IV	16400130	16400131
A at 1st base position of anticodon	Ala_AGC	1	V	857357	857358
A at 1st base position of anticodon	Thr_AGT	-1	V	2664703	2664704
A at 1st base position of anticodon	Thr_AGT	1	V	2674039	2674040
A at 1st base position of anticodon	Thr_AGT	-1	V	8496121	8496122
A at 1st base position of anticodon	Thr_AGT	-1	V	9407054	9407055
A at 1st base position of anticodon	Ala_AGC	-1	V	13003532	13003533
A at 1st base position of anticodon	Val_AAC	-1	V	15013055	15013056
A at 1st base position of anticodon	His_ATG	-1	V	17660789	17660790
A at 1st base position of anticodon	Arg_ACG	1	V	17662177	17662178
A at 1st base position of anticodon	His_ATG	1	V	17686387	17686388
A at 1st base position of anticodon	His_ATG	1	V	17690514	17690515
A at 1st base position of anticodon	His_ATG	-1	V	17765696	17765697
A at 1st base position of anticodon	Thr_AGT	-1	V	17786896	17786897
A at 1st base position of anticodon	Gly_ACC	1	V	17930357	17930358
A at 1st base position of anticodon	Gly_ACC	1	V	17932767	17932768
A at 1st base position of anticodon	His_ATG	1	V	18367143	18367144
A at 1st base position of anticodon	His_ATG	1	V	18371233	18371234
A at 1st base position of anticodon	Gly_ACC	-1	V	18801983	18801984
A at 1st base position of anticodon	Gly_ACC	1	V	18825989	18825990
A at 1st base position of anticodon	Gly_ACC	-1	V	18827884	18827885
A at 1st base position of anticodon	Gly_ACC	-1	V	18829056	18829057
A at 1st base position of anticodon	Thr_AGT	-1	V	19059010	19059011
A at 1st base position of anticodon	His_ATG	1	V	19393523	19393524

A at 1st base position of anticodon	His_ATG	-1	V	19394789	19394790
A at 1st base position of anticodon	His_ATG	-1	V	19396704	19396705
A at 1st base position of anticodon	His_ATG	-1	V	19396998	19396999
A at 1st base position of anticodon	His_ATG	1	V	19398589	19398590
A at 1st base position of anticodon	His_ATG	1	V	19577392	19577393
A at 1st base position of anticodon	His_ATG	-1	V	19578473	19578474
A at 1st base position of anticodon	His_ATG	-1	V	19578778	19578779
A at 1st base position of anticodon	His_ATG	-1	V	19594048	19594049
A at 1st base position of anticodon	His_ATG	-1	V	19594811	19594812
A at 1st base position of anticodon	His_ATG	-1	V	19595595	19595596
A at 1st base position of anticodon	His_ATG	-1	V	19624052	19624053
A at 1st base position of anticodon	Asp_ATC	1	V	19629830	19629831
A at 1st base position of anticodon	His_ATG	-1	V	20016582	20016583
A at 1st base position of anticodon	His_ATG	-1	V	20021098	20021099
A at 1st base position of anticodon	His_ATG	1	V	20219300	20219301
A at 1st base position of anticodon	Leu_AAG	-1	X	353301	353302
A at 1st base position of anticodon	Asp_ATC	-1	X	1474049	1474050
A at 1st base position of anticodon	Ile_AAT	-1	X	1589700	1589701
A at 1st base position of anticodon	Ile_AAT	-1	X	1590113	1590114
A at 1st base position of anticodon	Ile_AAT	-1	X	1590541	1590542
A at 1st base position of anticodon	Asp_ATC	1	X	1620301	1620302
A at 1st base position of anticodon	Asp_ATC	1	X	1643876	1643877
A at 1st base position of anticodon	Ile_AAT	-1	X	2526249	2526250
A at 1st base position of anticodon	Thr_AGT	1	X	2528066	2528067
A at 1st base position of anticodon	Ile_AAT	1	X	2531387	2531388
A at 1st base position of anticodon	Thr_AGT	-1	X	3371447	3371448
A at 1st base position of anticodon	Pro_AGG	1	X	3970107	3970108
A at 1st base position of anticodon	Pro_AGG	1	X	3970276	3970277
A at 1st base position of anticodon	Ile_AAT	1	X	4242535	4242536
A at 1st base position of anticodon	Thr_AGT	1	X	4445020	4445021
A at 1st base position of anticodon	Ile_AAT	-1	X	5085629	5085630
A at 1st base position of anticodon	Arg_ACG	-1	X	6220073	6220074
A at 1st base position of anticodon	Leu_AAG	-1	X	6903120	6903121
A at 1st base position of anticodon	Thr_AGT	-1	X	7176978	7176979
A at 1st base position of anticodon	Ala_AGC	1	X	7323738	7323739
A at 1st base position of anticodon	Ala_AGC	-1	X	7378776	7378777
A at 1st base position of anticodon	Ala_AGC	-1	X	7507283	7507284
A at 1st base position of anticodon	Arg_ACG	-1	X	8036674	8036675
A at 1st base position of anticodon	Pro_AGG	1	X	8037489	8037490
A at 1st base position of anticodon	Ser_AGA	-1	X	8152810	8152811
A at 1st base position of anticodon	Ser_AGA	1	X	8154428	8154429
A at 1st base position of anticodon	Ser_AGA	1	X	8155445	8155446
A at 1st base position of anticodon	Ile_AAT	-1	X	8156623	8156624
A at 1st base position of anticodon	Leu_AAG	-1	X	8409289	8409290
A at 1st base position of anticodon	Leu_AAG	-1	X	8417905	8417906
A at 1st base position of anticodon	Leu_AAG	-1	X	8419407	8419408
A at 1st base position of anticodon	Arg_ACG	1	X	8623583	8623584
A at 1st base position of anticodon	Arg_ACG	-1	X	8624446	8624447

A at 1st base position of anticodon	Arg_ACG	-1	X	8639211	8639212
A at 1st base position of anticodon	Arg_ACG	-1	X	8640780	8640781
A at 1st base position of anticodon	Val_AAC	-1	X	8650695	8650696
A at 1st base position of anticodon	Leu_AAG	1	X	8939529	8939530
A at 1st base position of anticodon	Leu_AAG	-1	X	8955912	8955913
A at 1st base position of anticodon	Ser_AGA	1	X	8971578	8971579
A at 1st base position of anticodon	Ala_AGC	1	X	9237783	9237784
A at 1st base position of anticodon	Ala_AGC	1	X	9238836	9238837
A at 1st base position of anticodon	Val_AAC	-1	X	9561069	9561070
A at 1st base position of anticodon	Ala_AGC	-1	X	9622805	9622806
A at 1st base position of anticodon	Ala_AGC	-1	X	9637833	9637834
A at 1st base position of anticodon	Ala_AGC	1	X	9743720	9743721
A at 1st base position of anticodon	Arg_ACG	-1	X	10018405	10018406
A at 1st base position of anticodon	Arg_ACG	1	X	10132651	10132652
A at 1st base position of anticodon	Arg_ACG	-1	X	10132950	10132951
A at 1st base position of anticodon	Arg_ACG	1	X	10145129	10145130
A at 1st base position of anticodon	Arg_ACG	-1	X	10148628	10148629
A at 1st base position of anticodon	Arg_ACG	-1	X	10731962	10731963
A at 1st base position of anticodon	Leu_AAG	-1	X	11162899	11162900
A at 1st base position of anticodon	Leu_AAG	-1	X	11163338	11163339
A at 1st base position of anticodon	Arg_ACG	-1	X	11252563	11252564
A at 1st base position of anticodon	Ala_AGC	-1	X	12458146	12458147
A at 1st base position of anticodon	Val_AAC	1	X	12843650	12843651
A at 1st base position of anticodon	Leu_AAG	1	X	12942421	12942422
A at 1st base position of anticodon	Ser_AGA	1	X	13046541	13046542
A at 1st base position of anticodon	Thr_AGT	1	X	13261165	13261166
A at 1st base position of anticodon	Thr_AGT	-1	X	13283606	13283607
A at 1st base position of anticodon	Thr_AGT	1	X	13293154	13293155
A at 1st base position of anticodon	Ile_AAT	-1	X	13415090	13415091
A at 1st base position of anticodon	Ile_AAT	1	X	13435931	13435932
A at 1st base position of anticodon	Ser_AGA	1	X	14162178	14162179
A at 1st base position of anticodon	Ile_AAT	1	X	14679124	14679125
A at 1st base position of anticodon	Val_AAC	1	X	15236087	15236088
A at 1st base position of anticodon	Ile_AAT	1	X	15297435	15297436
A at 1st base position of anticodon	Ser_AGA	-1	X	15769988	15769989
A at 1st base position of anticodon	Ser_AGA	1	X	15773491	15773492
A at 1st base position of anticodon	Val_AAC	1	X	16298301	16298302
A at 1st base position of anticodon	Val_AAC	1	X	16299353	16299354
A at 1st base position of anticodon	Val_AAC	1	X	16300023	16300024
A at 1st base position of anticodon	Val_AAC	1	X	16300549	16300550
A at 1st base position of anticodon	Val_AAC	1	X	17611245	17611246
A at 1st base position of anticodon	Val_AAC	-1	X	17611465	17611466
Three additional base positions from the 3'-end	Lys_CTT	1	I	185409	185412
Three additional base positions from the 3'-end	Leu_CAG	1	I	754766	754769
Three additional base positions from the 3'-end	Sup_CTA	1	I	803875	803878
Three additional base positions from the 3'-end	Trp_CCA	-1	I	946043	946046
Three additional base positions from the 3'-end	Arg_TCT	1	I	1447212	1447215
Three additional base positions from the 3'-end	Arg_TCT	1	I	1575794	1575797

Three additional base positions from the 3'-end	Gly_GCC	-1	I	2272366	2272369
Three additional base positions from the 3'-end	Met_CAT	-1	I	3638851	3638854
Three additional base positions from the 3'-end	Arg_ACG	-1	I	5843082	5843085
Three additional base positions from the 3'-end	Pro_AGG	-1	I	5854793	5854796
Three additional base positions from the 3'-end	Leu_AAG	-1	I	6051153	6051156
Three additional base positions from the 3'-end	Tyr_GTA	1	I	6135915	6135918
Three additional base positions from the 3'-end	Lys_CTT	1	I	6136671	6136674
Three additional base positions from the 3'-end	Ile_AAT	-1	I	6163469	6163472
Three additional base positions from the 3'-end	Ile_AAT	1	I	6164266	6164269
Three additional base positions from the 3'-end	Val_AAC	-1	I	6212819	6212822
Three additional base positions from the 3'-end	Cys_GCA	1	I	6781875	6781878
Three additional base positions from the 3'-end	Lys_CTT	-1	I	7279349	7279352
Three additional base positions from the 3'-end	Asn_GTT	-1	I	7718874	7718877
Three additional base positions from the 3'-end	Lys_CTT	1	I	7842983	7842986
Three additional base positions from the 3'-end	Ile_TAT	1	I	7960916	7960919
Three additional base positions from the 3'-end	Glu_CTC	-1	I	8530118	8530121
Three additional base positions from the 3'-end	Undet_???	-1	I	8781144	8781147
Three additional base positions from the 3'-end	Lys_CTT	1	I	8791420	8791423
Three additional base positions from the 3'-end	Lys_TTT	1	I	8953234	8953237
Three additional base positions from the 3'-end	Asn_GTT	-1	I	9051242	9051245
Three additional base positions from the 3'-end	Arg_TCG	1	I	9267187	9267190
Three additional base positions from the 3'-end	Pro_AGG	-1	I	9268917	9268920
Three additional base positions from the 3'-end	Gly_TCC	-1	I	9319953	9319956
Three additional base positions from the 3'-end	Gly_TCC	1	I	9320241	9320244
Three additional base positions from the 3'-end	Gly_TCC	-1	I	9327412	9327415
Three additional base positions from the 3'-end	Gly_TCC	1	I	9327700	9327703
Three additional base positions from the 3'-end	Gly_TCC	-1	I	9328404	9328407
Three additional base positions from the 3'-end	Gly_TCC	1	I	9328693	9328696
Three additional base positions from the 3'-end	Gln_TTG	1	I	9558866	9558869
Three additional base positions from the 3'-end	Ile_TAT	1	I	9562429	9562432
Three additional base positions from the 3'-end	Ile_TAT	-1	I	9563831	9563834
Three additional base positions from the 3'-end	Ser_TGA	1	I	9605829	9605832
Three additional base positions from the 3'-end	Lys_CTT	1	I	9679709	9679712
Three additional base positions from the 3'-end	Glu_TTC	1	I	9883758	9883761
Three additional base positions from the 3'-end	Pro_TGG	-1	I	10133587	10133590
Three additional base positions from the 3'-end	Gly_GCC	1	I	10593196	10593199
Three additional base positions from the 3'-end	Gly_CCC	-1	I	10601118	10601121
Three additional base positions from the 3'-end	Gly_CCC	1	I	10601922	10601925
Three additional base positions from the 3'-end	Gly_CCC	-1	I	10604119	10604122
Three additional base positions from the 3'-end	Asp_GTC	1	I	10807264	10807267
Three additional base positions from the 3'-end	Lys_CTT	1	I	10871683	10871686
Three additional base positions from the 3'-end	Lys_CTT	-1	I	10872678	10872681
Three additional base positions from the 3'-end	Gly_TCC	-1	I	10927402	10927405
Three additional base positions from the 3'-end	Gly_TCC	1	I	10927647	10927650
Three additional base positions from the 3'-end	Phe_GAA	1	I	10945886	10945889
Three additional base positions from the 3'-end	Gly_GCC	1	I	11158689	11158692
Three additional base positions from the 3'-end	Cys_GCA	1	I	11505613	11505616
Three additional base positions from the 3'-end	Lys_CTT	-1	I	11579693	11579696

Three additional base positions from the 3'-end	Lys_CTT	-1	I	11584109	11584112
Three additional base positions from the 3'-end	Lys_CTT	1	I	11584850	11584853
Three additional base positions from the 3'-end	Leu_AAG	-1	I	11585357	11585360
Three additional base positions from the 3'-end	Ser_CGA	-1	I	11803303	11803306
Three additional base positions from the 3'-end	Glu_CTC	-1	I	11977178	11977181
Three additional base positions from the 3'-end	Glu_CTC	1	I	11978054	11978057
Three additional base positions from the 3'-end	Glu_CTC	1	I	11978826	11978829
Three additional base positions from the 3'-end	Val_AAC	1	I	12065928	12065931
Three additional base positions from the 3'-end	Met_CAT	1	I	12142973	12142976
Three additional base positions from the 3'-end	Arg_TCT	1	I	12198666	12198669
Three additional base positions from the 3'-end	Met_CAT	-1	I	12225413	12225416
Three additional base positions from the 3'-end	Met_CAT	1	I	12229371	12229374
Three additional base positions from the 3'-end	Ala_AGC	1	I	12601835	12601838
Three additional base positions from the 3'-end	Leu_AAG	-1	I	12731644	12731647
Three additional base positions from the 3'-end	Val_AAC	-1	I	13156342	13156345
Three additional base positions from the 3'-end	Val_AAC	1	I	13161122	13161125
Three additional base positions from the 3'-end	Lys_CTT	-1	I	13297658	13297661
Three additional base positions from the 3'-end	Thr_TGT	-1	I	13310330	13310333
Three additional base positions from the 3'-end	Gly_TCC	-1	I	13324165	13324168
Three additional base positions from the 3'-end	Gly_TCC	1	I	13325055	13325058
Three additional base positions from the 3'-end	Gly_TCC	-1	I	13331132	13331135
Three additional base positions from the 3'-end	Ser_GCT	1	I	14162917	14162920
Three additional base positions from the 3'-end	Thr_GGT	-1	II	1088447	1088450
Three additional base positions from the 3'-end	Thr_TGT	-1	II	1102056	1102059
Three additional base positions from the 3'-end	Thr_TGT	-1	II	1102363	1102366
Three additional base positions from the 3'-end	Pro_GGG	1	II	1332062	1332065
Three additional base positions from the 3'-end	Thr_GGT	-1	II	1333254	1333257
Three additional base positions from the 3'-end	Thr_AGT	1	II	1334210	1334213
Three additional base positions from the 3'-end	Pro_GGG	1	II	1335816	1335819
Three additional base positions from the 3'-end	Thr_GGT	-1	II	1366399	1366402
Three additional base positions from the 3'-end	Ser_CGA	1	II	1368020	1368023
Three additional base positions from the 3'-end	Thr_GGT	-1	II	1476009	1476012
Three additional base positions from the 3'-end	Thr_GGT	-1	II	1476159	1476162
Three additional base positions from the 3'-end	Thr_GGT	1	II	1478835	1478838
Three additional base positions from the 3'-end	His_ATG	1	II	1481997	1482000
Three additional base positions from the 3'-end	Ser_AGA	1	II	1520123	1520126
Three additional base positions from the 3'-end	Undet_???	1	II	2443121	2443124
Three additional base positions from the 3'-end	Leu_AAG	-1	II	2735795	2735798
Three additional base positions from the 3'-end	Leu_AAG	-1	II	2736277	2736280
Three additional base positions from the 3'-end	Leu_AAG	1	II	2860428	2860431
Three additional base positions from the 3'-end	Leu_AAG	-1	II	2861551	2861554
Three additional base positions from the 3'-end	Gly_GCC	-1	II	3062206	3062209
Three additional base positions from the 3'-end	Gly_GCC	1	II	3062457	3062460
Three additional base positions from the 3'-end	Ser_AGA	1	II	3268074	3268077
Three additional base positions from the 3'-end	Thr_AGT	-1	II	3439218	3439221
Three additional base positions from the 3'-end	Lys_CTT	-1	II	3439393	3439396
Three additional base positions from the 3'-end	Thr_AGT	-1	II	3519085	3519088
Three additional base positions from the 3'-end	Lys_CTT	-1	II	3519249	3519252

Three additional base positions from the 3'-end	His_GTG	-1	II	3521486	3521489
Three additional base positions from the 3'-end	Thr_AGT	-1	II	3567609	3567612
Three additional base positions from the 3'-end	Lys_CTT	-1	II	3567763	3567766
Three additional base positions from the 3'-end	Ala_AGC	-1	II	4565009	4565012
Three additional base positions from the 3'-end	Ala_AGC	1	II	4565457	4565460
Three additional base positions from the 3'-end	Ile_AAT	1	II	5032835	5032838
Three additional base positions from the 3'-end	Ile_AAT	-1	II	5057435	5057438
Three additional base positions from the 3'-end	Ile_AAT	1	II	5237922	5237925
Three additional base positions from the 3'-end	Ala_AGC	1	II	5292386	5292389
Three additional base positions from the 3'-end	Glu_CTC	1	II	5293506	5293509
Three additional base positions from the 3'-end	Val_TAC	-1	II	5577179	5577182
Three additional base positions from the 3'-end	Leu_TAA	-1	II	5772892	5772895
Three additional base positions from the 3'-end	Gly_GCC	-1	II	5782188	5782191
Three additional base positions from the 3'-end	Glu_TTC	-1	II	6358331	6358334
Three additional base positions from the 3'-end	Lys_TTT	1	II	6565288	6565291
Three additional base positions from the 3'-end	Leu_TAA	1	II	6721811	6721814
Three additional base positions from the 3'-end	Leu_TAG	1	II	6853242	6853245
Three additional base positions from the 3'-end	Glu_TTC	1	II	6898221	6898224
Three additional base positions from the 3'-end	Ala_TGC	1	II	7001656	7001659
Three additional base positions from the 3'-end	Ala_AGC	-1	II	7002947	7002950
Three additional base positions from the 3'-end	Thr_AGT	1	II	7003302	7003305
Three additional base positions from the 3'-end	Gln_TTG	-1	II	7756896	7756899
Three additional base positions from the 3'-end	Leu_TAA	-1	II	8631903	8631906
Three additional base positions from the 3'-end	Tyr_GTA	-1	II	9211740	9211743
Three additional base positions from the 3'-end	His_GTG	1	II	9404373	9404376
Three additional base positions from the 3'-end	Arg_TCG	1	II	9566254	9566257
Three additional base positions from the 3'-end	Phe_GAA	-1	II	9713161	9713164
Three additional base positions from the 3'-end	Lys_CTT	1	II	9773669	9773672
Three additional base positions from the 3'-end	Pro_TGG	-1	II	10302095	10302098
Three additional base positions from the 3'-end	Pro_TGG	1	II	10302410	10302413
Three additional base positions from the 3'-end	Pro_TGG	-1	II	10304707	10304710
Three additional base positions from the 3'-end	Pro_TGG	1	II	10305022	10305025
Three additional base positions from the 3'-end	Lys_CTT	-1	II	11157740	11157743
Three additional base positions from the 3'-end	Ala_TGC	-1	II	11333945	11333948
Three additional base positions from the 3'-end	Ala_TGC	1	II	11334515	11334518
Three additional base positions from the 3'-end	Lys_CTT	1	II	11441200	11441203
Three additional base positions from the 3'-end	Lys_CTT	-1	II	11442182	11442185
Three additional base positions from the 3'-end	Val_TAC	1	II	11959384	11959387
Three additional base positions from the 3'-end	Ser_GCT	-1	II	12345812	12345815
Three additional base positions from the 3'-end	His_ATG	-1	II	12349568	12349571
Three additional base positions from the 3'-end	Ile_AAT	1	II	12362458	12362461
Three additional base positions from the 3'-end	Met_CAT	-1	II	12435512	12435515
Three additional base positions from the 3'-end	Ile_AAT	1	II	12494750	12494753
Three additional base positions from the 3'-end	Trp_CCA	1	II	12539604	12539607
Three additional base positions from the 3'-end	Lys_TTT	-1	II	12541620	12541623
Three additional base positions from the 3'-end	Leu_AAG	-1	II	12541862	12541865
Three additional base positions from the 3'-end	Ala_AGC	1	II	12542641	12542644
Three additional base positions from the 3'-end	Undet_???	-1	II	12666089	12666092

Three additional base positions from the 3'-end	His_GTG	-1	II	12669924	12669927
Three additional base positions from the 3'-end	Ala_TGC	1	II	12678316	12678319
Three additional base positions from the 3'-end	Arg_CCG	-1	II	12728681	12728684
Three additional base positions from the 3'-end	Gln_TTG	-1	II	14474763	14474766
Three additional base positions from the 3'-end	Asn_GTT	1	II	14617075	14617078
Three additional base positions from the 3'-end	Leu_AAG	-1	II	14635256	14635259
Three additional base positions from the 3'-end	Tyr_GTA	1	III	535463	535466
Three additional base positions from the 3'-end	Gly_GCC	1	III	1040736	1040739
Three additional base positions from the 3'-end	Gly_GCC	1	III	1041613	1041616
Three additional base positions from the 3'-end	Gly_GCC	1	III	1042646	1042649
Three additional base positions from the 3'-end	Undet_???	1	III	1045057	1045060
Three additional base positions from the 3'-end	Pro_TGG	1	III	1047002	1047005
Three additional base positions from the 3'-end	Gly_GCC	1	III	1047891	1047894
Three additional base positions from the 3'-end	Ile_AAT	-1	III	1163341	1163344
Three additional base positions from the 3'-end	Ile_AAT	-1	III	1164787	1164790
Three additional base positions from the 3'-end	Ile_AAT	-1	III	1218364	1218367
Three additional base positions from the 3'-end	Gly_TCC	1	III	1424554	1424557
Three additional base positions from the 3'-end	Gln_TTG	-1	III	1575321	1575324
Three additional base positions from the 3'-end	Arg_TCG	1	III	1575494	1575497
Three additional base positions from the 3'-end	Gln_TTG	-1	III	1575763	1575766
Three additional base positions from the 3'-end	Arg_TCG	1	III	1575936	1575939
Three additional base positions from the 3'-end	Gln_TTG	-1	III	1576996	1576999
Three additional base positions from the 3'-end	Ser_CGA	1	III	1577165	1577168
Three additional base positions from the 3'-end	Ser_CGA	-1	III	1577306	1577309
Three additional base positions from the 3'-end	Ser_CGA	1	III	1577474	1577477
Three additional base positions from the 3'-end	Met_CAT	1	III	1604644	1604647
Three additional base positions from the 3'-end	Glu_CTC	-1	III	1604689	1604692
Three additional base positions from the 3'-end	Arg_TCG	-1	III	1743236	1743239
Three additional base positions from the 3'-end	Arg_GCG	1	III	1998147	1998150
Three additional base positions from the 3'-end	Glu_CTC	1	III	2032579	2032582
Three additional base positions from the 3'-end	Glu_CTC	-1	III	2032883	2032886
Three additional base positions from the 3'-end	Pro_TGG	1	III	2711774	2711777
Three additional base positions from the 3'-end	His_ATG	1	III	2879980	2879983
Three additional base positions from the 3'-end	Leu_AAG	1	III	3099637	3099640
Three additional base positions from the 3'-end	Leu_GAG	1	III	3101148	3101151
Three additional base positions from the 3'-end	Leu_GAG	1	III	3103077	3103080
Three additional base positions from the 3'-end	Leu_GAG	-1	III	3104050	3104053
Three additional base positions from the 3'-end	Pro_GGG	1	III	3105206	3105209
Three additional base positions from the 3'-end	Leu_GAG	-1	III	3106135	3106138
Three additional base positions from the 3'-end	Leu_GAG	-1	III	3108249	3108252
Three additional base positions from the 3'-end	Pro_GGG	1	III	3110862	3110865
Three additional base positions from the 3'-end	Leu_GAG	-1	III	3112132	3112135
Three additional base positions from the 3'-end	Leu_GAG	-1	III	3186977	3186980
Three additional base positions from the 3'-end	Leu_GAG	1	III	3189733	3189736
Three additional base positions from the 3'-end	Asp_GTC	1	III	3191711	3191714
Three additional base positions from the 3'-end	Tyr_GTA	1	III	3195511	3195514
Three additional base positions from the 3'-end	Arg_ACG	-1	III	3339461	3339464
Three additional base positions from the 3'-end	Lys_CTT	1	III	3389599	3389602

Three additional base positions from the 3'-end	Lys_CTT	-1	III	3426899	3426902
Three additional base positions from the 3'-end	Leu_CAA	1	III	4362615	4362618
Three additional base positions from the 3'-end	Ala_AGC	-1	III	4428921	4428924
Three additional base positions from the 3'-end	Arg_ACG	-1	III	4449498	4449501
Three additional base positions from the 3'-end	Arg_ACG	-1	III	4450289	4450292
Three additional base positions from the 3'-end	Arg_TCG	-1	III	4470851	4470854
Three additional base positions from the 3'-end	Met_CAT	-1	III	5294936	5294939
Three additional base positions from the 3'-end	Thr_TGT	1	III	5305937	5305940
Three additional base positions from the 3'-end	Gly_GCC	-1	III	5762006	5762009
Three additional base positions from the 3'-end	Gly_GCC	1	III	5762309	5762312
Three additional base positions from the 3'-end	Leu_AAG	1	III	5769940	5769943
Three additional base positions from the 3'-end	Gly_GCC	-1	III	5782187	5782190
Three additional base positions from the 3'-end	Ser_GCT	-1	III	6056244	6056247
Three additional base positions from the 3'-end	Ser_AGA	1	III	6158807	6158810
Three additional base positions from the 3'-end	Ser_CGA	1	III	6158998	6159001
Three additional base positions from the 3'-end	Ser_CGA	-1	III	6513880	6513883
Three additional base positions from the 3'-end	Lys_CTT	-1	III	6724076	6724079
Three additional base positions from the 3'-end	Arg_CCT	-1	III	6726932	6726935
Three additional base positions from the 3'-end	Asn_GTT	1	III	6805585	6805588
Three additional base positions from the 3'-end	Lys_TTT	-1	III	6887670	6887673
Three additional base positions from the 3'-end	Gly_TCC	-1	III	6888552	6888555
Three additional base positions from the 3'-end	Gly_TCC	1	III	6888823	6888826
Three additional base positions from the 3'-end	Thr_AGT	-1	III	7030496	7030499
Three additional base positions from the 3'-end	Glu_CTC	-1	III	7642089	7642092
Three additional base positions from the 3'-end	Phe_GAA	-1	III	7978402	7978405
Three additional base positions from the 3'-end	Pro_TGG	1	III	8069411	8069414
Three additional base positions from the 3'-end	Met_CAT	1	III	8106659	8106662
Three additional base positions from the 3'-end	Leu_CAA	1	III	8109658	8109661
Three additional base positions from the 3'-end	Lys_CTT	1	III	8473468	8473471
Three additional base positions from the 3'-end	Leu_CAA	1	III	8639345	8639348
Three additional base positions from the 3'-end	Ser_AGA	1	III	8646168	8646171
Three additional base positions from the 3'-end	Pro_CGG	-1	III	8650297	8650300
Three additional base positions from the 3'-end	Phe_GAA	1	III	8653046	8653049
Three additional base positions from the 3'-end	Phe_GAA	-1	III	8653523	8653526
Three additional base positions from the 3'-end	Trp_CCA	-1	III	8678412	8678415
Three additional base positions from the 3'-end	Asn_GTT	-1	III	9104126	9104129
Three additional base positions from the 3'-end	Ser_CGA	-1	III	9861516	9861519
Three additional base positions from the 3'-end	Ser_AGA	-1	III	9861931	9861934
Three additional base positions from the 3'-end	Thr_AGT	-1	III	10624639	10624642
Three additional base positions from the 3'-end	Arg_GCG	-1	III	10961747	10961750
Three additional base positions from the 3'-end	Pro_TGG	-1	III	11066745	11066748
Three additional base positions from the 3'-end	Pro_TGG	1	III	11066999	11067002
Three additional base positions from the 3'-end	Val_AAC	1	III	11353001	11353004
Three additional base positions from the 3'-end	Val_AAC	1	III	11353600	11353603
Three additional base positions from the 3'-end	Arg_TCT	1	III	11474434	11474437
Three additional base positions from the 3'-end	Phe_GAA	1	III	11554797	11554800
Three additional base positions from the 3'-end	Thr_CGT	1	III	11792753	11792756
Three additional base positions from the 3'-end	Gln_TTG	-1	III	12399804	12399807

Three additional base positions from the 3'-end	Ser_GCT	1	III	13009403	13009406
Three additional base positions from the 3'-end	Thr_CGT	-1	III	13016002	13016005
Three additional base positions from the 3'-end	Tyr_GTA	1	III	13224548	13224551
Three additional base positions from the 3'-end	Asn_GTT	-1	III	13225057	13225060
Three additional base positions from the 3'-end	Thr_TGT	-1	III	13411261	13411264
Three additional base positions from the 3'-end	Lys_CTT	1	III	13522252	13522255
Three additional base positions from the 3'-end	Lys_CTT	-1	III	13523235	13523238
Three additional base positions from the 3'-end	Met_CAT	1	IV	67052	67055
Three additional base positions from the 3'-end	Arg_CCT	1	IV	144054	144057
Three additional base positions from the 3'-end	Asp_GTC	-1	IV	322628	322631
Three additional base positions from the 3'-end	Val_CAC	1	IV	620554	620557
Three additional base positions from the 3'-end	SeC(e)_TCA	1	IV	658266	658269
Three additional base positions from the 3'-end	Asp_GTC	1	IV	683134	683137
Three additional base positions from the 3'-end	Glu_TTC	1	IV	842880	842883
Three additional base positions from the 3'-end	Ala_GGC	-1	IV	1144680	1144683
Three additional base positions from the 3'-end	His_ATG	-1	IV	1146070	1146073
Three additional base positions from the 3'-end	Undet_???	-1	IV	1355409	1355412
Three additional base positions from the 3'-end	Arg_TCG	1	IV	1365261	1365264
Three additional base positions from the 3'-end	Undet_???	-1	IV	1385618	1385621
Three additional base positions from the 3'-end	Glu_TTC	-1	IV	1388211	1388214
Three additional base positions from the 3'-end	Tyr_ATA	1	IV	1407334	1407337
Three additional base positions from the 3'-end	Undet_???	1	IV	1464752	1464755
Three additional base positions from the 3'-end	His_GTG	-1	IV	1474647	1474650
Three additional base positions from the 3'-end	Asp_GTC	1	IV	1514662	1514665
Three additional base positions from the 3'-end	Glu_TTC	-1	IV	2639803	2639806
Three additional base positions from the 3'-end	Gln_TTG	-1	IV	2659007	2659010
Three additional base positions from the 3'-end	Gly_TCC	1	IV	2664312	2664315
Three additional base positions from the 3'-end	Undet_???	1	IV	2740284	2740287
Three additional base positions from the 3'-end	Asp_GTC	1	IV	2800168	2800171
Three additional base positions from the 3'-end	Asp_GTC	-1	IV	2801145	2801148
Three additional base positions from the 3'-end	Asp_GTC	1	IV	2802406	2802409
Three additional base positions from the 3'-end	Asp_GTC	1	IV	2805696	2805699
Three additional base positions from the 3'-end	Asp_GTC	1	IV	2808424	2808427
Three additional base positions from the 3'-end	Lys_CTT	-1	IV	3501640	3501643
Three additional base positions from the 3'-end	Arg_ACG	1	IV	3524887	3524890
Three additional base positions from the 3'-end	Pro_CGG	-1	IV	3552181	3552184
Three additional base positions from the 3'-end	Lys_CTT	1	IV	3589330	3589333
Three additional base positions from the 3'-end	Lys_CTT	-1	IV	3590282	3590285
Three additional base positions from the 3'-end	Trp_CCA	-1	IV	3681871	3681874
Three additional base positions from the 3'-end	Pro_GGG	-1	IV	4106563	4106566
Three additional base positions from the 3'-end	His_GTG	1	IV	4861815	4861818
Three additional base positions from the 3'-end	Lys_TTT	1	IV	5038984	5038987
Three additional base positions from the 3'-end	Gln_CTG	1	IV	5334775	5334778
Three additional base positions from the 3'-end	Trp_CCA	-1	IV	5344288	5344291
Three additional base positions from the 3'-end	Lys_CTT	1	IV	5349413	5349416
Three additional base positions from the 3'-end	Lys_CTT	1	IV	5967220	5967223
Three additional base positions from the 3'-end	Val_CAC	1	IV	6481222	6481225
Three additional base positions from the 3'-end	Leu_CAG	1	IV	6556890	6556893

Three additional base positions from the 3'-end	Ala_AGC	1	IV	6562262	6562265
Three additional base positions from the 3'-end	Gln_TTG	1	IV	7275387	7275390
Three additional base positions from the 3'-end	Trp_CCA	-1	IV	7344141	7344144
Three additional base positions from the 3'-end	Asp_GTC	1	IV	8927260	8927263
Three additional base positions from the 3'-end	Thr_TGT	-1	IV	9000785	9000788
Three additional base positions from the 3'-end	Gln_TTG	-1	IV	10311205	10311208
Three additional base positions from the 3'-end	Pro_AGG	1	IV	11231614	11231617
Three additional base positions from the 3'-end	Lys_CTT	1	IV	11362175	11362178
Three additional base positions from the 3'-end	Lys_CTT	-1	IV	11363168	11363171
Three additional base positions from the 3'-end	His_GTG	1	IV	11721213	11721216
Three additional base positions from the 3'-end	Leu_CAA	-1	IV	11928451	11928454
Three additional base positions from the 3'-end	Pro_CGG	-1	IV	12005571	12005574
Three additional base positions from the 3'-end	Gln_CTG	-1	IV	12416915	12416918
Three additional base positions from the 3'-end	Gln_TTG	-1	IV	12417222	12417225
Three additional base positions from the 3'-end	Arg_ACG	-1	IV	12417899	12417902
Three additional base positions from the 3'-end	Lys_CTT	1	IV	12594788	12594791
Three additional base positions from the 3'-end	Lys_CTT	1	IV	13043740	13043743
Three additional base positions from the 3'-end	Lys_CTT	-1	IV	13044432	13044435
Three additional base positions from the 3'-end	His_GTG	1	IV	13870311	13870314
Three additional base positions from the 3'-end	Ala_TGC	-1	IV	14013776	14013779
Three additional base positions from the 3'-end	Pro_TGG	1	IV	14225148	14225151
Three additional base positions from the 3'-end	Gly_TCC	-1	IV	14576590	14576593
Three additional base positions from the 3'-end	Arg_ACG	-1	IV	14611275	14611278
Three additional base positions from the 3'-end	His_GTG	-1	IV	14888986	14888989
Three additional base positions from the 3'-end	His_GTG	1	IV	15187383	15187386
Three additional base positions from the 3'-end	His_GTG	-1	IV	15187618	15187621
Three additional base positions from the 3'-end	Val_AAC	1	IV	15235428	15235431
Three additional base positions from the 3'-end	Trp_CCA	1	IV	15289742	15289745
Three additional base positions from the 3'-end	Ser_AGA	-1	IV	15311082	15311085
Three additional base positions from the 3'-end	Ser_AGA	-1	IV	15318442	15318445
Three additional base positions from the 3'-end	Ala_TGC	-1	IV	15604276	15604279
Three additional base positions from the 3'-end	Lys_CTT	1	IV	15793455	15793458
Three additional base positions from the 3'-end	Asp_GTC	-1	IV	15931306	15931309
Three additional base positions from the 3'-end	Asp_GTC	1	IV	15932049	15932052
Three additional base positions from the 3'-end	Glu_TTC	-1	IV	15933125	15933128
Three additional base positions from the 3'-end	Glu_TTC	1	IV	15933392	15933395
Three additional base positions from the 3'-end	Val_AAC	-1	IV	16382395	16382398
Three additional base positions from the 3'-end	His_GTG	1	IV	16388495	16388498
Three additional base positions from the 3'-end	Ala_AGC	-1	IV	16389175	16389178
Three additional base positions from the 3'-end	Ala_AGC	1	IV	16398423	16398426
Three additional base positions from the 3'-end	Ala_AGC	1	IV	16399223	16399226
Three additional base positions from the 3'-end	Val_AAC	1	IV	16400170	16400173
Three additional base positions from the 3'-end	His_GTG	1	IV	16400935	16400938
Three additional base positions from the 3'-end	Lys_TTT	1	IV	16552259	16552262
Three additional base positions from the 3'-end	Lys_TTT	-1	IV	16552806	16552809
Three additional base positions from the 3'-end	Ser_TGA	1	IV	16577354	16577357
Three additional base positions from the 3'-end	Ser_TGA	-1	IV	16583705	16583708
Three additional base positions from the 3'-end	Gly_TCC	-1	IV	16670829	16670832

Three additional base positions from the 3'-end	Gly_TCC	1	IV	16682045	16682048
Three additional base positions from the 3'-end	Gly_TCC	-1	IV	16684346	16684349
Three additional base positions from the 3'-end	Gly_TCC	1	IV	16775220	16775223
Three additional base positions from the 3'-end	Gly_TCC	1	IV	16776126	16776129
Three additional base positions from the 3'-end	Ala_AGC	1	V	857397	857400
Three additional base positions from the 3'-end	Undet_???	-1	V	2149172	2149175
Three additional base positions from the 3'-end	Thr_AGT	-1	V	2664661	2664664
Three additional base positions from the 3'-end	Thr_AGT	1	V	2674079	2674082
Three additional base positions from the 3'-end	Met_CAT	-1	V	3455319	3455322
Three additional base positions from the 3'-end	Pro_TGG	-1	V	3582124	3582127
Three additional base positions from the 3'-end	Pro_TGG	1	V	3582388	3582391
Three additional base positions from the 3'-end	Pro_TGG	-1	V	3588297	3588300
Three additional base positions from the 3'-end	Pro_TGG	1	V	3588561	3588564
Three additional base positions from the 3'-end	Gly_GCC	1	V	4310666	4310669
Three additional base positions from the 3'-end	Lys_CTT	-1	V	4782211	4782214
Three additional base positions from the 3'-end	Gln_TTG	-1	V	5330119	5330122
Three additional base positions from the 3'-end	Cys_GCA	1	V	6335452	6335455
Three additional base positions from the 3'-end	Lys_CTT	1	V	6418030	6418033
Three additional base positions from the 3'-end	Lys_CTT	-1	V	6421705	6421708
Three additional base positions from the 3'-end	Ser_GCT	-1	V	6551833	6551836
Three additional base positions from the 3'-end	Cys_GCA	1	V	6559811	6559814
Three additional base positions from the 3'-end	Ile_TAT	1	V	6917532	6917535
Three additional base positions from the 3'-end	Glu_TTC	-1	V	7069725	7069728
Three additional base positions from the 3'-end	Arg_TCT	1	V	7506448	7506451
Three additional base positions from the 3'-end	Thr_CGT	-1	V	7730631	7730634
Three additional base positions from the 3'-end	Met_CAT	-1	V	8230182	8230185
Three additional base positions from the 3'-end	Leu_TAG	-1	V	8235434	8235437
Three additional base positions from the 3'-end	Tyr_GTA	1	V	8247544	8247547
Three additional base positions from the 3'-end	Lys_TTT	1	V	8495353	8495356
Three additional base positions from the 3'-end	Thr_AGT	-1	V	8496079	8496082
Three additional base positions from the 3'-end	Lys_TTT	-1	V	8499539	8499542
Three additional base positions from the 3'-end	Ala_CGC	1	V	9318249	9318252
Three additional base positions from the 3'-end	Thr_AGT	-1	V	9407012	9407015
Three additional base positions from the 3'-end	Tyr_GTA	-1	V	9412177	9412180
Three additional base positions from the 3'-end	Ala_CGC	1	V	9503609	9503612
Three additional base positions from the 3'-end	Leu_CAG	-1	V	11174768	11174771
Three additional base positions from the 3'-end	Leu_CAG	-1	V	11183644	11183647
Three additional base positions from the 3'-end	Val_CAC	-1	V	11412213	11412216
Three additional base positions from the 3'-end	Ile_TAT	1	V	12002241	12002244
Three additional base positions from the 3'-end	Asn_GTT	-1	V	12331942	12331945
Three additional base positions from the 3'-end	Val_TAC	-1	V	12683330	12683333
Three additional base positions from the 3'-end	Ser_TGA	-1	V	12780365	12780368
Three additional base positions from the 3'-end	Ala_AGC	-1	V	13003490	13003493
Three additional base positions from the 3'-end	Lys_TTT	-1	V	13027009	13027012
Three additional base positions from the 3'-end	Pro_TGG	-1	V	13507186	13507189
Three additional base positions from the 3'-end	Pro_TGG	1	V	13509751	13509754
Three additional base positions from the 3'-end	Gln_CTG	1	V	14231420	14231423
Three additional base positions from the 3'-end	Tyr_GTA	1	V	14689600	14689603

Three additional base positions from the 3'-end	Cys_GCA	-1	V	14855768	14855771
Three additional base positions from the 3'-end	Phe_GAA	-1	V	14876370	14876373
Three additional base positions from the 3'-end	Val_AAC	-1	V	15013013	15013016
Three additional base positions from the 3'-end	Asn_GTT	1	V	15156737	15156740
Three additional base positions from the 3'-end	Lys_TTT	1	V	15465140	15465143
Three additional base positions from the 3'-end	Pro_TGG	-1	V	15465203	15465206
Three additional base positions from the 3'-end	Ala_CGC	-1	V	15479963	15479966
Three additional base positions from the 3'-end	Tyr_GTA	1	V	15480759	15480762
Three additional base positions from the 3'-end	Gly_GCC	-1	V	15505186	15505189
Three additional base positions from the 3'-end	Val_CAC	-1	V	15515484	15515487
Three additional base positions from the 3'-end	Met_CAT	1	V	15517450	15517453
Three additional base positions from the 3'-end	Ser_TGA	-1	V	15547852	15547855
Three additional base positions from the 3'-end	Ser_TGA	1	V	15550674	15550677
Three additional base positions from the 3'-end	Asp_GTC	-1	V	15551776	15551779
Three additional base positions from the 3'-end	His_GTG	1	V	15554082	15554085
Three additional base positions from the 3'-end	Thr_CGT	1	V	16219210	16219213
Three additional base positions from the 3'-end	Asn_GTT	-1	V	16338346	16338349
Three additional base positions from the 3'-end	Asn_GTT	1	V	16338600	16338603
Three additional base positions from the 3'-end	Asn_GTT	-1	V	16341577	16341580
Three additional base positions from the 3'-end	Asn_GTT	1	V	16341831	16341834
Three additional base positions from the 3'-end	Lys_CTT	1	V	16564113	16564116
Three additional base positions from the 3'-end	Lys_CTT	-1	V	16565108	16565111
Three additional base positions from the 3'-end	Gln_CTG	-1	V	16592212	16592215
Three additional base positions from the 3'-end	Lys_TTT	-1	V	16622034	16622037
Three additional base positions from the 3'-end	Asp_GTC	1	V	16640628	16640631
Three additional base positions from the 3'-end	Asp_GTC	-1	V	16645681	16645684
Three additional base positions from the 3'-end	Asp_GTC	-1	V	16648741	16648744
Three additional base positions from the 3'-end	Tyr_GTA	-1	V	17317850	17317853
Three additional base positions from the 3'-end	Tyr_GTA	-1	V	17542416	17542419
Three additional base positions from the 3'-end	His_ATG	-1	V	17660748	17660751
Three additional base positions from the 3'-end	Gln_TTG	1	V	17660930	17660933
Three additional base positions from the 3'-end	Arg_ACG	1	V	17662217	17662220
Three additional base positions from the 3'-end	Lys_TTT	-1	V	17668330	17668333
Three additional base positions from the 3'-end	His_ATG	1	V	17686426	17686429
Three additional base positions from the 3'-end	His_GTG	1	V	17686701	17686704
Three additional base positions from the 3'-end	Undet_???	-1	V	17688404	17688407
Three additional base positions from the 3'-end	His_ATG	1	V	17690553	17690556
Three additional base positions from the 3'-end	Leu_GAG	1	V	17691328	17691331
Three additional base positions from the 3'-end	Gln_TTG	-1	V	17693769	17693772
Three additional base positions from the 3'-end	Undet_???	-1	V	17694064	17694067
Three additional base positions from the 3'-end	Sup_TTA	-1	V	17734605	17734608
Three additional base positions from the 3'-end	Gln_TTG	1	V	17734747	17734750
Three additional base positions from the 3'-end	Sup_TTA	-1	V	17739460	17739463
Three additional base positions from the 3'-end	Gln_TTG	1	V	17739602	17739605
Three additional base positions from the 3'-end	His_ATG	-1	V	17765654	17765657
Three additional base positions from the 3'-end	Thr_GGT	1	V	17783204	17783207
Three additional base positions from the 3'-end	Ile_GAT	-1	V	17784485	17784488
Three additional base positions from the 3'-end	Thr_GGT	-1	V	17785684	17785687

Three additional base positions from the 3'-end	Thr_AGT	-1	V	17786856	17786859
Three additional base positions from the 3'-end	Gly_ACC	1	V	17930395	17930398
Three additional base positions from the 3'-end	Gly_CCC	1	V	17930839	17930842
Three additional base positions from the 3'-end	Gly_GCC	1	V	17931120	17931123
Three additional base positions from the 3'-end	Gly_GCC	1	V	17931738	17931741
Three additional base positions from the 3'-end	Gly_ACC	1	V	17932805	17932808
Three additional base positions from the 3'-end	Gly_GCC	1	V	17933375	17933378
Three additional base positions from the 3'-end	His_ATG	1	V	18367182	18367185
Three additional base positions from the 3'-end	His_ATG	1	V	18371273	18371276
Three additional base positions from the 3'-end	Undet_???	-1	V	18372849	18372852
Three additional base positions from the 3'-end	Undet_???	-1	V	18373634	18373637
Three additional base positions from the 3'-end	Ile_TAT	-1	V	18373931	18373934
Three additional base positions from the 3'-end	Thr_TGT	-1	V	18412451	18412454
Three additional base positions from the 3'-end	Arg_TCG	1	V	18461870	18461873
Three additional base positions from the 3'-end	Leu_CAA	1	V	18622796	18622799
Three additional base positions from the 3'-end	Gly_ACC	-1	V	18801942	18801945
Three additional base positions from the 3'-end	Ser_CGA	-1	V	18804677	18804680
Three additional base positions from the 3'-end	Ala_GGC	-1	V	18805533	18805536
Three additional base positions from the 3'-end	Undet_???	1	V	18825423	18825426
Three additional base positions from the 3'-end	Gly_ACC	1	V	18826028	18826031
Three additional base positions from the 3'-end	Gly_ACC	-1	V	18827843	18827846
Three additional base positions from the 3'-end	Gly_ACC	-1	V	18829015	18829018
Three additional base positions from the 3'-end	Undet_???	-1	V	18906263	18906266
Three additional base positions from the 3'-end	Thr_GGT	-1	V	19057472	19057475
Three additional base positions from the 3'-end	Thr_GGT	1	V	19058334	19058337
Three additional base positions from the 3'-end	Thr_AGT	-1	V	19058969	19058972
Three additional base positions from the 3'-end	Thr_GGT	-1	V	19059253	19059256
Three additional base positions from the 3'-end	Thr_GGT	-1	V	19059561	19059564
Three additional base positions from the 3'-end	Thr_GGT	-1	V	19060476	19060479
Three additional base positions from the 3'-end	Pro_GGG	1	V	19082984	19082987
Three additional base positions from the 3'-end	Pro_GGG	1	V	19084647	19084650
Three additional base positions from the 3'-end	Phe_GAA	1	V	19126087	19126090
Three additional base positions from the 3'-end	Glu_TTC	1	V	19142830	19142833
Three additional base positions from the 3'-end	Val_GAC	1	V	19347625	19347628
Three additional base positions from the 3'-end	His_ATG	1	V	19393563	19393566
Three additional base positions from the 3'-end	His_ATG	-1	V	19394748	19394751
Three additional base positions from the 3'-end	His_ATG	-1	V	19396662	19396665
Three additional base positions from the 3'-end	His_ATG	-1	V	19396956	19396959
Three additional base positions from the 3'-end	His_ATG	1	V	19398629	19398632
Three additional base positions from the 3'-end	Gln_TTG	1	V	19431334	19431337
Three additional base positions from the 3'-end	Undet_???	-1	V	19557605	19557608
Three additional base positions from the 3'-end	Undet_???	-1	V	19557913	19557916
Three additional base positions from the 3'-end	Undet_???	-1	V	19558995	19558998
Three additional base positions from the 3'-end	His_ATG	1	V	19577431	19577434
Three additional base positions from the 3'-end	His_ATG	-1	V	19578431	19578434
Three additional base positions from the 3'-end	His_ATG	-1	V	19578736	19578739
Three additional base positions from the 3'-end	Undet_???	1	V	19579748	19579751
Three additional base positions from the 3'-end	Undet_???	1	V	19581754	19581757

Three additional base positions from the 3'-end	Undet_???	1	V	19582043	19582046
Three additional base positions from the 3'-end	His_GTG	1	V	19584867	19584870
Three additional base positions from the 3'-end	Gln_CTG	-1	V	19587230	19587233
Three additional base positions from the 3'-end	His_ATG	-1	V	19594007	19594010
Three additional base positions from the 3'-end	Gln_TTG	-1	V	19594471	19594474
Three additional base positions from the 3'-end	His_ATG	-1	V	19594771	19594774
Three additional base positions from the 3'-end	His_ATG	-1	V	19595553	19595556
Three additional base positions from the 3'-end	Undet_???	-1	V	19595832	19595835
Three additional base positions from the 3'-end	Undet_???	1	V	19597008	19597011
Three additional base positions from the 3'-end	Pro_GGG	-1	V	19598565	19598568
Three additional base positions from the 3'-end	His_GTG	1	V	19599558	19599561
Three additional base positions from the 3'-end	His_ATG	-1	V	19624009	19624012
Three additional base positions from the 3'-end	Asp_ATC	1	V	19629869	19629872
Three additional base positions from the 3'-end	His_GTG	1	V	19656246	19656249
Three additional base positions from the 3'-end	Gln_TTG	-1	V	19665296	19665299
Three additional base positions from the 3'-end	Pro_TGG	-1	V	19665914	19665917
Three additional base positions from the 3'-end	Pro_GGG	1	V	19667945	19667948
Three additional base positions from the 3'-end	Asn_GTT	1	V	19723073	19723076
Three additional base positions from the 3'-end	His_ATG	-1	V	20016540	20016543
Three additional base positions from the 3'-end	His_ATG	-1	V	20021056	20021059
Three additional base positions from the 3'-end	Glu_TTC	1	V	20123272	20123275
Three additional base positions from the 3'-end	Gln_TTG	-1	V	20123699	20123702
Three additional base positions from the 3'-end	Gln_TTG	1	V	20123886	20123889
Three additional base positions from the 3'-end	Gln_TTG	-1	V	20126135	20126138
Three additional base positions from the 3'-end	Sup_TTA	1	V	20135044	20135047
Three additional base positions from the 3'-end	Gln_TTG	-1	V	20214830	20214833
Three additional base positions from the 3'-end	His_ATG	1	V	20219339	20219342
Three additional base positions from the 3'-end	Asn_GTT	-1	V	20347762	20347765
Three additional base positions from the 3'-end	Asn_GTT	-1	V	20350494	20350497
Three additional base positions from the 3'-end	Cys_GCA	-1	V	20694086	20694089
Three additional base positions from the 3'-end	Glu_CTC	1	X	86958	86961
Three additional base positions from the 3'-end	Lys_CTT	1	X	288521	288524
Three additional base positions from the 3'-end	Lys_CTT	-1	X	289509	289512
Three additional base positions from the 3'-end	Leu_AAG	-1	X	353251	353254
Three additional base positions from the 3'-end	Gln_TTG	1	X	410463	410466
Three additional base positions from the 3'-end	Thr_TGT	1	X	487887	487890
Three additional base positions from the 3'-end	Asp_GTC	-1	X	489865	489868
Three additional base positions from the 3'-end	Tyr_GTA	-1	X	865384	865387
Three additional base positions from the 3'-end	Arg_TCG	-1	X	1089166	1089169
Three additional base positions from the 3'-end	Arg_TCG	1	X	1089471	1089474
Three additional base positions from the 3'-end	Arg_TCG	1	X	1089907	1089910
Three additional base positions from the 3'-end	Gly_TCC	-1	X	1379594	1379597
Three additional base positions from the 3'-end	Undet_???	-1	X	1433054	1433057
Three additional base positions from the 3'-end	Asp_ATC	-1	X	1474007	1474010
Three additional base positions from the 3'-end	Ile_AAT	-1	X	1589658	1589661
Three additional base positions from the 3'-end	Ile_AAT	-1	X	1590071	1590074
Three additional base positions from the 3'-end	Ile_AAT	-1	X	1590499	1590502
Three additional base positions from the 3'-end	Asp_ATC	1	X	1620350	1620353

Three additional base positions from the 3'-end	Arg_CCG	-1	X	1629872	1629875
Three additional base positions from the 3'-end	Asp_ATC	1	X	1643917	1643920
Three additional base positions from the 3'-end	Ser_CGA	-1	X	1691074	1691077
Three additional base positions from the 3'-end	Ser_CGA	1	X	1692796	1692799
Three additional base positions from the 3'-end	Arg_TCG	1	X	1698741	1698744
Three additional base positions from the 3'-end	Arg_CCT	-1	X	1764245	1764248
Three additional base positions from the 3'-end	Arg_CCT	1	X	1775555	1775558
Three additional base positions from the 3'-end	Ile_TAT	1	X	1830741	1830744
Three additional base positions from the 3'-end	Glu_TTC	1	X	1858884	1858887
Three additional base positions from the 3'-end	Pro_CGG	-1	X	1880807	1880810
Three additional base positions from the 3'-end	Leu_TAA	1	X	1891511	1891514
Three additional base positions from the 3'-end	Undet_???	-1	X	2125752	2125755
Three additional base positions from the 3'-end	Asp_GTC	-1	X	2227179	2227182
Three additional base positions from the 3'-end	Lys_CTT	1	X	2462619	2462622
Three additional base positions from the 3'-end	Lys_CTT	-1	X	2463474	2463477
Three additional base positions from the 3'-end	Ile_AAT	-1	X	2526207	2526210
Three additional base positions from the 3'-end	Thr_AGT	1	X	2528106	2528109
Three additional base positions from the 3'-end	Ile_AAT	1	X	2531427	2531430
Three additional base positions from the 3'-end	Asn_GTT	-1	X	2549517	2549520
Three additional base positions from the 3'-end	Asp_GTC	1	X	2553629	2553632
Three additional base positions from the 3'-end	Gly_GCC	1	X	2613305	2613308
Three additional base positions from the 3'-end	Glu_CTC	-1	X	3035962	3035965
Three additional base positions from the 3'-end	Gly_TCC	1	X	3036225	3036228
Three additional base positions from the 3'-end	Thr_AGT	-1	X	3371405	3371408
Three additional base positions from the 3'-end	Met_CAT	1	X	3697815	3697818
Three additional base positions from the 3'-end	Val_TAC	-1	X	3793544	3793547
Three additional base positions from the 3'-end	Pro_TGG	1	X	3969607	3969610
Three additional base positions from the 3'-end	Pro_AGG	1	X	3970147	3970150
Three additional base positions from the 3'-end	Pro_AGG	1	X	3970316	3970319
Three additional base positions from the 3'-end	Gly_TCC	1	X	3994652	3994655
Three additional base positions from the 3'-end	Ile_AAT	1	X	4242575	4242578
Three additional base positions from the 3'-end	Glu_CTC	1	X	4409830	4409833
Three additional base positions from the 3'-end	Glu_CTC	1	X	4410698	4410701
Three additional base positions from the 3'-end	Glu_TTC	1	X	4444308	4444311
Three additional base positions from the 3'-end	Thr_AGT	1	X	4445060	4445063
Three additional base positions from the 3'-end	Lys_TTT	1	X	4789174	4789177
Three additional base positions from the 3'-end	His_GTG	-1	X	5022782	5022785
Three additional base positions from the 3'-end	Ile_AAT	-1	X	5085587	5085590
Three additional base positions from the 3'-end	Lys_CTT	1	X	5284461	5284464
Three additional base positions from the 3'-end	Lys_CTT	-1	X	5285443	5285446
Three additional base positions from the 3'-end	Phe_GAA	-1	X	5319349	5319352
Three additional base positions from the 3'-end	Met_CAT	-1	X	5407377	5407380
Three additional base positions from the 3'-end	Lys_TTT	-1	X	5451168	5451171
Three additional base positions from the 3'-end	Met_CAT	1	X	5485152	5485155
Three additional base positions from the 3'-end	Met_CAT	1	X	5485495	5485498
Three additional base positions from the 3'-end	Met_CAT	1	X	5485786	5485789
Three additional base positions from the 3'-end	Ser_GCT	1	X	5708074	5708077
Three additional base positions from the 3'-end	Ser_GCT	1	X	5709421	5709424

Three additional base positions from the 3'-end	Lys_TTT	1	X	5713689	5713692
Three additional base positions from the 3'-end	Pro_TGG	-1	X	5801172	5801175
Three additional base positions from the 3'-end	Pro_TGG	1	X	5801508	5801511
Three additional base positions from the 3'-end	Arg_ACG	-1	X	6220031	6220034
Three additional base positions from the 3'-end	Ala_TGC	1	X	6286356	6286359
Three additional base positions from the 3'-end	Ala_TGC	-1	X	6292238	6292241
Three additional base positions from the 3'-end	Trp_CCA	1	X	6371413	6371416
Three additional base positions from the 3'-end	Phe_GAA	1	X	6593631	6593634
Three additional base positions from the 3'-end	Lys_CTT	1	X	6600263	6600266
Three additional base positions from the 3'-end	Lys_CTT	-1	X	6601268	6601271
Three additional base positions from the 3'-end	Leu_AAG	-1	X	6903070	6903073
Three additional base positions from the 3'-end	Thr_AGT	-1	X	7176936	7176939
Three additional base positions from the 3'-end	Ala_AGC	1	X	7323778	7323781
Three additional base positions from the 3'-end	Ala_AGC	-1	X	7378734	7378737
Three additional base positions from the 3'-end	Ala_AGC	-1	X	7507241	7507244
Three additional base positions from the 3'-end	Glu_CTC	1	X	7681060	7681063
Three additional base positions from the 3'-end	Asn_GTT	1	X	7773211	7773214
Three additional base positions from the 3'-end	Asn_GTT	1	X	7773739	7773742
Three additional base positions from the 3'-end	Asn_GTT	-1	X	7773910	7773913
Three additional base positions from the 3'-end	Gly_TCC	-1	X	7794281	7794284
Three additional base positions from the 3'-end	Gly_TCC	1	X	7794623	7794626
Three additional base positions from the 3'-end	Glu_CTC	-1	X	7898256	7898259
Three additional base positions from the 3'-end	Glu_CTC	1	X	7906608	7906611
Three additional base positions from the 3'-end	Arg_ACG	-1	X	8036632	8036635
Three additional base positions from the 3'-end	Pro_AGG	1	X	8037529	8037532
Three additional base positions from the 3'-end	Asp_GTC	1	X	8149178	8149181
Three additional base positions from the 3'-end	Asp_GTC	-1	X	8152493	8152496
Three additional base positions from the 3'-end	Ser_AGA	-1	X	8152759	8152762
Three additional base positions from the 3'-end	Ser_AGA	1	X	8154477	8154480
Three additional base positions from the 3'-end	Asp_GTC	1	X	8154744	8154747
Three additional base positions from the 3'-end	Ser_AGA	1	X	8155494	8155497
Three additional base positions from the 3'-end	Asp_GTC	1	X	8155774	8155777
Three additional base positions from the 3'-end	Met_CAT	1	X	8156200	8156203
Three additional base positions from the 3'-end	Ile_AAT	-1	X	8156581	8156584
Three additional base positions from the 3'-end	Arg_TCT	-1	X	8381738	8381741
Three additional base positions from the 3'-end	Arg_TCT	1	X	8386223	8386226
Three additional base positions from the 3'-end	Arg_TCT	1	X	8386633	8386636
Three additional base positions from the 3'-end	Asn_GTT	1	X	8404518	8404521
Three additional base positions from the 3'-end	Leu_AAG	-1	X	8409239	8409242
Three additional base positions from the 3'-end	Leu_AAG	-1	X	8417855	8417858
Three additional base positions from the 3'-end	Leu_AAG	-1	X	8419357	8419360
Three additional base positions from the 3'-end	Lys_CTT	-1	X	8458982	8458985
Three additional base positions from the 3'-end	Gln_CTG	-1	X	8608778	8608781
Three additional base positions from the 3'-end	Gln_TTG	1	X	8609057	8609060
Three additional base positions from the 3'-end	Arg_ACG	1	X	8623623	8623626
Three additional base positions from the 3'-end	Cys_GCA	-1	X	8623708	8623711
Three additional base positions from the 3'-end	Cys_GCA	-1	X	8623942	8623945
Three additional base positions from the 3'-end	Cys_GCA	1	X	8624301	8624304

Three additional base positions from the 3'-end	Arg_ACG	-1	X	8624404	8624407
Three additional base positions from the 3'-end	Cys_GCA	-1	X	8638564	8638567
Three additional base positions from the 3'-end	Cys_GCA	1	X	8638941	8638944
Three additional base positions from the 3'-end	Arg_ACG	-1	X	8639169	8639172
Three additional base positions from the 3'-end	Arg_ACG	-1	X	8640738	8640741
Three additional base positions from the 3'-end	Glu_TTC	1	X	8646499	8646502
Three additional base positions from the 3'-end	Val_AAC	-1	X	8650653	8650656
Three additional base positions from the 3'-end	Gly_GCC	-1	X	8796765	8796768
Three additional base positions from the 3'-end	Cys_GCA	-1	X	8818043	8818046
Three additional base positions from the 3'-end	Gly_TCC	-1	X	8818521	8818524
Three additional base positions from the 3'-end	Gly_TCC	1	X	8819699	8819702
Three additional base positions from the 3'-end	Gly_TCC	-1	X	8819867	8819870
Three additional base positions from the 3'-end	Gly_TCC	1	X	8820203	8820206
Three additional base positions from the 3'-end	Glu_TTC	1	X	8836131	8836134
Three additional base positions from the 3'-end	Gly_TCC	-1	X	8838281	8838284
Three additional base positions from the 3'-end	Gly_TCC	-1	X	8846449	8846452
Three additional base positions from the 3'-end	Gly_TCC	1	X	8846739	8846742
Three additional base positions from the 3'-end	Trp_CCA	1	X	8877358	8877361
Three additional base positions from the 3'-end	Gln_TTG	1	X	8925659	8925662
Three additional base positions from the 3'-end	Leu_AAG	1	X	8939577	8939580
Three additional base positions from the 3'-end	Trp_CCA	-1	X	8944174	8944177
Three additional base positions from the 3'-end	Trp_CCA	-1	X	8945102	8945105
Three additional base positions from the 3'-end	Leu_AAG	-1	X	8955862	8955865
Three additional base positions from the 3'-end	Pro_TGG	-1	X	8969430	8969433
Three additional base positions from the 3'-end	Pro_TGG	1	X	8969755	8969758
Three additional base positions from the 3'-end	Ser_AGA	1	X	8971627	8971630
Three additional base positions from the 3'-end	Thr_TGT	-1	X	9024354	9024357
Three additional base positions from the 3'-end	Thr_CGT	-1	X	9025604	9025607
Three additional base positions from the 3'-end	Undet_???	-1	X	9059221	9059224
Three additional base positions from the 3'-end	Gln_TTG	-1	X	9074613	9074616
Three additional base positions from the 3'-end	Gln_TTG	-1	X	9078195	9078198
Three additional base positions from the 3'-end	Gln_TTG	-1	X	9090567	9090570
Three additional base positions from the 3'-end	Gln_CTG	1	X	9090845	9090848
Three additional base positions from the 3'-end	Gln_TTG	-1	X	9091037	9091040
Three additional base positions from the 3'-end	Gln_TTG	1	X	9091325	9091328
Three additional base positions from the 3'-end	Gln_TTG	-1	X	9091497	9091500
Three additional base positions from the 3'-end	Ala_AGC	1	X	9237823	9237826
Three additional base positions from the 3'-end	Ala_AGC	1	X	9238876	9238879
Three additional base positions from the 3'-end	Met_CAT	-1	X	9373087	9373090
Three additional base positions from the 3'-end	Pro_TGG	-1	X	9467666	9467669
Three additional base positions from the 3'-end	Val_AAC	-1	X	9561027	9561030
Three additional base positions from the 3'-end	Ala_AGC	-1	X	9622763	9622766
Three additional base positions from the 3'-end	Ala_AGC	-1	X	9637791	9637794
Three additional base positions from the 3'-end	Ala_TGC	1	X	9743602	9743605
Three additional base positions from the 3'-end	Ala_AGC	1	X	9743760	9743763
Three additional base positions from the 3'-end	Ile_TAT	-1	X	9829605	9829608
Three additional base positions from the 3'-end	Arg_ACG	-1	X	10018363	10018366
Three additional base positions from the 3'-end	Ser_TGA	1	X	10124520	10124523

Three additional base positions from the 3'-end	Arg_ACG	1	X	10132691	10132694
Three additional base positions from the 3'-end	Arg_ACG	-1	X	10132908	10132911
Three additional base positions from the 3'-end	Arg_ACG	1	X	10145169	10145172
Three additional base positions from the 3'-end	Arg_ACG	-1	X	10148586	10148589
Three additional base positions from the 3'-end	Glu_TTC	-1	X	10666955	10666958
Three additional base positions from the 3'-end	Glu_TTC	1	X	10667601	10667604
Three additional base positions from the 3'-end	Arg_ACG	-1	X	10731920	10731923
Three additional base positions from the 3'-end	Glu_TTC	-1	X	10821258	10821261
Three additional base positions from the 3'-end	Gly_TCC	1	X	10963833	10963836
Three additional base positions from the 3'-end	Leu_TAG	1	X	11095688	11095691
Three additional base positions from the 3'-end	Leu_AAG	-1	X	11162849	11162852
Three additional base positions from the 3'-end	Leu_AAG	-1	X	11163288	11163291
Three additional base positions from the 3'-end	Arg_ACG	-1	X	11252521	11252524
Three additional base positions from the 3'-end	Asn_GTT	1	X	11645688	11645691
Three additional base positions from the 3'-end	Leu_TAA	-1	X	11757760	11757763
Three additional base positions from the 3'-end	Arg_TCG	-1	X	11806516	11806519
Three additional base positions from the 3'-end	Gln_TTG	-1	X	11872169	11872172
Three additional base positions from the 3'-end	Gln_CTG	1	X	11872509	11872512
Three additional base positions from the 3'-end	Thr_CGT	1	X	11933731	11933734
Three additional base positions from the 3'-end	Thr_CGT	-1	X	11935008	11935011
Three additional base positions from the 3'-end	His_GTG	-1	X	11993449	11993452
Three additional base positions from the 3'-end	Lys_CTT	-1	X	12106075	12106078
Three additional base positions from the 3'-end	Lys_CTT	1	X	12166224	12166227
Three additional base positions from the 3'-end	Lys_CTT	-1	X	12167038	12167041
Three additional base positions from the 3'-end	Arg_TCG	-1	X	12337926	12337929
Three additional base positions from the 3'-end	Leu_CAA	-1	X	12347935	12347938
Three additional base positions from the 3'-end	Glu_TTC	-1	X	12367570	12367573
Three additional base positions from the 3'-end	Ala_AGC	-1	X	12458104	12458107
Three additional base positions from the 3'-end	Thr_TGT	1	X	12563790	12563793
Three additional base positions from the 3'-end	Ile_TAT	1	X	12661176	12661179
Three additional base positions from the 3'-end	Tyr_GTA	1	X	12665240	12665243
Three additional base positions from the 3'-end	Val_AAC	1	X	12843690	12843693
Three additional base positions from the 3'-end	Pro_TGG	-1	X	12854766	12854769
Three additional base positions from the 3'-end	Ser_TGA	1	X	12889951	12889954
Three additional base positions from the 3'-end	Ala_CGC	-1	X	12890211	12890214
Three additional base positions from the 3'-end	Gln_TTG	1	X	12937702	12937705
Three additional base positions from the 3'-end	Gln_TTG	1	X	12938183	12938186
Three additional base positions from the 3'-end	Leu_AAG	1	X	12942469	12942472
Three additional base positions from the 3'-end	Leu_CAG	-1	X	13038887	13038890
Three additional base positions from the 3'-end	Ser_CGA	-1	X	13046293	13046296
Three additional base positions from the 3'-end	Ser_AGA	1	X	13046590	13046593
Three additional base positions from the 3'-end	Gly_GCC	-1	X	13121187	13121190
Three additional base positions from the 3'-end	Thr_AGT	1	X	13261205	13261208
Three additional base positions from the 3'-end	Tyr_GTA	-1	X	13264610	13264613
Three additional base positions from the 3'-end	Thr_AGT	-1	X	13283564	13283567
Three additional base positions from the 3'-end	Tyr_GTA	-1	X	13284693	13284696
Three additional base positions from the 3'-end	Thr_AGT	1	X	13293194	13293197
Three additional base positions from the 3'-end	Val_TAC	1	X	13307037	13307040

Three additional base positions from the 3'-end	Pro_TGG	1	X	13308032	13308035
Three additional base positions from the 3'-end	Ile_AAT	-1	X	13415048	13415051
Three additional base positions from the 3'-end	Ile_AAT	1	X	13435971	13435974
Three additional base positions from the 3'-end	Ser_GCT	1	X	13442656	13442659
Three additional base positions from the 3'-end	Leu_CAA	-1	X	13443314	13443317
Three additional base positions from the 3'-end	Glu_CTC	1	X	13448680	13448683
Three additional base positions from the 3'-end	Ser_GCT	-1	X	13448801	13448804
Three additional base positions from the 3'-end	Pro_TGG	-1	X	13511896	13511899
Three additional base positions from the 3'-end	Thr_TGT	1	X	13653729	13653732
Three additional base positions from the 3'-end	Lys_CTT	-1	X	13655843	13655846
Three additional base positions from the 3'-end	Leu_CAA	1	X	13665603	13665606
Three additional base positions from the 3'-end	Lys_CTT	1	X	13714891	13714894
Three additional base positions from the 3'-end	Lys_CTT	1	X	13716496	13716499
Three additional base positions from the 3'-end	Lys_CTT	1	X	13717198	13717201
Three additional base positions from the 3'-end	Lys_CTT	1	X	13729908	13729911
Three additional base positions from the 3'-end	Lys_CTT	1	X	13730360	13730363
Three additional base positions from the 3'-end	Lys_CTT	1	X	13730781	13730784
Three additional base positions from the 3'-end	Lys_CTT	1	X	13781644	13781647
Three additional base positions from the 3'-end	Lys_CTT	1	X	13782253	13782256
Three additional base positions from the 3'-end	Gly_TCC	-1	X	13886297	13886300
Three additional base positions from the 3'-end	Gly_TCC	1	X	14010255	14010258
Three additional base positions from the 3'-end	Glu_CTC	1	X	14015946	14015949
Three additional base positions from the 3'-end	Lys_CTT	1	X	14061677	14061680
Three additional base positions from the 3'-end	Lys_CTT	-1	X	14062366	14062369
Three additional base positions from the 3'-end	Lys_TTT	-1	X	14063873	14063876
Three additional base positions from the 3'-end	Arg_CCT	1	X	14115694	14115697
Three additional base positions from the 3'-end	His_GTG	1	X	14135047	14135050
Three additional base positions from the 3'-end	His_GTG	-1	X	14135235	14135238
Three additional base positions from the 3'-end	His_GTG	1	X	14135876	14135879
Three additional base positions from the 3'-end	His_GTG	-1	X	14136051	14136054
Three additional base positions from the 3'-end	His_GTG	1	X	14136692	14136695
Three additional base positions from the 3'-end	His_GTG	-1	X	14136866	14136869
Three additional base positions from the 3'-end	Asp_GTC	-1	X	14161953	14161956
Three additional base positions from the 3'-end	Ser_AGA	1	X	14162227	14162230
Three additional base positions from the 3'-end	Trp_CCA	-1	X	14228847	14228850
Three additional base positions from the 3'-end	Pro_TGG	-1	X	14437799	14437802
Three additional base positions from the 3'-end	Pro_TGG	1	X	14438103	14438106
Three additional base positions from the 3'-end	Lys_CTT	-1	X	14478803	14478806
Three additional base positions from the 3'-end	Lys_CTT	-1	X	14486137	14486140
Three additional base positions from the 3'-end	Ile_AAT	1	X	14679164	14679167
Three additional base positions from the 3'-end	Cys_GCA	1	X	14804879	14804882
Three additional base positions from the 3'-end	Arg_TCT	-1	X	15146488	15146491
Three additional base positions from the 3'-end	Gly_TCC	-1	X	15200744	15200747
Three additional base positions from the 3'-end	Gly_TCC	1	X	15201009	15201012
Three additional base positions from the 3'-end	Val_AAC	1	X	15236127	15236130
Three additional base positions from the 3'-end	Lys_CTT	-1	X	15241282	15241285
Three additional base positions from the 3'-end	Thr_CGT	1	X	15248974	15248977
Three additional base positions from the 3'-end	Ile_AAT	1	X	15297475	15297478

Three additional base positions from the 3'-end	Glu_TTC	1	X	15368677	15368680
Three additional base positions from the 3'-end	Leu_CAG	1	X	15742065	15742068
Three additional base positions from the 3'-end	Pro_TGG	-1	X	15747731	15747734
Three additional base positions from the 3'-end	Pro_TGG	1	X	15748034	15748037
Three additional base positions from the 3'-end	Pro_TGG	-1	X	15748244	15748247
Three additional base positions from the 3'-end	Pro_TGG	1	X	15751423	15751426
Three additional base positions from the 3'-end	Ser_AGA	-1	X	15769937	15769940
Three additional base positions from the 3'-end	Ser_CGA	-1	X	15773259	15773262
Three additional base positions from the 3'-end	Ser_AGA	1	X	15773540	15773543
Three additional base positions from the 3'-end	Pro_TGG	-1	X	15796551	15796554
Three additional base positions from the 3'-end	Pro_TGG	1	X	15796858	15796861
Three additional base positions from the 3'-end	Ser_TGA	-1	X	16042532	16042535
Three additional base positions from the 3'-end	Tyr_GTA	-1	X	16208187	16208190
Three additional base positions from the 3'-end	Tyr_GTA	-1	X	16208749	16208752
Three additional base positions from the 3'-end	Tyr_GTA	-1	X	16209152	16209155
Three additional base positions from the 3'-end	Tyr_GTA	-1	X	16209693	16209696
Three additional base positions from the 3'-end	Lys_TTT	-1	X	16217819	16217822
Three additional base positions from the 3'-end	Glu_CTC	-1	X	16271219	16271222
Three additional base positions from the 3'-end	Glu_CTC	1	X	16273765	16273768
Three additional base positions from the 3'-end	Glu_CTC	1	X	16274496	16274499
Three additional base positions from the 3'-end	Glu_CTC	-1	X	16284246	16284249
Three additional base positions from the 3'-end	Glu_CTC	-1	X	16285218	16285221
Three additional base positions from the 3'-end	Val_AAC	1	X	16298341	16298344
Three additional base positions from the 3'-end	Val_AAC	1	X	16299393	16299396
Three additional base positions from the 3'-end	Val_AAC	1	X	16300063	16300066
Three additional base positions from the 3'-end	Val_AAC	1	X	16300589	16300592
Three additional base positions from the 3'-end	Glu_CTC	1	X	16378112	16378115
Three additional base positions from the 3'-end	Met_CAT	1	X	16441145	16441148
Three additional base positions from the 3'-end	Arg_TCG	1	X	16461476	16461479
Three additional base positions from the 3'-end	Phe_GAA	-1	X	16503067	16503070
Three additional base positions from the 3'-end	Phe_GAA	1	X	16522768	16522771
Three additional base positions from the 3'-end	Phe_GAA	1	X	16523343	16523346
Three additional base positions from the 3'-end	Phe_GAA	1	X	16523922	16523925
Three additional base positions from the 3'-end	Phe_GAA	1	X	16551510	16551513
Three additional base positions from the 3'-end	Glu_CTC	1	X	16558940	16558943
Three additional base positions from the 3'-end	Val_CAC	1	X	16590394	16590397
Three additional base positions from the 3'-end	Val_CAC	-1	X	16590854	16590857
Three additional base positions from the 3'-end	Asp_GTC	-1	X	16604643	16604646
Three additional base positions from the 3'-end	Asp_GTC	-1	X	16617815	16617818
Three additional base positions from the 3'-end	Asp_GTC	-1	X	16618219	16618222
Three additional base positions from the 3'-end	Asp_GTC	-1	X	16619256	16619259
Three additional base positions from the 3'-end	His_GTG	-1	X	16634753	16634756
Three additional base positions from the 3'-end	Ser_GCT	-1	X	16689908	16689911
Three additional base positions from the 3'-end	Gly_GCC	-1	X	16928194	16928197
Three additional base positions from the 3'-end	Val_AAC	1	X	17611285	17611288
Three additional base positions from the 3'-end	Val_AAC	-1	X	17611423	17611426

Supplementary Table S8. Northern probes

probe_id	source_seq_id	target	probe
Asp_GTC_5	Chr_V_29_Asp_GTC	TCCTCGGTAGTATAGTGGTGAGTATCCGCG	CGCGGATACTCACCACTATACTACCGAGGA
Asp_GTC_3	Chr_V_29_Asp_GTC	AGACCCGGGTTCAATTCCCGGCCG	CGGCCGGGAATTGAACCCGGGTCT
Gln_TTG_5	Chr_X_60_Gln_TTG	GGTTCCATGGTGTAGCGGTTAGCACTCAGG	CCTGAGTGCTAACCGCTACACCATGGAACC
Gln_TTG_3	Chr_X_60_Gln_TTG	ACCCGAGTTCAAATCTCGGTGGAACCTCCA	TGGAGGTTCCACCGAGATTTGAACTCGGGT
Lys_CTT_5	Chr_III_26_Lys_CTT	GCCCGGTTAGCTCAGTCGGTAGAGCACC	GGTGCTCTACCGACTGAGCTAACCGGGC
Lys_CTT_3	Chr_III_26_Lys_CTT	TCGCGGGTTCGAGCCCCGCATT	AATGCGGGGCTCGAACCCGCGA
Ser_AGA_5	Chr_II_7_Ser_AGA	GCAGTCATGTCCGAGTGGTTAAGGAGATTG	CAATCTCCTTAACCACTCGGACATGACTGC
Ser_AGA_3	Chr_II_7_Ser_AGA	GTAGGTTCGAATCCTGCTGACTGCGCCA	TGGCGCAGTCAGCAGGATTCGAACCTAC
